# Response of the replisome to araA and araC suggests a mechanism for arabinosyl nucleoside tolerance

**DOI:** 10.1038/s44318-026-00901-1

**Published:** 2026-08-27

**Authors:** Harry Orrin, Joseph T P Yeeles

**Affiliations:** 1 MRC Laboratory of Molecular Biology, Cambridge Biomedical Campus, Cambridge, UK; 2 Present Address: MRC Human Genetics Unit, Institute of Genetics and Cancer, University of Edinburgh, Western General Hospital, Edinburgh, UK

**Keywords:** DNA Replication, Recombination & Repair

## Abstract

Arabinosyl (ara) nucleoside analogues such as vidarabine (araA) and cytarabine (araC) are antimetabolites known to inhibit DNA polymerases in isolation. However, their effects on DNA synthesis catalysed by the multi-protein replisome remain unknown. Here, we uncover a central role for the lagging-strand DNA polymerase, Pol δ, in tolerating these drugs. Upon DNA synthesis inhibition by ara nucleotides, Pol δ replaces the canonical leading-strand DNA polymerase, Pol ε, to synthesise and proofread nascent leading strands in vitro and in vivo, with these Pol δ activities limiting helicase-polymerase uncoupling and intra-S checkpoint activation. Removal of ara analogues from nascent strands by DNA polymerase proofreading is likely the primary resistance mechanism against these drugs since a template-embedded analogue can be bypassed by the replisome. This study defines the initial response of the eukaryotic replisome to araA and araC and may have implications for how resistance arises against them, and other nucleoside analogue chemotherapeutics, in the clinic.

## Synopsis

Arabinosyl nucleoside analogues are chemotherapeutic antimetabolites inhibiting isolated DNA polymerases. Here, their use on reconstituted full replisomes shows that the canonical lagging-strand DNA polymerase Pol δ can take over leading strand replication, with implications for drug tolerance evolution.

- Pol δ plays a central role in the response of the eukaryotic replisome to arabinosyl nucleoside analogues.
- DNA synthesis inhibition by arabinosyl analogues causes nascent leading strands to become uncoupled from the CMG helicase.
- This enables Pol δ to replace Pol ***ε*** (the canonical leading-strand DNA polymerase) and synthesise and proofread nascent leading strands.
- These Pol δ activities limit helicase-polymerase uncoupling and intra-S-checkpoint activation.
- The extent of Pol δ usage on the leading strand is shaped by both drug dose and the ability of Pol ***ε*** to proofread.

## Abstract (web summary)

Upon DNA synthesis inhibition by arabinosyl nucleoside analogues, the canonical lagging-strand DNA polymerase Pol δ synthesises and proofreads nascent leading strands, revealing replisome plasticity.

## Graphical abstract

**Figure:**
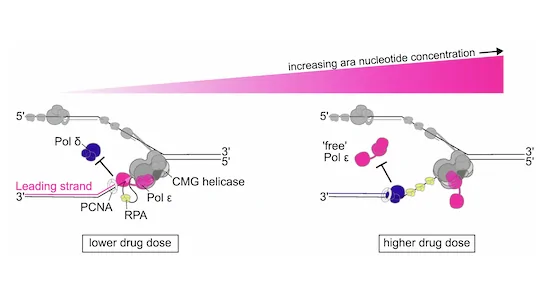

## Introduction

**Figure 1:**
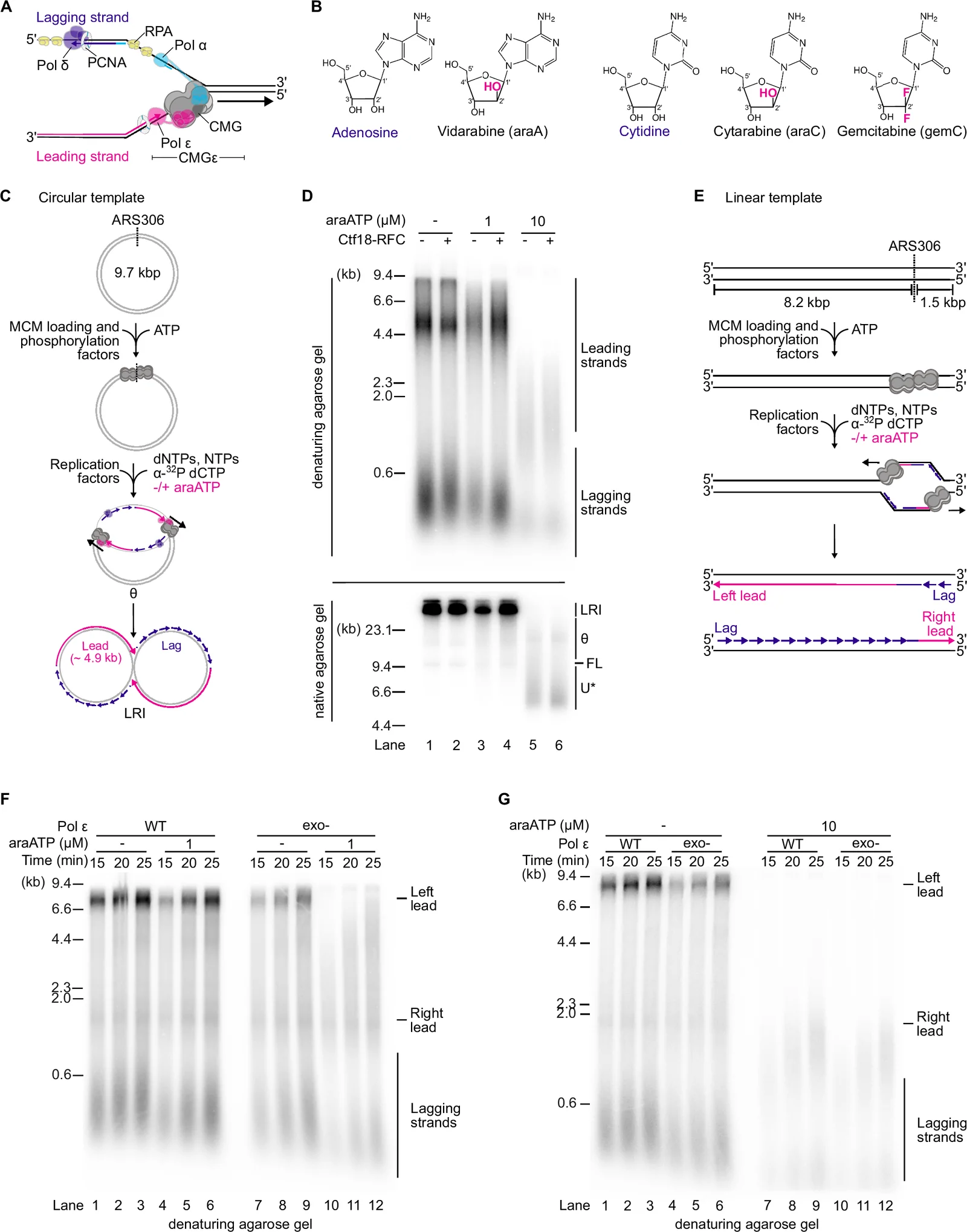
AraATP causes leading-strand synthesis to be uncoupled from the CMG helicase. (**A**) Schematic illustrating the organisation of the eukaryotic replisome (several factors omitted for simplicity). CMGε, CMG + Pol ε. (**B**) Structures of adenosine and cytidine nucleoside analogues. Arabinosyl (ara) analogues contain an arabinose sugar moiety characterised by an ara hydroxyl at the 2’ position whereas the related analogue gemcitabine contains two fluorines (pink). (**C**) Schematic representation of an in vitro replication reaction on 9.7 kbp circular dsDNA template containing the ARS306 replication origin. Predicted leading- (pink) and lagging-strand (blue) products are shown (see “Methods” for further details). θ, see main text. LRI late replication intermediate. (**D**) Denaturing (top) and native gel (bottom) autoradiography of replication reactions performed as in (**C**). Reactions were quenched after 10 min. FL full length. U*, see main text. The native gel was overexposed to aid visualisation of weaker U* species. (**E**) Schematic representation of an in vitro replication reaction on an AhdI-linearised version of dsDNA template in (**C**). (**F**, **G**) Denaturing gel autoradiography of replication reactions performed as in (**E**). WT wild-type. See Appendix Fig. S5 for lane quantification. Source data are available online for this figure.

To ensure faithful inheritance of the genome following cell division, each chromosome must be duplicated accurately and completely. Failure to achieve this can cause enhanced replication stress leading to genomic instability, a major hallmark of cancer (Hanahan and Weinberg, 2011). DNA replication is carried out by a multi-protein complex termed the ‘replisome’ (Fig. 1A). In eukaryotes, this comprises a central Cdc45-Mcm2-7-GINS (CMG) helicase decorated with various factors including DNA polymerases Pol α-primase (Pol α) and Pol ε. During S-phase of the cell cycle, DNA replication initiates at specific locations throughout the genome termed ‘replication origins’. DNA is first unwound by two CMG helicases giving rise to two replication forks emanating from each origin. After sufficient template unwinding, Pol α initiates DNA replication by synthesising a short RNA-DNA primer (Pellegrini, 2012; Baranovskiy et al, 2022; Jones et al, 2023). At this primer-template junction, the Rfc1-RFC complex loads the processivity clamp, PCNA (Maga et al, 2000), enabling Pol δ to extend the primer across the replication origin, displacing the single-stranded (ss)DNA-binding protein, replication protein A (RPA). The nascent strand is then handed over to Pol ε which associates with the CMG helicase (CMGε) to catalyse the bulk of continuous leading-strand synthesis (Fig. 1A; Garbacz et al, 2018; Aria and Yeeles, 2019; Pursell et al, 2007; Clausen et al, 2015; Daigaku et al, 2015; Reijns et al, 2015; Koyanagi et al, 2022). Meanwhile, Pol δ carries out the bulk of discontinuous lagging-strand replication by extending primers deposited by Pol α (Nick McElhinny et al, 2008; Clausen et al, 2015; Daigaku et al, 2015; Reijns et al, 2015; Koyanagi et al, 2022).

Numerous anticancer agents act by inhibiting DNA synthesis. One type of such agents is purine and pyrimidine nucleoside analogue chemotherapeutics, a subset of which includes arabinosyl (ara) analogues, characterised by their arabinose sugar moiety (Seley-Radtke and Yates, 2018; Yates and Seley-Radtke, 2019; Fig. 1B). These act as first-line treatments against a wide-range of solid and blood cancers (Tsesmetzis et al, 2018). For example, the ara cytidine analogue cytarabine (araC) is a mainstay for treatment against acute myeloid leukaemia (Khwaja et al, 2016; Dombret and Gardin, 2016) and has been in clinical use for over 55 years (Jordheim et al, 2013). By contrast, whilst initially developed as an anticancer drug, the ara adenosine analogue vidarabine (araA) is now used primarily as an antiviral agent owing to its rapid deamination in human cells (De Clercq and Li, 2016). Gemcitabine (gemC), a non-ara cytidine analogue, is also used to treat pancreatic and lung cancers as well as sarcomas (Ciccolini et al, 2011; Fig. 1B).

Following cellular uptake and phosphorylation to triphosphates, ara nucleotides are thought to block DNA replication. Evidence for this mode-of-action stems primarily from in vitro studies demonstrating that the analogues can non-specifically inhibit multiple targets including a range of DNA polymerases, DNA ligase and ribonucleotide reductase (Dicioccio and Srivastava, 1977; Yang et al, 1992; White et al, 1982; Parker et al, 1988; Tsuda et al, 2017; Chang et al, 2024; Shen et al, 2024; Richardson et al, 2004; Catapano et al, 1993; Huang et al, 1990; Tseng et al, 1982; Prakasha Gowda et al, 2010; Fowler et al, 2008; Garcia-Diaz et al, 2010; Kufe and Major, 1982; Harrington and Perrino, 1995; Parker and Cheng, 1987; Perrino and Mekosh, 1992; Kufe et al, 1980; Rashbaum and Cozzarelli, 1976). These analogues are incorporated into the nascent DNA strand but, despite having a 3’-hydroxyl group, subsequent extension from them is inefficient, essentially rendering them chain terminators (Tsuda et al, 2017; Huang et al, 1990; Holzer et al, 2019). However, many of the studies of how nucleoside analogues operate were performed before the CMG complex was characterised as the helicase responsible for unwinding duplex DNA (Moyer et al, 2006; Gambus et al, 2006), and before the division of labour of the replicative polymerases to either the leading or lagging strands was fully established (Pursell et al, 2007; Nick McElhinny et al, 2008; Clausen et al, 2015; Daigaku et al, 2015; Reijns et al, 2015; Koyanagi et al, 2022). As such, whilst the notion that these analogues inhibit a wide range of DNA polymerases in isolation is well appreciated, it is not understood whether specific polymerases are differently affected when carrying out DNA synthesis in concert as part of the replisome.

In this study, we set out to understand how the replisome responds to inhibition of DNA synthesis by ara nucleoside analogues. We show that in response to DNA synthesis inhibition by araA, Pol δ exchanges with Pol ε to synthesise and proofread nascent leading strands in vitro and in vivo. In doing so, Pol δ suppresses the extent of separation between the 3’ end of the nascent leading strand and the CMG helicase (helicase-polymerase uncoupling) and thus limits DNA damage checkpoint activation. By manipulating the proofreading activities of these enzymes, we demonstrate that removal of araA and araC from nascent leading strands is critical for efficient synthesis rates and is likely the primary resistance mechanism towards these drugs since the replisome bypasses a single template-embedded analogue. Together, this study defines how the replisome tolerates araA and araC and may have implications for how resistance arises against them, and other nucleoside analogue chemotherapeutics, in the clinic.

## Results

### AraATP causes CMG to be uncoupled from leading-strand synthesis

Since only the elongation stage of human DNA replication has been reconstituted (Baris et al, 2022), we utilised a previously-established in vitro replication system with purified budding yeast proteins (Yeeles et al, 2015, 2017) to examine the effects of ara nucleotides on replisomes during both origin activation and elongation. This system gives rise to two replisomes assembled and activated at a replication origin (e.g. ARS306) that carry out leading- and lagging-strand DNA synthesis at in vivo rates. Using this system, we performed reactions with a circular 9.7 kbp double-stranded (ds)DNA template in either the presence or absence of araATP (Fig. 1C). The addition of radiolabelled dCTP enables nascent products to be visualised by autoradiography following deproteinization and denaturing or native agarose gel electrophoresis. Inspection of the denaturing gel in the absence of ara nucleotides revealed leading-strand products corresponding to approximately half the template length (~4.9 kb), and short lagging-strand products (~0.6 kb) synthesised by 10 min (Fig. 1D, denaturing, lanes 1–2). These product lengths are expected, given that termination-supporting factors (Pif1/Rrm3) and Okazaki fragment maturation factors (Fen1/Cdc9) were omitted from the reactions (Deegan et al, 2019). Addition of 1 μM araATP had little effect on synthesis, but adding 10 μM slowed leading-strand synthesis and resulted in shorter lagging-strand products (Fig. 1D, denaturing, compare lanes 3–4 with 5–6). This effect was not rescued by addition of the clamp loader Ctf18-RFC which promotes PCNA loading on the leading strand (Liu et al, 2020) (Fig. 1D, denaturing, compare lanes 5 and 6). In line with this, upon addition of 10 μM araATP slow migrating products indicative of late replication intermediates (LRIs) were absent and there was an increase in θ intermediates (Fig. 1C) in the native gel, which preserves the structures of replication intermediates (Fig. 1D, native, compare lanes 1–4 with 5–6). The most notable difference, however, was the presence of a 10 μM araATP-dependent fast-migrating species (Fig. 1D, native, lanes 5–6). This species (U*) occurs when a stretch of ssDNA forms between the 3’ end of the nascent leading strand and the CMG helicase which continues to unwind dsDNA (Devbhandari and Remus, 2020). Following deproteinization, the unwound strands reanneal leading to a negatively supercoiled plasmid which migrates faster through the native gel than a standard θ intermediate (Devbhandari and Remus, 2020). We conclude that addition of 10 μM araATP causes leading-strand synthesis to become uncoupled from CMG helicase DNA unwinding, and that this is not reduced in the presence of Ctf18-RFC.

### Loss of Pol ε proofreading enhances leading-strand uncoupling by araATP

**Figure EV1:**
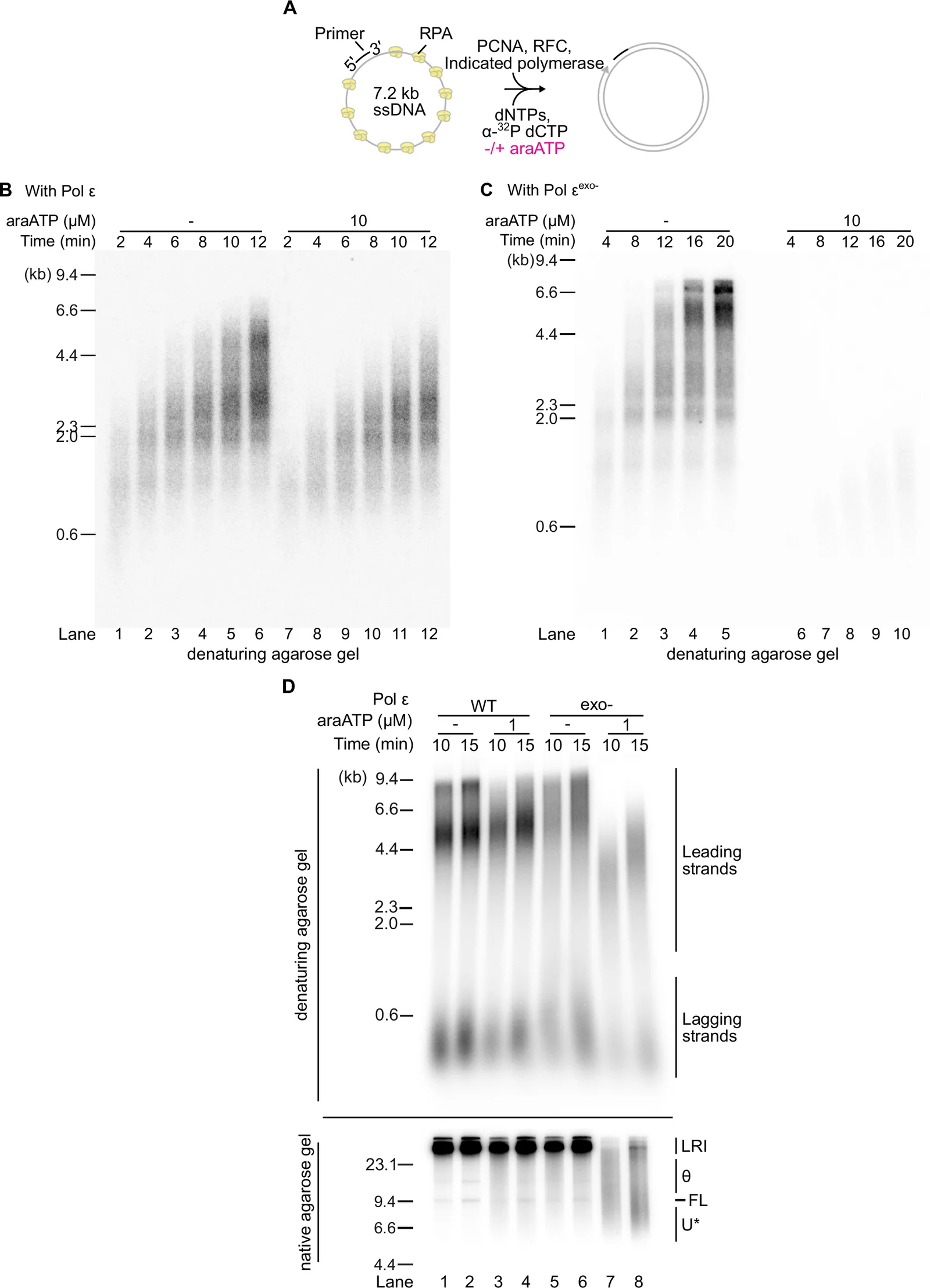
Pol ε proofreading restricts araATP-induced helicase-polymerase uncoupling. Related to Fig. 1. (**A**) Schematic representation of in vitro primer extension assay on singly primed ssDNA template. (**B**, **C**) Denaturing gel autoradiography of primer extension reactions performed with the indicated polymerase as in (**A**). (**D**) Denaturing (top) and native gel (bottom) autoradiography of replication reactions performed as in Fig. 1C. The native gel was overexposed to aid visualisation of weaker U* species. Source data are available online for this figure.

The lack of synthesis inhibition at 1 μM araATP (Fig. 1D, lanes 3–4) led us to explore possible tolerance mechanisms that enable the replisome to resist helicase-polymerase uncoupling caused by these drugs. Previous work has shown that proofreading-defective human Pol ε is more sensitive than wild-type to inhibition by araCTP (Tsuda et al, 2017). Consistent with this, primer extension reactions on a singly primed ssDNA template (Fig. EV1A) demonstrate that purified proofreading-defective budding yeast Pol ε (Pol ε^exo-^, D290A, E292A; Guilliam and Yeeles, 2021) is markedly more sensitive to inhibition by araATP than wild-type (compare Fig. EV1B with EV1C). We then tested the effect of losing Pol ε proofreading in replication reactions with a linear dsDNA template. Replication of this template gives rise to two differentially sized leading strands (8.2 kb and 1.5 kb) each synthesised by a single replication fork population (Fig. 1E). As such, the length distribution of leading strands is minimised, making it easier to detect differences in synthesis rates compared with reactions using a circular template. Upon addition of 1 μM araATP, leading-strand synthesis rates were minimally affected in reactions with Pol ε (Fig. 1F, compare lanes 1–3 with 4–6). However, leading-strand synthesis rates were significantly attenuated upon 1  μM araATP addition in reactions with Pol ε^exo-^, with the bulk length of leading-strand products being ~3 kb shorter at 15 min relative to no drug conditions (Fig. 1F, compare lanes 7–9 with 10–12). Moreover, the extent of helicase-polymerase uncoupling increased under similar conditions (Fig. EV1D). Surprisingly, and in contrast to Pol ε primer extension data (Fig. EV1B,C; Tsuda et al, 2017), repeating the above experiment with 10 μM araATP revealed that synthesis rates in reactions containing either Pol ε or Pol ε^exo-^ were almost identical (Fig. 1G, compare lanes 7–9 with 10–12). We conclude that under low araATP concentrations (1 μM) uncoupling of leading-strand synthesis from helicase unwinding is minimised by Pol ε proofreading, however this activity has a less significant effect at higher drug concentrations (10 μM).

### Pol δ carries out leading-strand synthesis in the presence of araATP

**Figure EV2:**
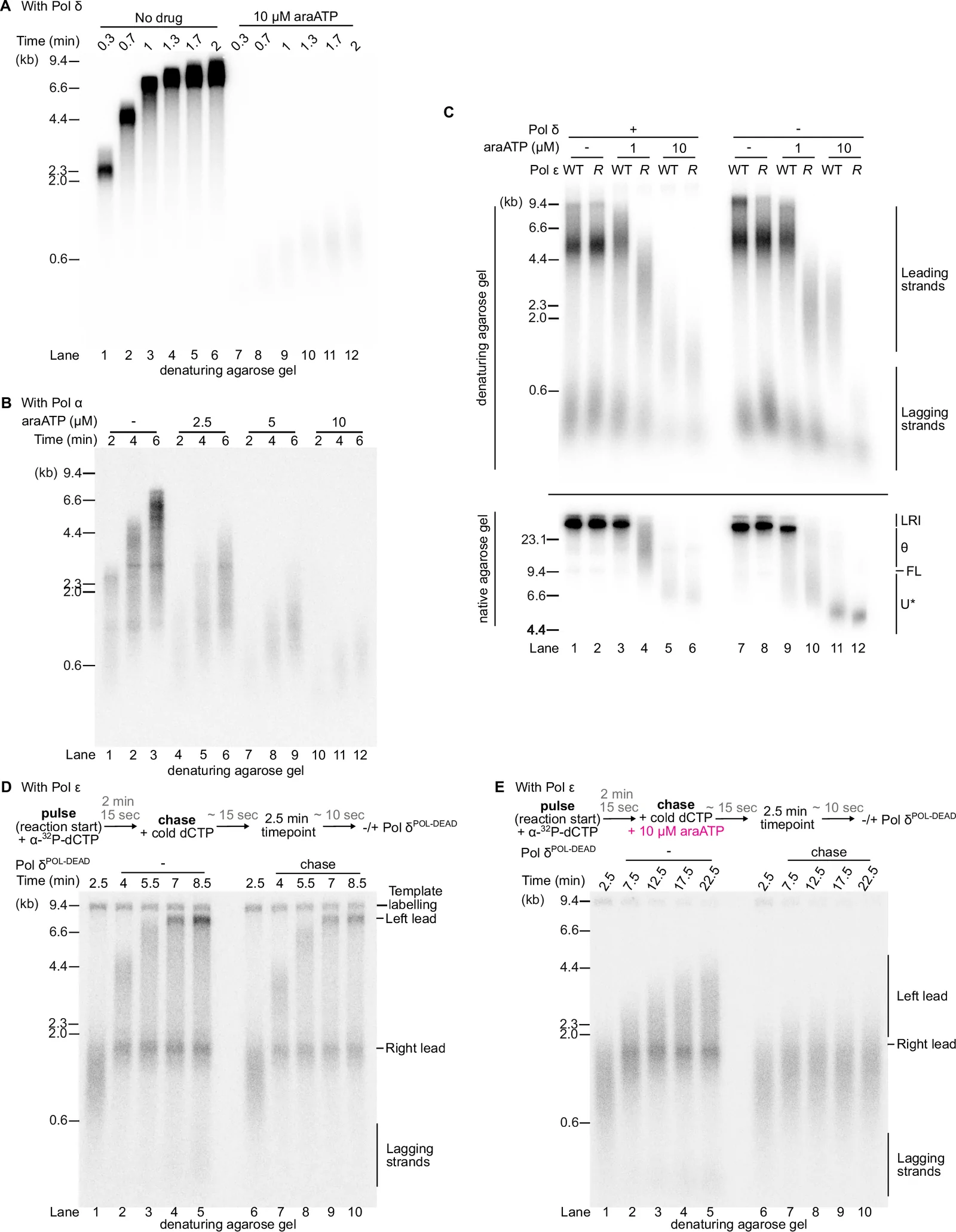
Additional evidence for the role of Pol δ on the leading strand in response to araATP. Related to Fig. 2. (**A**) Denaturing gel autoradiography of primer extension reaction performed as in Fig. EV1A with Pol δ. The image was overexposed to aid visualisation of weaker synthesis products (lanes 7–12). (**B**) Denaturing gel autoradiography of primer extension reaction performed as in (A) except with Pol α and without RFC. (**C**) Denaturing (top) and native gel (bottom) autoradiography of replication reactions performed as in Fig. 1C. Reactions were quenched after 10 min. Pol ε R = Pol ε^P301R^. The native gel was overexposed to aid visualisation of weaker U* species. (**D**, **E**) Pulse-chase replication reactions performed on AhdI-linearised dsDNA template as indicated (and see “Methods”) and analysed by denaturing gel autoradiography. Where indicated, araATP was added during the chase to a final concentration of 10 μM after all components were added. Source data are available online for this figure.

The reconstituted replication reactions described so far contain three B-family polymerases Pol α, Pol δ, and Pol ε; all of which are inhibited by araATP (Figs. EV1B and EV2A,B). Under unperturbed conditions, Pol ε catalyses the bulk of leading-strand replication (Pursell et al, 2007). This strand bias is mainly attributed to its numerous interactions with the CMG helicase which unwinds dsDNA as it encircles the leading-strand template (Langston et al, 2014; Georgescu et al, 2014; Goswami et al, 2018), giving rise to processive leading-strand synthesis catalysed by CMGε (Fig. 1A).

**Figure 2:**
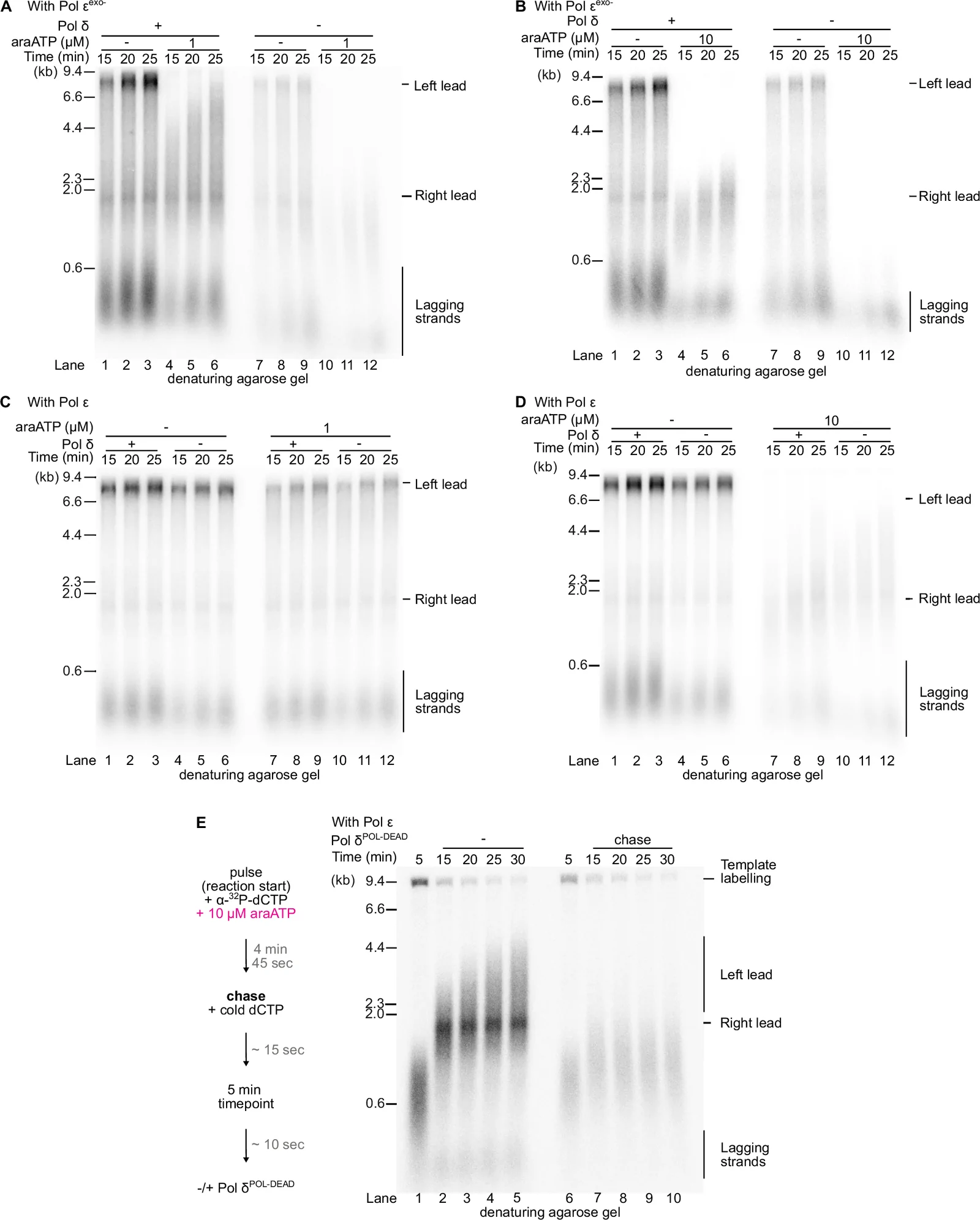
Pol δ catalyses leading- and lagging-strand synthesis in the presence of araATP. (**A**–**D**) Denaturing gel autoradiography of replication reactions performed on AhdI-linearised dsDNA template. See Appendix Fig. S5 for lane quantification. (**E**) Pulse-chase replication reaction performed on AhdI-linearised dsDNA template as indicated (and see “Methods”) and analysed by denaturing gel autoradiography. AraATP was added during the pulse to a final concentration of 10 μM after all components were added. Template labelling (a replisome-independent event) is indicated. See Appendix Fig. S5 for quantification of maximal leading-strand synthesis rates. Source data are available online for this figure.

Since Pol ε proofreading does not significantly affect replication rate in the presence of 10  μM araATP (Fig. 1G), and Pol α cannot support efficient leading-strand synthesis under these conditions (Yeeles et al, 2017), we wondered whether the uncoupling caused by ara nucleotides (Figs. 1D and EV1D) allows Pol δ to take over leading-strand synthesis. To test this, we first performed replication reactions using either Pol ε^exo-^ or Pol ε in the presence or absence of Pol δ. With Pol ε^exo-^, omitting Pol δ enhanced the inhibition of leading-strand synthesis at 1 μM araATP and almost completely abolished synthesis at 10 μM araATP (Fig. 2A,B compare lanes 4–6 with 10–12). We additionally observed this effect in similar experiments using an exonuclease-defective Pol ε mutant (Pol ε^P301R^) that is orthologous to the human Pol ε P286R variant associated with a subset of colorectal and endometrial cancers (Kane and Shcherbakova, 2014) (Fig. EV2C).

Omitting Pol δ in reactions containing Pol ε had no effect on leading-strand synthesis at 1 μM araATP (Fig. 2C, compare lanes 7–9 with 10–12) presumably because Pol ε proofreading minimises helicase-polymerase uncoupling at this drug concentration (Figs. 1D and EV1D). Surprisingly, loss of Pol δ also had little if any effect on leading-strand synthesis in Pol ε reactions at 10 μM araATP (Fig. 2D, compare lanes 7–9 with 10–12) where helicase-polymerase uncoupling is expected (Fig. 1D). One possible explanation for this lack of difference could be that Pol δ does not contribute to leading-strand synthesis in the presence of drug when Pol ε can proofread. Alternatively, Pol δ may carry out leading-strand synthesis but the time taken to remove ara nucleotides from nascent strands may limit the synthesis rates of Pol ε and Pol δ such that they appear equal. To distinguish between these possibilities, we performed a pulse-chase replication reaction using a Pol δ mutant lacking polymerase activity (Pol δ^POL-DEAD^; Aria and Yeeles, 2019). In this reaction, replication is initiated without Pol δ at a low dCTP concentration (pulse) before an excess of unlabelled dCTP is added (chase) to enable the monitoring of a subset of elongating leading strands. This is then followed immediately by addition of Pol δ^POL-DEAD^ where indicated. Under unperturbed conditions, addition of Pol δ^POL-DEAD^ had little effect on leading-strand elongation owing to processive synthesis catalysed by CMGε (Aria and Yeeles, 2019; and Fig. EV2D). However, in the presence of 10 μM araATP (added during the pulse), addition of Pol δ^POL-DEAD^ completely inhibited further DNA synthesis (Fig. 2E, compare lanes 1–5 with 6–10). We observed the same trend when 10 μM araATP was added later in the chase, presumably after synthesis by CMGε becomes established (Fig. EV2E).

Together, these experiments support the notion that Pol δ catalyses leading-strand synthesis at most, if not all, replication forks in the presence of 10 μM araATP in vitro. They further show that Pol δ contributes to leading-strand synthesis at lower drug concentrations when Pol ε proofreading is compromised.

### Pol δ proofreads nascent leading strands to remove incorporated araA

**Figure 3:**
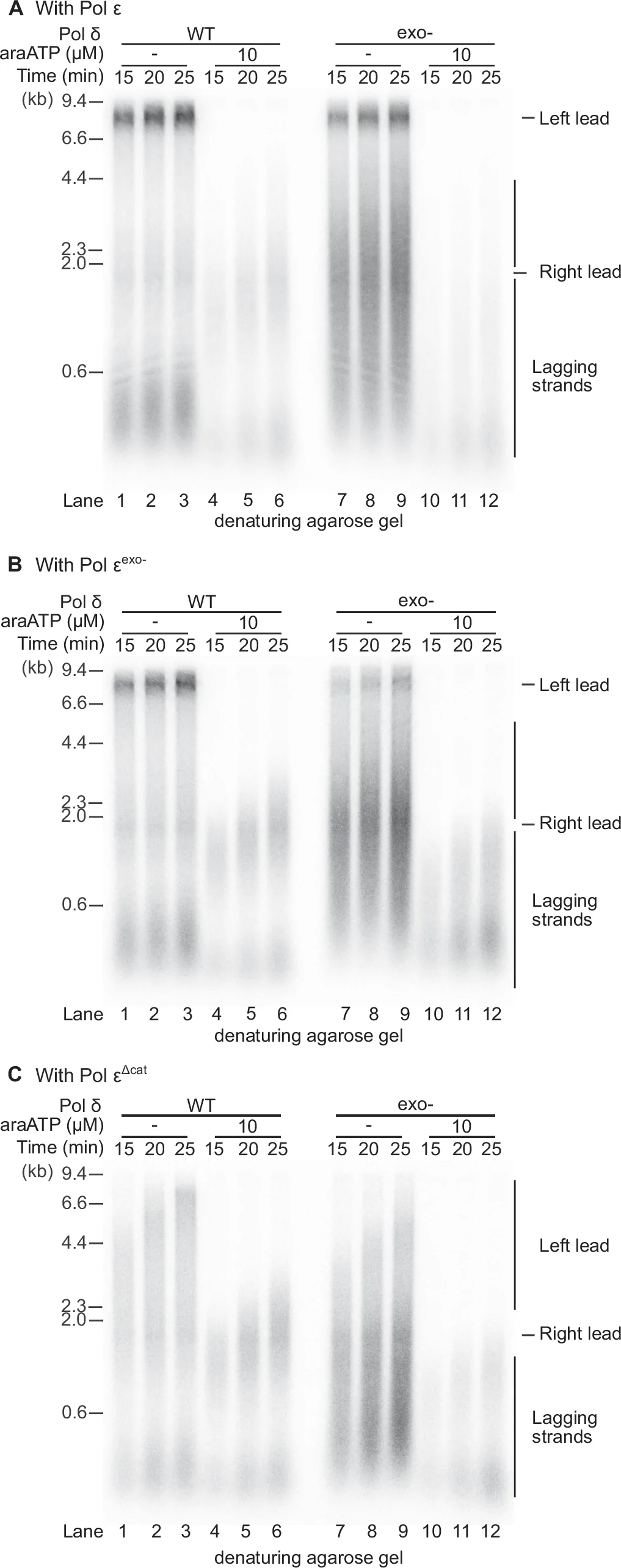
Pol δ removes incorporated araA from nascent leading strands. (**A**–**C**) Denaturing gel autoradiography of replication reactions performed on AhdI-linearised dsDNA template. See Appendix Fig. S5 for lane quantification. Source data are available online for this figure.

**Figure EV3:**
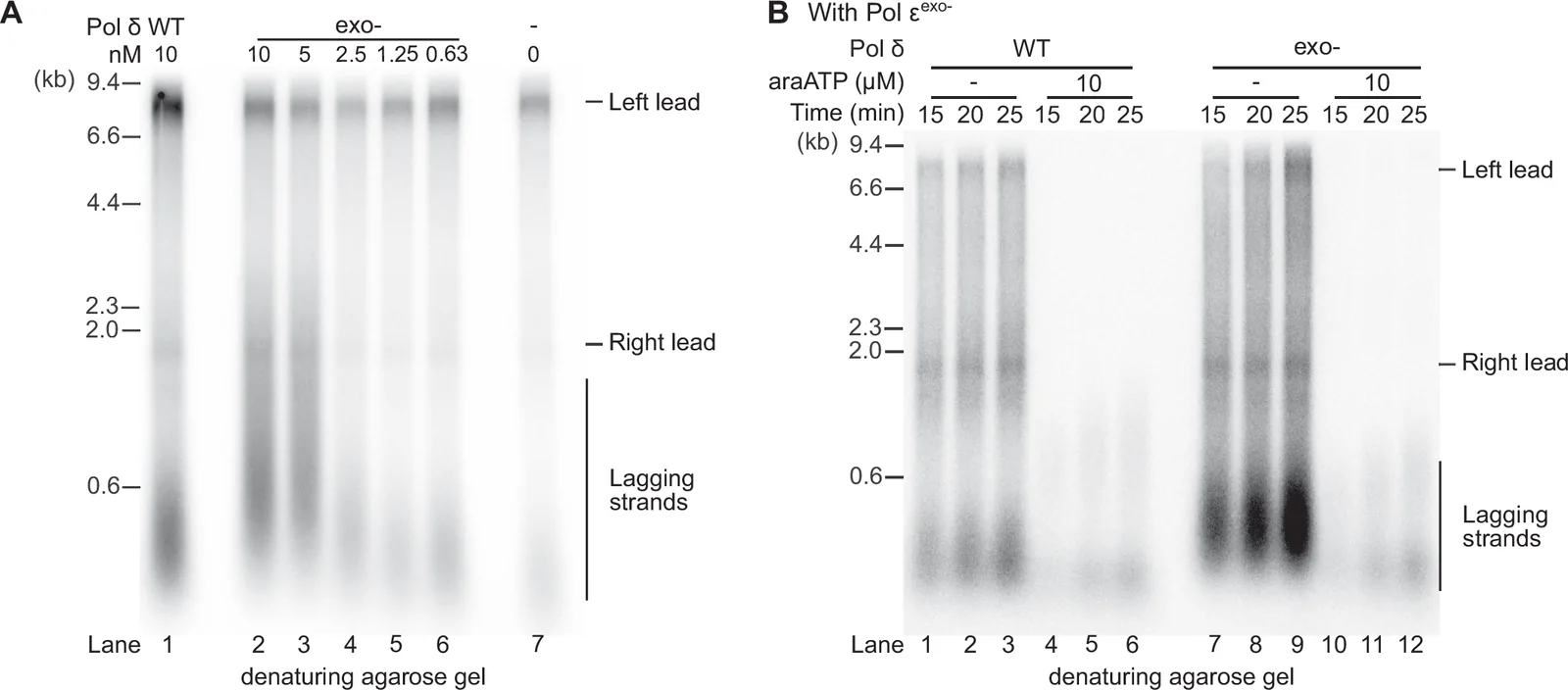
Pol δ^exo-^ strand-displacement activity is suppressed at lower concentrations. Related to Fig. 3. (**A**) Denaturing gel autoradiography of replication reactions performed on AhdI-linearised version dsDNA template as in Fig. 1E with indicated concentrations of Pol δ^exo-^. (**B**) Denaturing gel autoradiography of replication reactions performed as in Fig. 3B, but using fourfold less (2.5 nM) Pol δ^exo-^. Source data are available online for this figure.

Previous studies in budding yeast have shown that Pol δ corrects mismatches on the leading strand generated by Pol ε (Zhou and Kunkel, 2022 and references therein). Given this, and that our in vitro data indicates that Pol δ carries out leading-strand synthesis in the presence of 10 μM ara nucleotides (Fig. 2), we next wondered whether Pol δ removes ara nucleotides incorporated into the nascent leading strand. To test this, we expressed and purified a proofreading-defective mutant of Pol δ (Pol δ^exo-^, D321A, E323A) and performed replication reactions using either this or Pol δ with either Pol ε or Pol ε^exo-^ (Fig. 3A,B). Pol δ^exo-^ mutants have been previously shown to exhibit high levels of strand-displacement activity in vitro (Jin et al, 2003). Consistent with this, we observed significant lengthening of lagging strands with Pol δ^exo-^ relative to Pol δ (Fig. 3A–C, compare lanes 1–3 with 7–9) which was suppressed by lowering Pol δ^exo-^ concentration (Fig. EV3A,B). Upon 10 μM araATP addition, this strand-displacement activity was largely abolished, and leading-strand synthesis was attenuated relative to reactions with wild-type Pol δ (Fig. 3A,B, compare lanes 4–6 with 10–12). Together with Fig. 2, this suggests that Pol δ synthesises and proofreads leading strands in the presence of 10 μM araATP with either Pol ε or Pol ε^exo-^. To further test whether these nascent products were made by Pol δ, we repeated the experiment with a truncated form of Pol ε (Pol ε^Δcat^) that lacks the catalytic domain of the Pol2 subunit, but can still support CMG assembly (Yeeles et al, 2017). This revealed similar product lengths compared to experiments using either Pol ε or Pol ε^exo-^, indicating the products are synthesised by Pol δ (compare Fig. 3A, B, lanes 4–6 and 10–12 with Fig. 3C, lanes 4–6 and 10–12), further confirming that Pol δ takes over leading-strand synthesis in the presence of 10 μM araATP.

### A single template-embedded nucleoside analogue does not significantly block leading-strand synthesis

**Figure EV4:**
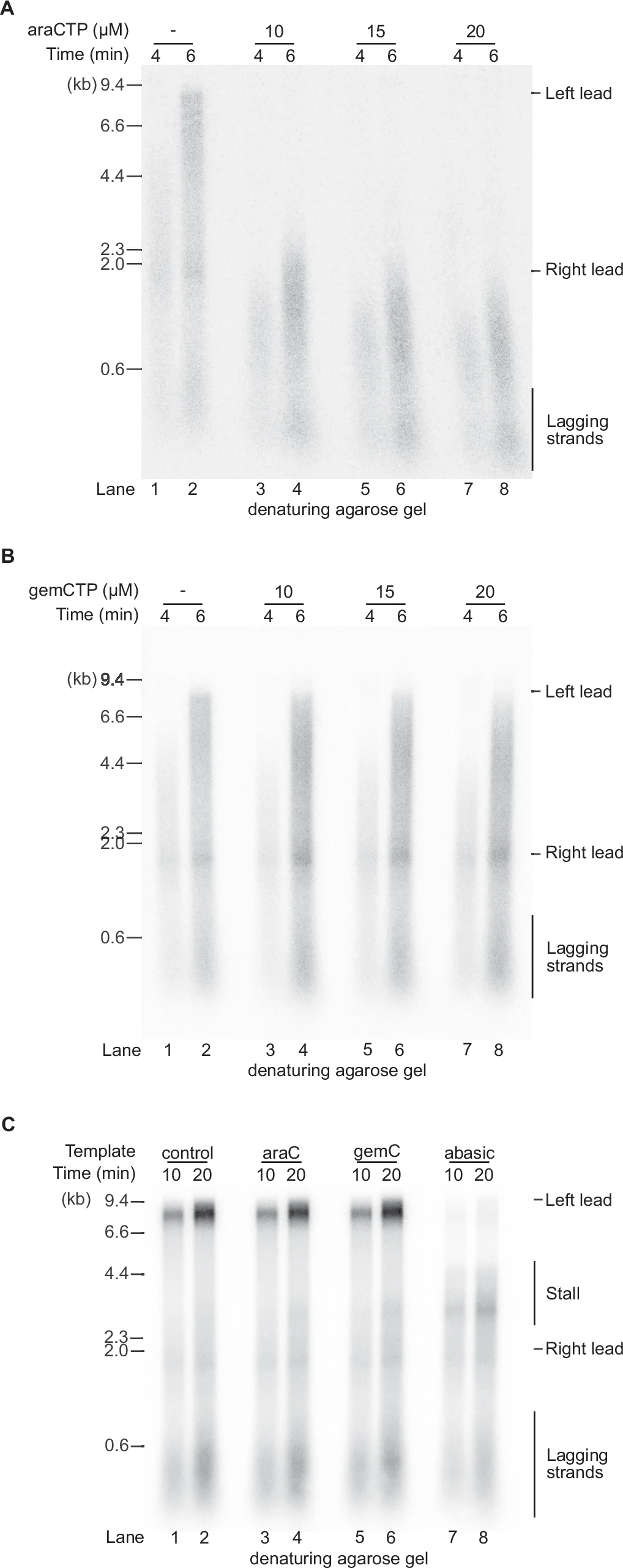
Effects of araC and gemC on DNA synthesis when present in solution as triphosphates or embedded in the leading-strand template. Related to Fig. 4. (**A**, **B**) Denaturing gel autoradiography of replication reactions with indicated concentrations of either araCTP (**A**) or gemCTP (**B**) performed on AhdI-linearised version dsDNA template as in Fig. 1E except α- ^32^P dTTP radiolabel was used. (**C**) Denaturing gel autoradiography of replication reactions performed as in Fig. 4B, but without post-quench SmaI treatment. Source data are available online for this figure.

**Figure 4:**
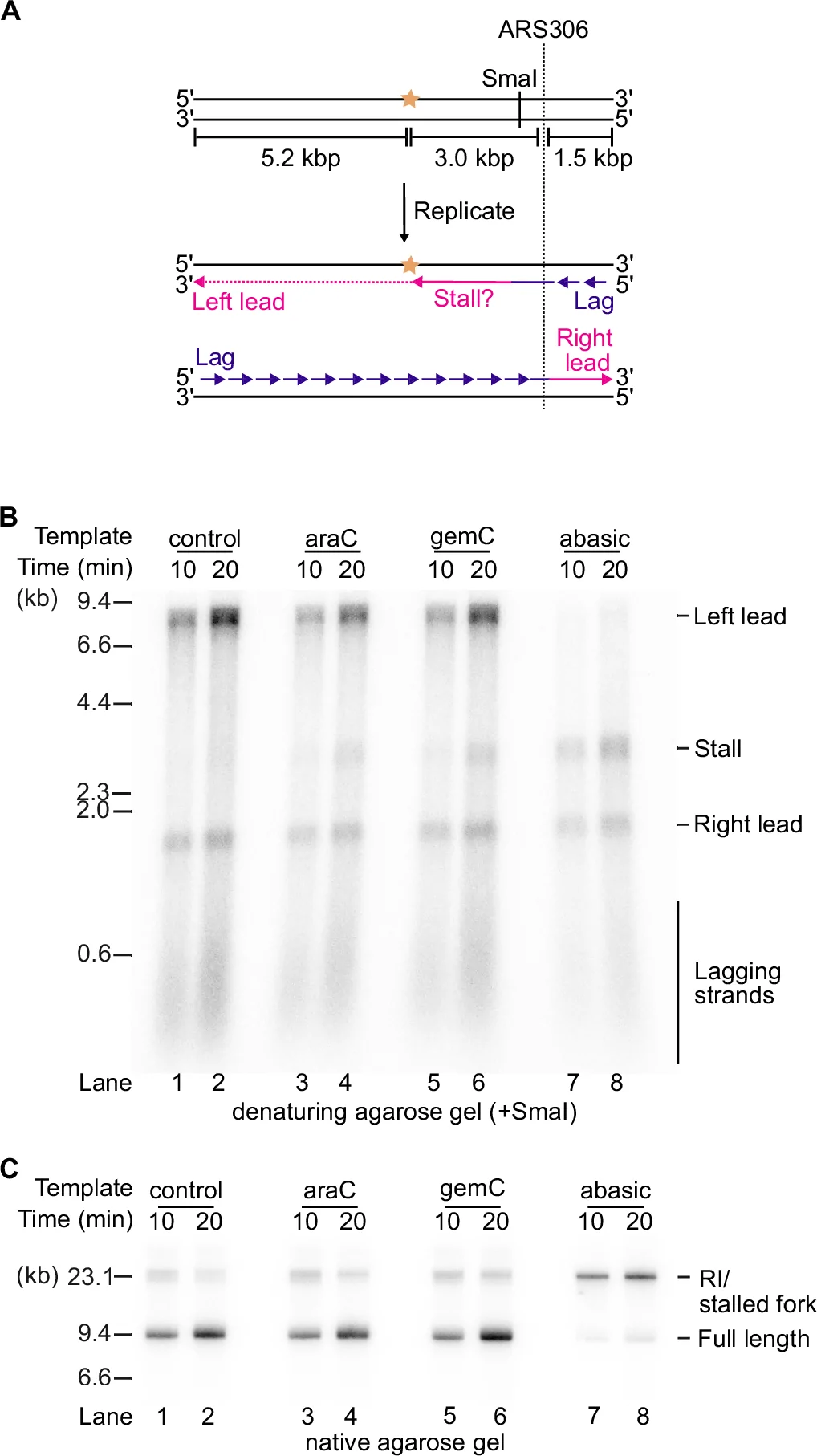
A single template-embedded nucleoside analogue has little effect on leading-strand synthesis. (**A**) Schematic representation of an in vitro replication reaction on AhdI-linearised templates modified to contain either a deoxycytidine (control), araC, gemC, or an artificial abasic site approximately 3 kbp to the left of ARS306 in the leading-strand template (gold star) (see Appendix Fig. S1 and “Methods” for construction details). Predicted nascent products are shown. (**B**) Denaturing gel autoradiography of replication reactions performed on modified AhdI-linearised dsDNA templates. Reactions were treated post-quench with the restriction endonuclease SmaI (see “Methods”). See Appendix Fig. S5 for lane quantification. (**C**) Native gel autoradiography of replication reactions performed on modified AhdI-linearised dsDNA templates. Source data are available online for this figure.

As observed for araATP, araCTP also inhibited DNA replication catalysed by the replisome in a dose-dependent manner (Fig. EV4A) whereas addition of up to 20 μM gemCTP had a negligible effect on leading-strand synthesis (Fig. EV4B). This is consistent with gemC having a weaker affinity for polymerase active sites relative to ara analogues and highlights the importance of the arabinose sugar in inhibiting DNA synthesis (Huang et al, 1991; Richardson et al, 2004; Holzer et al, 2019). Despite these differences, both araC and gemC are detectable in genomic (g)DNA suggesting that subpopulations of each analogue can escape proofreading during synthesis and be incorporated into nascent strands via their 3’-hydroxyl groups (Fig. 1B) (Graham and Whitmore, 1970; Zahn et al, 1972; Boeckemeier et al, 2020; Ubhi et al, 2024). In this scenario, the incorporated analogues would be encountered by the replisome as part of the template strand during the subsequent round of replication. To test the relative effects of template-embedded arabinose (araC) and difluororibose (gemC) sugar moieties on leading-strand synthesis catalysed by the replisome, we carried out replication reactions using modified DNA templates containing either a single araC or gemC at a specific site (~3 kbp left of ARS306) on the leading-strand template (Fig. 4A). These were performed alongside reactions using templates containing either an artificial abasic site (known to potently stall leading-strand synthesis, Taylor and Yeeles, 2019) or a deoxycytidine (control) at the equivalent position, constructed in the same way (Appendix Fig. S1). After quenching, these reactions were treated with the restriction endonuclease SmaI that cuts close to the left of ARS306 (Fig. 4A) to eliminate product length heterogeneity caused by variability in the precise location of leading-strand initiation (compare Fig. 4B with Fig. EV4C) (Taylor and Yeeles, 2018). In contrast to the potent stalling caused by the abasic lesion, the presence of a single araC or gemC gave rise to a weak stall band, and full length left leading strands were still generated (Fig. 4B). Consistent with this, the profile of araC and gemC template reactions on native gels were more similar to control template reactions rather than those performed using the abasic site template, with the majority of species corresponding to full length products rather than replication intermediates (RI) or stalled forks (Fig. 4C). From this, we conclude that the presence of a single analogue in the template strand does not significantly block leading-strand synthesis, although we cannot rule out the possibility that multiple clustered embedded analogues may prove a greater impediment.

### Pol δ proofreading promotes resistance against ara nucleoside analogues in vivo

We next wanted to define how the replisome responds to nucleoside analogues in vivo. The proofreading activities of both Pol ε and Pol δ have been previously implicated in modulating cell sensitivity to araC (Tsuda et al, 2017; Nishizawa et al, 2025). We hypothesised that if Pol δ synthesises and proofreads both the lagging- and leading-strands in vivo in the presence of ara nucleosides, as is predicted from our in vitro data (Figs. 2 and 3), then cells expressing an exonuclease deficient form of Pol δ should be exquisitely sensitive to these drugs. Owing to difficulties in generating homozygous Pol δ proofreading-deficient mammalian cell lines (Nishizawa et al, 2025), we decided to explore this hypothesis using haploid budding yeast strains.

**Figure EV5:**
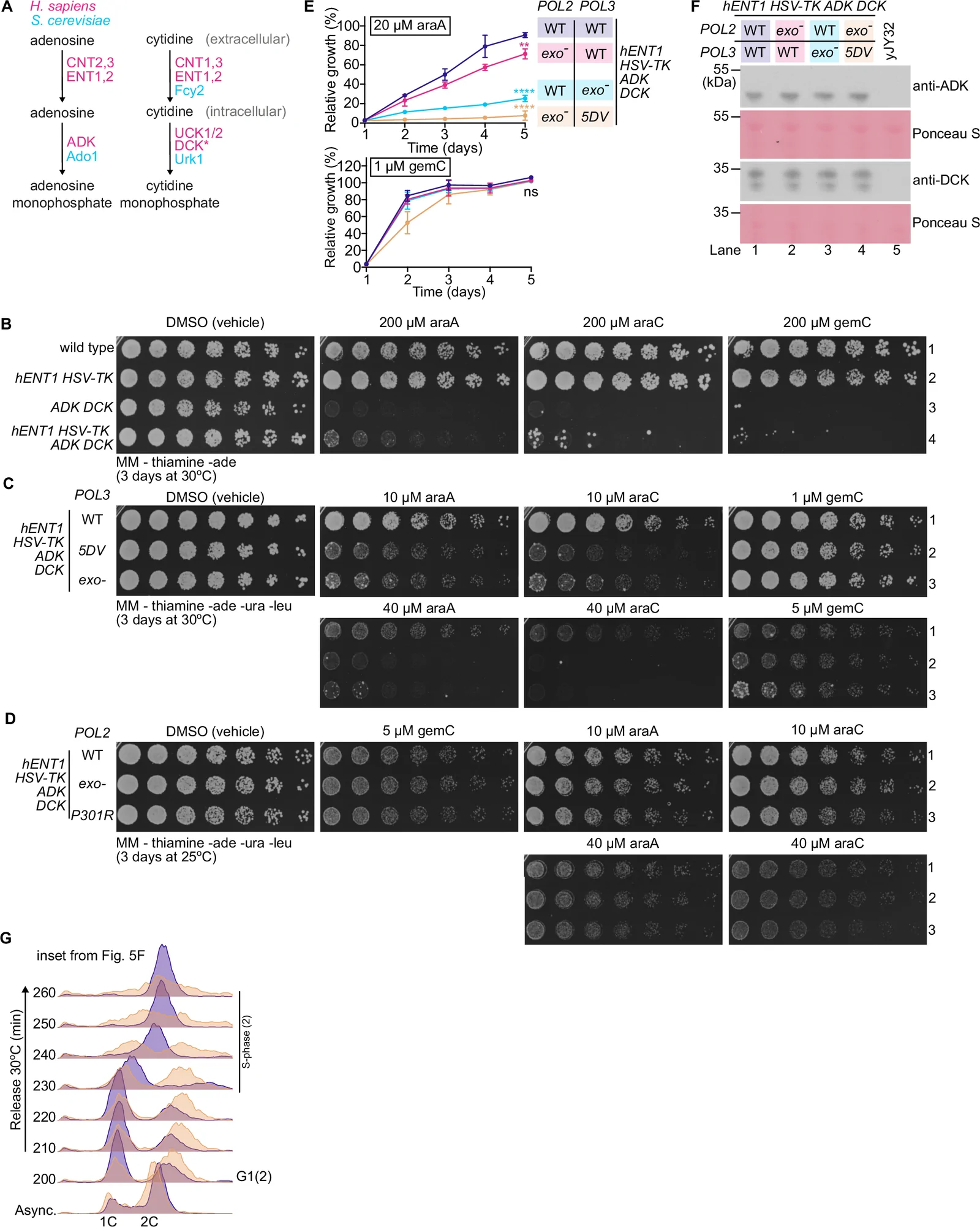
The role of proofreading in tolerating ara nucleosides in vivo. Related to Fig. 5. (**A**) Schematic illustrating the human (pink) and yeast (blue) transporters and kinases involved in uptake and activation of adenosine and cytidine, respectively. *Whilst cytidine is not a preferred substrate for DCK, this kinase phosphorylates cytidine analogues. (**B**) Threefold serial dilutions of the indicated genotypes (yHO38, yPZ4587, yHO36, yHO25) spotted onto media with the indicated conditions. (**C**) Serial dilutions as in (**B**) of the indicated genotypes (yHO56, yHO66, yHO50). (**D**) Serial dilutions as in (**B**) of the indicated genotypes (yHO45, yHO43, yHO60). (**E**) Graphs quantifying growth over 5 days of strains as in Fig. 5A for indicated condition. Error bars represent standard deviation of three biological replicates. The measure of centre of the error bars represents the mean. Significance of differences relative to wild-type at 5-day timepoint was estimated by one-way ANOVA with Tukey’s test for multiple comparisons, as in Fig. 5B. *P* values, additional comparisons and test summaries are shown in Appendix Table S4. (**F**) Western blots from strains in Fig. 5A alongside yJY32 (control) against ADK and DCK. (**G**) Zoomed-in view of flow cytometry profile presented in Fig. 5F. Source data are available online for this figure.

Wild-type budding yeast is insensitive to ara nucleosides owing to low expression of nucleoside salvage kinases required for the rate-limiting step of their activation (Jordheim et al, 2013, Fig. EV5A). We therefore engineered budding yeast cells to constitutively express a human nucleoside transporter (hENT1), along with human adenosine and deoxycytidine kinases (ADK and DCK, respectively) and Herpes Simplex Virus thymidine kinase (HSV-TK) to facilitate uptake and activation of ara nucleosides, respectively. Expression of ADK and DCK alone was sufficient to sensitise cells to adenosine and cytidine analogues (Fig. EV5B).

**Figure 5:**
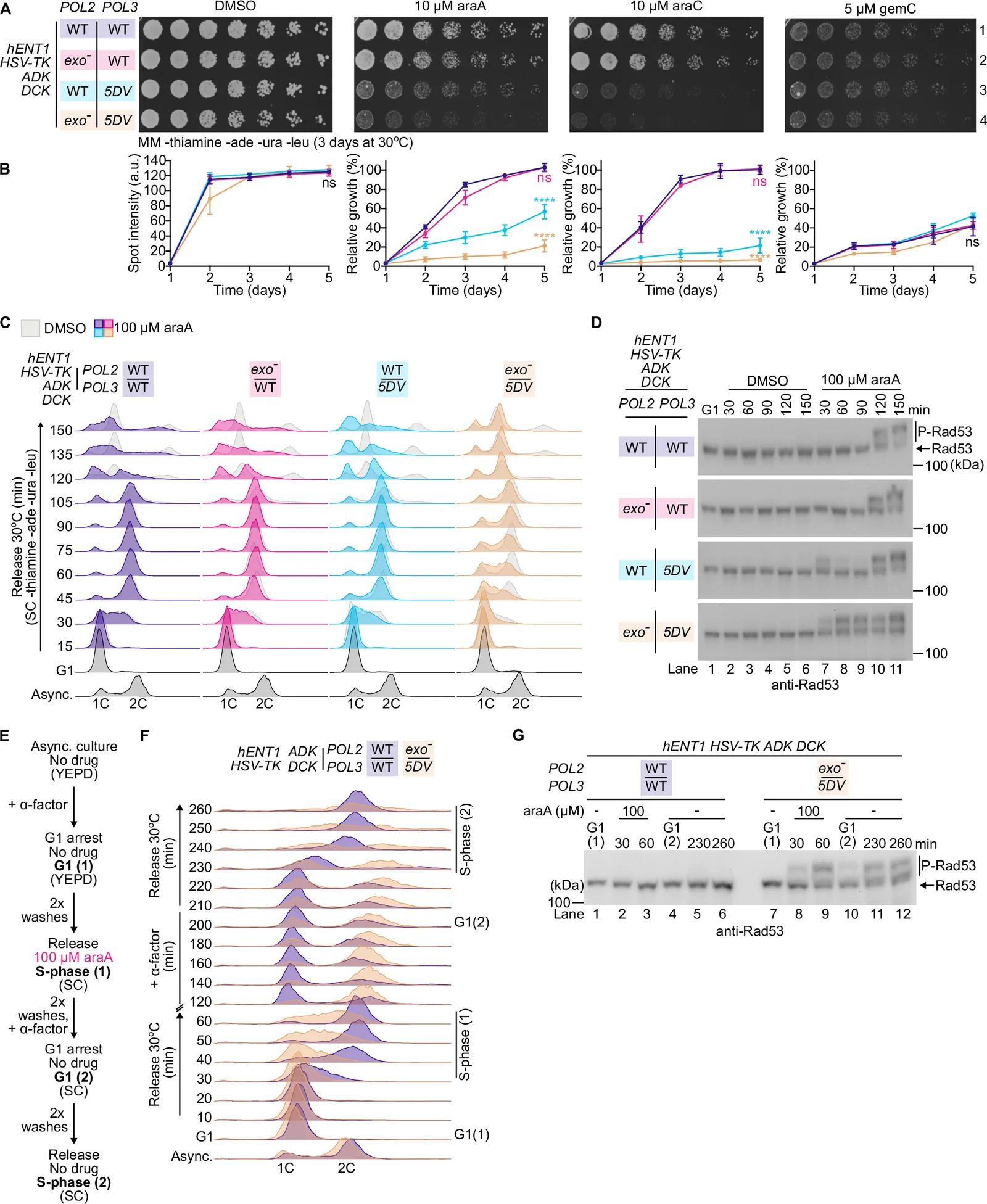
The proofreading activity of Pol δ is critical for cell survival against ara nucleosides. (**A**) Threefold serial dilutions of the indicated genotypes (yHO64, yHO43, yHO66, and yHO62) spotted onto media with the indicated conditions. MM -thiamine -ade -ura -leu, minimal medium without thiamine, adenine, uracil, and leucine. (**B**) Graphs quantifying growth over 5 days for each condition, as described in Methods and Appendix Fig. S3. Error bars represent standard deviation of three biological replicates. The measure of centre of the error bars represents the mean. Significance of differences relative to wild-type at 5-day timepoint was estimated by one-way ANOVA with Tukey’s test for multiple comparisons. Asterisks indicate *P ≤ 0.05, **P ≤ 0.01, ***P ≤ 0.001, ****P ≤ 0.0001. ns, not significant. *P* values, additional comparisons and test summaries are shown in Appendix Table S4. (**C**) DNA content of G1-arrested yeast strains in (**A**) analysed by flow cytometry following release into media containing either DMSO (grey) or 100 μM araA (colours). SC -thiamine -ade -ura -leu, synthetic complete medium without thiamine, adenine, uracil, and leucine (SC). (**D**) Western blot from experiment in (**C**) against Rad53. (**E**) Reaction scheme involving *POL2 POL3* (yHO64) and *pol2 exo- pol3-5DV* (yHO62) strains. (**F**) DNA content of indicated strains from experiment outlined in (**E**) analysed by flow cytometry. (**G**) Western blot from experiment in (**F**) against Rad53. Source data are available online for this figure.

Using this background, we made strains harbouring previously characterised inactivating mutations in the exonuclease domains of Pol ε and/ or Pol δ. Yeast strains carrying alanine substitutions in catalytic residues of either Pol ε (*pol2-D290A, E292A*, hereafter referred to as *pol2 exo-*) or Pol δ (*pol3-D321A, E323A*, hereafter referred to as *pol3 exo-*) are viable (Morrison et al, 1991, 1993) but result in synthetic lethality when combined in haploids (Morrison and Sugino, 1994). However, haploid *pol2 exo-* strains are viable with the *pol3-D520V* allele (hereafter referred to as *pol3-5DV*) which retains slightly more exonuclease activity compared to the canonical *pol3 exo-* mutant, but much less than the wild-type enzyme (Zhou et al, 2021; Jin et al, 2001). Loss of Pol δ proofreading activity alone (*pol3-5DV* or *pol3 exo-*) strongly sensitised cells to 10 μM araA or araC (Fig. 5A, compare rows 1 and 3; Figs. 5B and EV5C). In contrast, loss of Pol ε proofreading activity alone (*pol2 exo-* or *pol2-P301R*) had no effect on viability (Fig. 5A compare rows 1 and 2; Figs. 5B and EV5D) although began to sensitise cells at higher concentrations (20 μM, EV5E, upper). However, loss of Pol ε proofreading did lead to enhanced sensitivity towards ara nucleosides when Pol δ was unable to proofread (*pol2 exo- pol3-5DV*) (Fig. 5A, compare rows 1/2 with 3/4; Fig. 5B). This sensitivity pattern was specific to ara nucleosides because all strains were equally sensitive to gemC (Figs. 5A,B and EV5E, lower). Importantly, differences in sensitivity were not due to differential expression of nucleoside kinases ADK and DCK (Fig. EV5F).

From this, we conclude that loss of Pol δ proofreading alone markedly sensitises cells to ara nucleosides, whereas loss of Pol ε proofreading only sensitises cells when Pol δ cannot proofread.

### Pol δ proofreading is critical for timely S-phase progression when ara nucleosides are present

To explore how ara nucleosides impact S-phase progression, we released G1-synchronised cultures of these four strains into S-phase in the presence or absence of 100 μM araA and analysed their progression across two successive cell cycles by flow cytometry. In parallel, we assessed the intra-S checkpoint response by monitoring activation of the checkpoint kinase Rad53, determined by the accumulation of its phospho-shifted forms (P-Rad53) by western blot. Without drug (DMSO), strains exhibited comparable progression kinetics apart from the *pol2 exo- pol3-5DV* double mutant which showed a minor delay (Fig. 5C, pale grey profiles). When araA was present, wild-type and *pol2 exo-* cells exhibited no difference in progression through the first S-phase (S-phase (1)) relative to DMSO-treated cells but were delayed during the second S-phase (S-phase (2)) (Fig. 5C, compare 15–60 min with 120–150 min). This S-phase (2) defect is likely due to a rapid increase in cellular concentration of araA upon cell division in araA-containing media, rather than a response to template-embedded analogues (see later). As expected from Fig. 5A, in the presence of araA *pol3-5DV* cells progressed slower through S-phase (1) and *pol2 exo- pol3-5DV* cells exhibited an even greater defect, with cells unable to enter S-phase (2) even after 150 min (Fig. 5C). Rad53 activation was not observed during S-phase (1) for either wild-type or *pol2 exo-* cells but was detected at low levels in an araA-dependent manner for *pol3-5DV* cells before decreasing as cells completed S-phase (1) (Fig. 5D, lanes 7–9), in line with DNA damage caused by ara nucleosides being primarily S-phase-dependent (Ewald et al, 2007). This was then followed by robust Rad53 activation as cells entered S-phase (2), consistent with slow progression for these strains after 120 min in the presence of araA (Fig. 5C,D, lanes 10–11). In contrast, we observed constitutive araA-dependent Rad53 activation for *pol2 exo- pol3-5DV* cells after release from G1, consistent with an inability to fully progress beyond S-phase (1) by 150 min (Fig. 5C,D).

These experiments indicated that cells lacking proofreading ability (*pol2 exo- pol3-5DV*) struggle to complete a single S-phase in the presence of araA within 150 min. We hence wondered whether these cells could progress into S-phase (2) given more time. To test this, we released either wild-type or *pol2 exo- pol3-5DV* cells from G1 (1) into S-phase (1) in the presence of araA. Drug was then removed by washing and cells were blocked with α-factor before being released into S-phase (2) in the absence of araA (Fig. 5E). Wild-type cells progressed efficiently through both S-phases (Fig. 5F) with no Rad53 activation in either S-phase (Fig. 5G, lanes 1–6). This is consistent with Rad53 activation in S-phase (2) being attributed to high cellular araA concentrations (Fig. 5D). As expected, *pol2 exo- pol3-5DV* cells progressed much slower through S-phase (1) relative to wild-type cells and this was accompanied by checkpoint activation (Fig. 5F,G, lanes 7–12). Strikingly, the majority of *pol2 exo- pol3-5DV* cells were still unable to enter G1 (2) after 200 min, despite having been without drug for 140 min (Fig. 5F). After removing α-factor by washing, the *pol2 exo- pol3-5DV* flow cytometry profile collapsed relative to the wild-type strain, suggesting most cells were unable to enter S-phase (2) (Fig. 5F). However, a closer inspection revealed that some cells were still able to progress (Fig. EV5G) albeit with slow kinetics, consistent with the observed Rad53 activation (Fig. 5G, lanes 10–12).

Together, these results show that proofreading of ara nucleosides by both Pol ε and Pol δ contribute to timely S-phase progression. The fact that cells lacking both activities struggle to complete a single S-phase in the presence of araA implies removal of ara nucleotides from the nascent strand is the primary resistance mechanism towards these drugs (see Discussion).

### AraA promotes a Pol ε to Pol δ switch on the leading strand in vivo

**Figure EV6:**
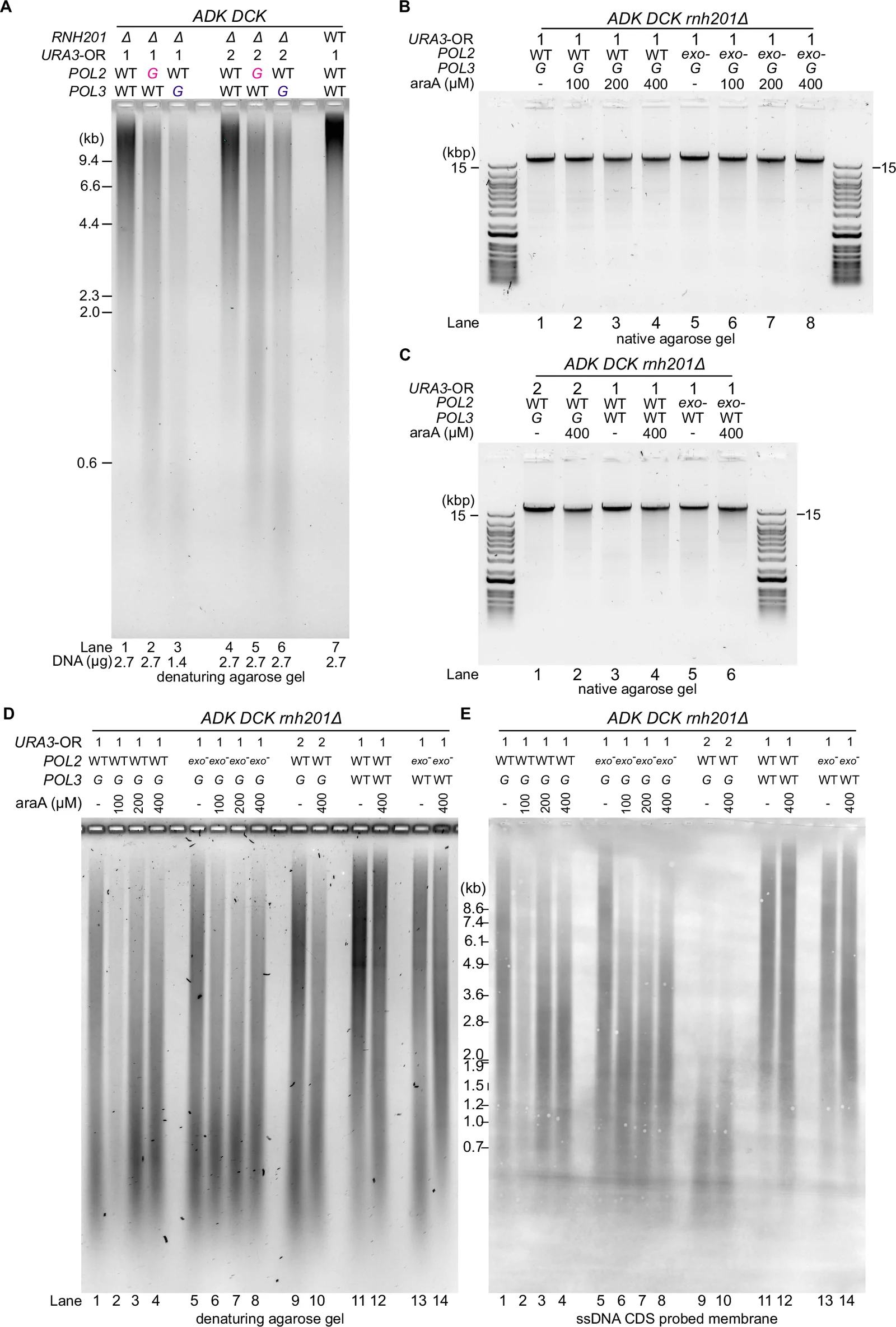
Additional evidence for greater Pol δ utility on the leading strand in vivo in response to araA. Related to Fig. 6. (**A**) Independent replicate of gel presented in Fig. 6B with addition of strain yHO105 (lane 7). Amount of DNA loaded is indicated under each lane. (**B**, **C**) Native gel analysis of gDNA from indicated strains (yHO142, yHO147, yHO144, yHO133, yHO148) treated with either DMSO (−) or araA. (**D**) Alkali-treated gDNA samples from (**B**, **C**) analysed by denaturing agarose gel electrophoresis and stained with ethidium bromide as in (**A**). (**E**) Southern blot of denaturing gel in (**D**) probed as in Fig. 6C. Source data are available online for this figure.

**Figure 6:**
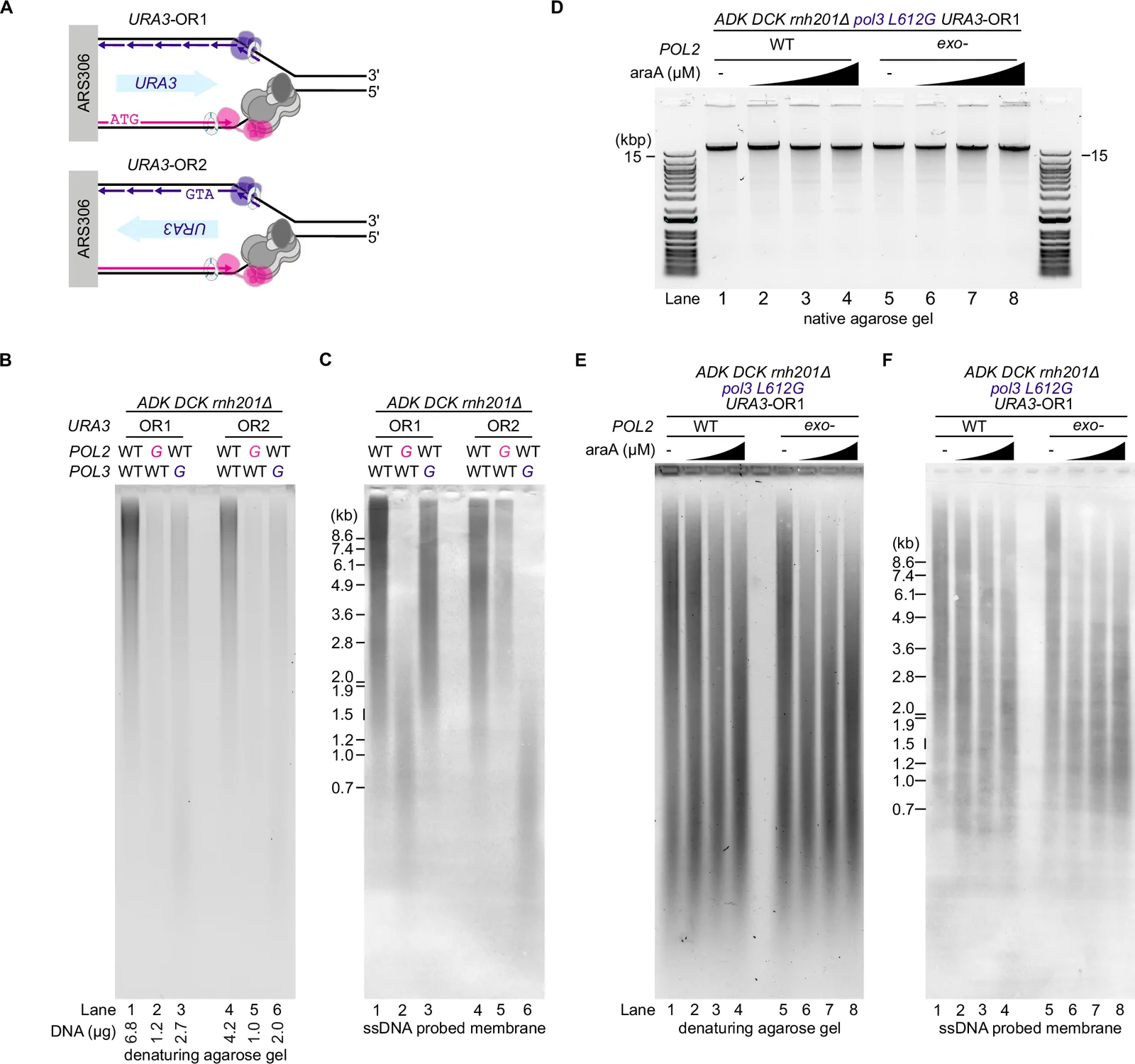
AraA promotes greater Pol δ usage on the leading strand in vivo. (**A**) Schematic illustrating the two orientations of the *URA3* cassette integrated ~849 bp to the right of ARS306 on chromosome III. The coding sequence (CDS) of the *URA3* gene is indicated by the ATG start codon. (**B**) Alkali-treated genomic DNA (gDNA) from the indicated strains (yHO134, yHO136, yHO142, yHO137, yHO140, yHO145) analysed by denaturing agarose gel electrophoresis and stained with ethidium bromide. Amount of DNA loaded is indicated under each lane. (**C**) Southern blot of denaturing gel in (**B**) using a single-stranded DNA (ssDNA) probe that hybridises to the strand containing the *URA3* gene CDS (see “Methods”). See Appendix Fig. S5 for lane quantification. (**D**) Native gel analysis of gDNA from indicated strains (yHO142 and yHO147) treated with either DMSO (−) or araA (either 100, 200 or 400 μM). 200 ng DNA was loaded per sample. (**E**) Alkali-treated gDNA samples from (**D**) analysed by denaturing agarose gel electrophoresis and stained with ethidium bromide as in (**B**). (**F**) Southern blot of denaturing gel in (**E**) probed as in (**C**). See Appendix Fig. S5 for lane quantification. Source data are available online for this figure.

The observation that loss of Pol ε proofreading does not sensitise cells to ara nucleosides when Pol δ can proofread - but does when Pol δ is unable to proofread (Fig. 5) - suggests that Pol δ contributes to leading-strand proofreading in vivo to remove ara nucleotides, consistent with our in vitro experiments (Figs. 2 and 3). To explore whether such a polymerase switch takes place in response to ara nucleosides in vivo, we took advantage of previously developed experimental approaches used to assign polymerase usage to either the leading- or lagging-strands (Pursell et al, 2007; McElhinny et al, 2010; Miyabe et al, 2011; Clausen et al, 2015; Keszthelyi et al, 2015; Miyabe et al, 2015). We generated nucleoside kinase- expressing yeast strains containing wild-type or previously characterised variants of either Pol ε or Pol δ that are promiscuous for ribonucleotide incorporation (*pol2-M644G*, and *pol3-L612G*, respectively) (Pursell et al, 2007; Clausen et al, 2015; Williams et al, 2015). DNA replicated by these polymerase variants contains elevated levels of ribonucleoside monophosphates that are not removed in the absence of a functional ribonucleotide excision repair pathway (*rnh201Δ*). As such, gDNA isolated from strains expressing these polymerase variants is sensitive to fragmentation upon alkali treatment and hence migrates quicker through denaturing agarose gels (McElhinny et al, 2010; Clausen et al, 2015) (Fig. EV6A). Using these strains, we integrated a *URA3* cassette in one of two orientations (OR1 and OR2, Fig. 6A) < 1 kbp to the right of the ARS306 replication origin which fires with approximately 90% efficiency (Poloumienko et al, 2001). Importantly, because the next nearest origin (ARS307, which has a comparable firing efficiency) is >32 kbp away, the *URA3* cassette can be assumed to be replicated by replication forks emanating from ARS306 (Pursell et al, 2007). When in OR1, the ATG-containing coding strand of *URA3* will therefore be synthesised as the nascent leading strand by Pol ε (Fig. 6A). Consistent with this, Southern blot analysis using a single-stranded probe against the coding strand revealed that this strand is alkali-sensitive from *pol2-M644G* cells, but alkali-insensitive from *pol3-L612G* cells (Fig. 6B,C, lanes 1–3). The converse is true when the orientation of *URA3* was inverted (OR2, Fig. 6B,C, lanes 4–6).

We reasoned that if ara nucleosides promote a Pol ε to Pol δ switch on the leading strand in vivo, then gDNA isolated from *pol3-L612G URA3*-OR1 cells should become more sensitive to alkali with increasing dose of ara nucleosides. Moreover, the Southern blot profile should more closely resemble that of *pol3-L612G URA3*-OR2 cells (Fig. 6C, compare lanes 3 and 6). To test this, we extracted gDNA from asynchronous cultures of *pol3-L612G URA3*-OR1 cells incubated with increasing concentrations of araA for 30 h. We performed this experiment alongside an isogenic strain additionally carrying the *pol2 exo-* allele since our in vitro and genetics data indicates a polymerase switch would be favoured under these conditions (Figs. 1, 2, and 5). Analysis by native agarose gel electrophoresis revealed a single prominent band for each sample, corresponding to high molecular weight gDNA (Fig. 6D). Following alkali treatment, migration of control samples through a denaturing agarose gel revealed two main populations, with slowly migrating species likely corresponding to gDNA replicated by either Pol ε or Pol ε^exo-^ and faster migrating, alkali-sensitive species likely corresponding to strands synthesised by Pol δ^L612G^ (Fig. 6E, lanes 1 and 5). We noticed that upon treatment with araA, this bimodal distribution became less defined, and this was most evident for the *pol2 exo-* strain at higher drug concentrations (Fig. 6E, compare lanes 7–8 with lane 5). We next specifically visualised nascent leading strands using the single-stranded probe described above. This revealed an increase in gel migration for both *POL2* and *pol2 exo-* strains with increasing araA dose, with the shift occurring at lower araA concentrations for the *pol2 exo-* strain (Fig. 6F). This observed loss of signal intensity at the top of the gel was reproducible across independent experiments (Fig. EV6B–E) and is dependent on the *pol3-L612G* allele because araA-treated *POL3* cells did not exhibit a comparable shift (compare Fig. 6F lanes 4/8 with Fig. EV6E lanes 12/14). Taken together, these data suggest increased but not constitutive Pol δ usage on the leading strand upon addition of araA in vivo, and that this polymerase switch is promoted by loss of Pol ε proofreading.

## Discussion

Using a reconstituted DNA replication system and budding yeast genetics, we show that araATP induces a Pol ε to Pol δ switch on the leading strand in vitro (Fig. 2) and in vivo (Fig. 6) in a manner that is shaped by both drug dose and Pol ε proofreading capability. This behaviour is consistent with our previous studies demonstrating that Pol δ acts to recouple leading-strand synthesis with CMGε after encountering various site-specific template lesions (Guilliam and Yeeles, 2020, 2021), and with earlier work showing that Pol δ carries out the bulk of leading-strand synthesis in select DNA repair pathways in vivo (Miyabe et al, 2015; Donnianni et al, 2019). It also fits with the notion that Pol δ can correct mismatches for Pol ε on the nascent leading strand (Bulock et al, 2020; Zhou et al, 2021).

### Ara chemotherapeutics and leading-strand synthesis

**Figure 7:**
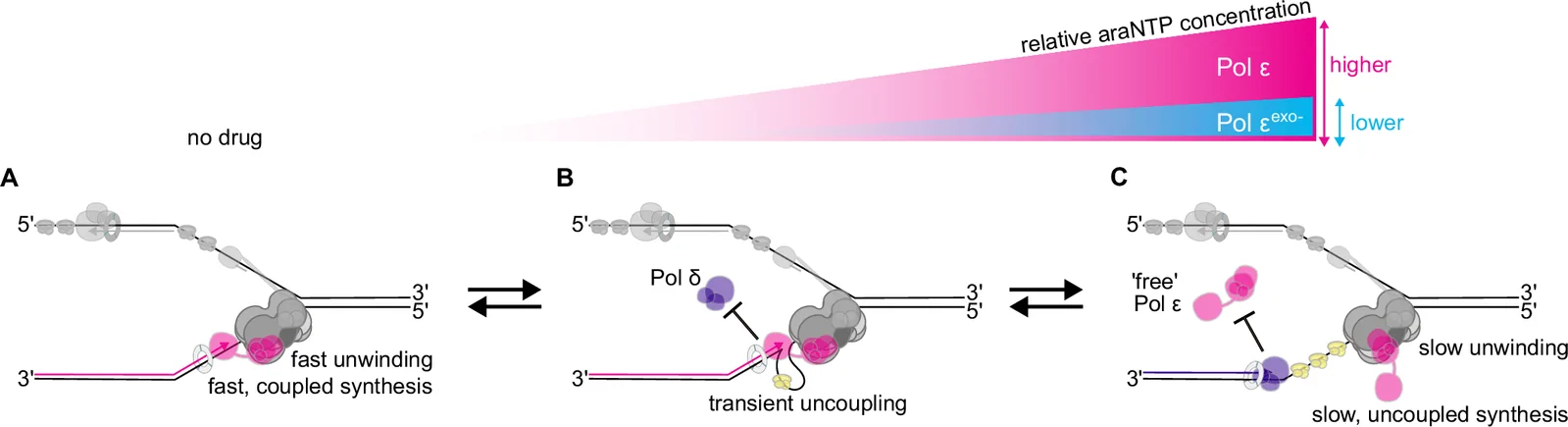
The effects of ara nucleoside chemotherapeutics on leading-strand synthesis. (**A**) In the absence of araNTPs, Pol ε-catalysed leading-strand replication is coupled to CMG helicase unwinding resulting in fast and efficient synthesis rates. (**B**) The extent of polymerase-helicase uncoupling following infrequent stalling events at low concentrations of araNTPs is minimised by the proofreading activity of Pol ε. As such, Pol ε remains associated with the 3’ end of the nascent leading strand preventing Pol δ access. (**C**) At higher araNTP concentrations (pink wedge), or at lower concentrations in the absence of Pol ε proofreading (Pol ε^exo-^, blue wedge), increased stalling levels cause CMG-bound Pol ε to dissociate from the 3’ end of the nascent leading strand as CMG continues to slowly unwind dsDNA. Owing to its high affinity for PCNA-primed 3’ ends, Pol δ outcompetes ‘free’ Pol ε and takes over leading-strand synthesis. The extent of helicase-polymerase uncoupling under these conditions is shaped by both the drug dose, and ability of Pol δ to proofread incorporated ara nucleosides.

Under normal conditions, nascent leading-strand synthesis catalysed by CMGε is established following Pol δ-mediated extension of the first Okazaki fragment deposited by Pol α across the origin (Aria and Yeeles, 2019). This coupled leading-strand synthesis stimulates CMG unwinding resulting in maximal synthesis rates (Yeeles et al, 2017; Fig. 7A). The estimated processivity of PCNA-bound Pol ε on an RPA-coated singly primed ssDNA template is ~500 nt (Chilkova et al, 2007). Therefore, it is likely that Pol ε frequently dissociates from the 3’ end of the nascent strand but, owing to its multitude of contacts with the CMG helicase (Langston et al, 2014; Georgescu et al, 2014; Goswami et al, 2018), rapidly rebinds and resumes efficient synthesis.

In the presence of low levels of ara nucleotides, synthesis by CMGε stalls owing to the arabinose sugar being difficult to extend from (Tsuda et al, 2017; Huang et al, 1990; Holzer et al, 2019). Since dNTPs are in large molar excess, the frequency of stalling would be low. Nevertheless, the extent of helicase-polymerase uncoupling arising from these stalling events is minimised by Pol ε proofreading activity (Fig. 1F). This likely enables CMG-bound Pol ε to remain associated with the 3’ end of the nascent leading strand, poised to resume fast, coupled synthesis following the removal of terminal ara nucleotides, and thereby prevent Pol δ access to the nascent leading strand (Fig. 7B). In line with this, loss of Pol δ has little, if any, effect on leading-strand synthesis under these conditions (Fig. 2C).

However, at higher drug concentrations we propose the increased stalling frequency causes CMG-bound Pol ε to vacate the 3’ end of the nascent leading strand. This generates an optimal substrate for Pol δ which has a higher affinity for PCNA and preferentially binds PCNA-primed ssDNA relative to ‘free’ Pol ε (Chilkova et al, 2007; Georgescu et al, 2014). As such, Pol δ takes over leading-strand synthesis in a manner that is uncoupled from DNA unwinding (Figs. 1D, 2E, and 7C) explaining why loss of Pol ε proofreading has less of an effect on synthesis rates than would be expected based on primer extension data (compare Fig. 1G with EV1B,C). Consequently, stimulation of helicase unwinding activity by Pol ε is lost (Yeeles et al, 2017) and synthesis becomes rate-limited by the requirement to excise ara nucleotides from the nascent strand. This is consistent with the rate of leading-strand synthesis catalysed by Pol δ being similar to that carried out by Pol ε in the presence of ara nucleotides (Fig. 2D), despite the former polymerase exhibiting vastly greater synthesis rates on singly primed ssDNA templates (Yeeles et al, 2017; Chilkova et al, 2007; compare Fig. EV1B with EV2A). It also fits with the observation that loss of Pol δ proofreading further reduces leading-strand synthesis rates (Fig. 3). We note that these data would also be consistent with the notion that high levels of ara nucleotides prevent the establishment of CMGε-coupled synthesis when present during initiation.

This model is additionally supported by Fig. 6, which shows that loss of Pol ε proofreading enhances the use of Pol δ on the leading strand in vivo, and may explain why *pol2 exo-* cells do not exhibit enhanced sensitivity to ara nucleosides when Pol δ can proofread (Fig. 5A). This observation that Pol ε proofreading status modulates Pol δ activity on the leading strand suggests that a Pol ε to Pol δ switch is not constitutive in vivo, although is enhanced with increasing drug dose (Fig. 6). Whilst we do not know the concentration of active (phosphorylated) drug in cells, it is possible that additional factors act to minimise uncoupling caused by ara analogues in vivo. Consistent with this hypothesis, loss of the leading-strand clamp loader CTF18-RFC sensitises cells to araC (Washif et al, 2023) and prolonged uncoupling leads to nascent strand degradation and replication fork reversal in *Xenopus* egg extracts (Kavlashvili et al, 2023). Pol δ replicates both strands in yeast strains expressing a truncated form of the Pol ε catalytic subunit (*pol2-16*) that can support CMG assembly but lacks polymerase and exonuclease activities (Kesti et al, 1999; Dua et al, 1999; Garbacz et al, 2018). These strains are profoundly sick and exhibit high spontaneous mutation rates (Garbacz et al, 2018). Therefore, mechanisms acting to reduce the extent of leading strand uncoupling may be critical to maintain cell viability in the presence of these chemotherapeutics.

### Polymerase proofreading and resistance against ara chemotherapeutics

Previous work has shown that Pol ε and Pol δ proofreading provide tolerance to araC in vivo (Tsuda et al, 2017; Nishizawa et al, 2025). We extend these findings by demonstrating that loss of Pol δ proofreading exquisitely sensitises cells to ara nucleosides (Fig. 5). Since loss of this activity results in attenuated leading-strand synthesis in vitro (Fig. 3), we suggest this phenotype can in part be explained by increased uncoupling on the leading strand. Such a scenario would result in increased levels of RPA-coated ssDNA, consistent with intra-S checkpoint activation being exacerbated in *pol3-5DV* cells (Fig. 5D). Moreover, only a small subset of cells lacking all polymerase proofreading activities can progress through later cell cycles after being transiently exposed to these drugs (Fig. 5F), albeit with an activated checkpoint (Fig. 5G). Therefore, whilst we cannot exclude the contribution of post-replicative repair pathways, our data suggest that the removal of ara nucleotides from nascent DNA by Pol ε and Pol δ acts as the initial and likely primary resistance mechanism against these drugs, consistent with the relatively small effect of a template-embedded analogue on leading-strand synthesis (Fig. 4). This could explain why genetic screens have been more successful in uncovering upstream metabolic suppressors of ara nucleosides rather than downstream pathways involved in their repair (Zhao et al, 2023).

We do not observe the same genetic relationship with the related cytidine analogue gemC since cells treated with this drug are equally sick irrespective of their proofreading status (Figs. 5B and EV5E, lower). When diphosphorylated, gemC acts as an inhibitor of ribonucleotide reductase (akin to hydroxyurea) resulting in dNTP depletion (Heinemann et al, 1990). Given that gemC is more cytotoxic than araC (Fig. 5A,B), yet its triphosphate is unable to significantly inhibit DNA synthesis in vitro (Fig. EV4B), we believe dNTP depletion is likely the dominant mode-of-action of gemC in our budding yeast model. Interestingly, a genetic screen against gemC uncovered mutations in RNase H2 as top hits that sensitise human cells to this drug (Olivieri et al, 2020). Our data suggest this may be explained by diphosphorylated gemC-mediated depletion of dNTPs. This would cause an elevated NTP/dNTP ratio leading to increased NTP incorporation into nascent DNA, necessitating RNase H2-catalysed removal via the ribonucleotide excision repair pathway (Sparks et al, 2012).

### Ara chemotherapeutics and lagging-strand synthesis

Previous studies have shown that ara nucleotides can attenuate primer synthesis and subsequent extension by Pol α in vitro (Holzer et al, 2019; Richardson et al, 2004). We noticed that lagging-strand products from in vitro replication reactions consistently appear shorter in length in the presence of ara nucleotides (e.g. Fig. 1D). However, this is also the case under conditions that favour a Pol ε to Pol δ switch on the leading strand at low concentrations of ara nucleotides (e.g. Fig. 1F lanes 10–12) that would not be expected to greatly inhibit Pol α activity (Fig. EV2B) Therefore, we suggest that the reduced length of lagging-strand products primarily stems from loss of maximum CMG-unwinding rates following a Pol ε to Pol δ switch (Yeeles et al, 2017). This results in a given number of priming events occurring over a shorter template region, hence the appearance of shorter products. Nevertheless, we suspect that ara nucleosides may also attenuate Okazaki fragment maturation in vivo giving rise to ssDNA nicks on the lagging strand. This may partly explain why *pol3-5DV* cells are sick when grown in the presence of ara nucleosides (Fig. 5A,B) and have an activated intra-S checkpoint (Fig. 5D) but appear to progress through S-phase without delay when DNA content is analysed by flow cytometry compared to wild-type cells (Fig. 5C).

Taken together, our work reveals that DNA polymerase proofreading and the plasticity of polymerase usage at the replication fork enable the replisome to tolerate ara nucleoside analogues and may have implications for how resistance arises against these and other chain-terminating nucleoside analogue chemotherapeutics in the clinic.

## Methods

**Table:** Reagents and tools table

Reagent/resource	Source	Identifier
**Antibodies**		
Anti-ADK (rabbit polyclonal)	Abcam	ab227087
Anti-DCK (rabbit polyclonal)	Abcam	ab151966; RRID:AB_3096174
Anti-Digoxigenin-AP, Fab fragments (sheep)	Roche	11093274910; RRID:AB_514497
Anti-Rabbit IgG-HRP (goat polyclonal)	Invitrogen	31462; RRID:AB_228338
Anti-Rad53 (rabbit polyclonal)	Abcam	ab104232; RRID:AB_2687603
**Experimental models**		
5-alpha Competent *E. coli* (High Efficiency)	New England Biolabs	C2987H
*S. cerevisiae* strain background W303-1A (*MATa, ade2-1; ura3-1; his3-11; trp1-1; leu2-3, 112; can1-100; rad5-535*)	Thomas and Rothstein, 1989	N/A
*S. cerevisiae*: yHO105 W303-1A *ADE2* *leu2::pTEF1-ADK-CYC1t and* *pADH1-DCK-ADH1t ::LEU2* *ura3∆::KanMX4* *URA3-OR1*	This study	N/A
*S. cerevisiae*: yHO133 W303-1A *ADE2* *ura3∆::KanMX4* *leu2::pTEF1-ADK-CYC1t and* *pADH1-DCK-ADH1t ::LEU2* *URA3-OR1* *rnh201∆::hphMX6*	This study	N/A
*S. cerevisiae*: yHO134 W303-1A *ADE2* *ura3∆::KanMX4* *leu2::pTEF1-ADK-CYC1t and* *pADH1-DCK-ADH1t ::LEU2* *URA3-OR1* *rnh201∆::hphMX6*	This study	N/A
*S. cerevisiae*: yHO136 W303-1A *ADE2* *ura3∆::KanMX4* *leu2::pTEF1-ADK-CYC1t and* *pADH1-DCK-ADH1t ::LEU2* *URA3-OR1* *rnh201∆::hphMX6* *pol2-M644G-hphNT1*	This study	N/A
*S. cerevisiae*: yHO137 W303-1A *ADE2* *ura3∆::KanMX* *leu2::pTEF1-ADK-CYC1t and* *pADH1-DCK-ADH1t ::LEU2* *URA3-OR2* *rnh201∆::hphMX6*	This study	N/A
*S. cerevisiae*: yHO140 W303-1A *ADE2* *ura3∆::KanMX4* *leu2::pTEF1-ADK-CYC1t and* *pADH1-DCK-ADH1t ::LEU2* *URA3-OR2* *rnh201∆::hphMX* *pol2-M644G-hphNT1*	This study	N/A
*S. cerevisiae*: yHO142 W303-1A *ADE2* *ura3∆::KanMX4* *leu2::pTEF1-ADK-CYC1t and* *pADH1-DCK-ADH1t ::LEU2* *URA3-OR1* *rnh201∆::hphMX6* *pol3-L612G-natNT2*	This study	N/A
*S. cerevisiae*: yHO144 W303-1A *ADE2* *ura3∆::KanMX4* *leu2::pTEF1-ADK-CYC1t and* *pADH1-DCK-ADH1t ::LEU2* *URA3-OR2* *rnh201∆::hphMX6* *pol3-L612G-natNT2*	This study	N/A
*S. cerevisiae*: yHO145 W303-1A *ADE2* *ura3∆::KanMX4* *leu2::pTEF1-ADK-CYC1t and* *pADH1-DCK-ADH1t ::LEU2* *URA3-OR2* *rnh201∆::hphMX6* *pol3-L612G-natNT2*	This study	N/A
*S. cerevisiae*: yHO147 *W303-1A* *ADE2* *ura3∆::KanMX4* *URA3-OR1* *rnh201∆::hphMX6* *leu2::pTEF1-ADK-CYC1t and* *pADH1-DCK-ADH1t ::LEU2* *pol2-D290A,E292A-hphNT1* *pol3-L612G-natNT2*	This study	N/A
*S. cerevisiae*: yHO148 *W303-1A* *ADE2* *ura3∆::KanMX4* *URA3 OR1* *rnh201∆::hphMX6* *leu2::pTEF1-ADK-CYC1t and* *pADH1-DCK-ADH1t ::LEU2* *pol2-D290A,E292A-hphNT1*	This study	N/A
*S. cerevisiae*: yHO25 W303-1A *ura3::pGAP-HSV-TK and* *pADH1-hENT1::URA3* *leu2::pTEF1-ADK-CYC1t and* *pADH1-DCK-ADH1t ::LEU2*	This study	N/A
*S. cerevisiae*: yHO36 W303-1A *ADE2* *leu2::pTEF1-ADK-CYC1t and* *pADH1-DCK-ADH1t ::LEU2*	This study	N/A
*S. cerevisiae*: yHO38 W303-1A *ADE2*	This study	N/A
*S. cerevisiae*: yHO43 W303-1A *ADE2* *ura3::pGAP-HSV-TK and* *pADH1-hENT1::URA3* *leu2::pTEF1-ADK-CYC1t and* *pADH1-DCK-ADH1t ::LEU2* *pol2-D290A,E292A-hphNT1*	This study	N/A
*S. cerevisiae*: yHO45 W303-1A *ADE2* *ura3::pGAP-HSV-TK and* *pADH1-hENT1::URA3* *leu2::pTEF1-ADK-CYC1t and* *pADH1-DCK-ADH1t ::LEU2* *POL2-hphNT1*	This study	N/A
*S. cerevisiae*: yHO50 W303-1A *ADE2* *ura3::pGAP-HSV-TK and* *pADH1-hENT1::URA3* *leu2::pTEF1-ADK-CYC1t and* *pADH1-DCK-ADH1t ::LEU2* *pol3-D321 A,E323A-natNT2*	This study	N/A
*S. cerevisiae*: yHO56 W303-1A ADE2 *ura3::pGAP-HSV-TK and* *pADH1-hENT1::URA3* *leu2::pTEF1-ADK-CYC1t and* *pADH1-DCK-ADH1t ::LEU2* *POL3-natNT2*	This study	N/A
*S. cerevisiae*: yHO60 W303-1A *ADE2* *ura3::pGAP-HSV-TK and* *pADH1-hENT1::URA3* *leu2::pTEF1-ADK-CYC1t and* *pADH1-DCK-ADH1t ::LEU2* *pol2-P301R-hphNT1*	This study	N/A
*S. cerevisiae*: yHO62 W303-1A *ADE2* *ura3::pGAP-HSV-TK and* *pADH1-hENT1::URA3* *leu2::pTEF1-ADK-CYC1t and* *pADH1-DCK-ADH1t ::LEU2* *pol2-D290A,E292A-hphNT1* *pol3-D520V-natNT2*	This study	N/A
*S. cerevisiae*: yHO64 W303-1A *ADE2* *ura3::pGAP-HSV-TK and* *pADH1-hENT1::URA3* *leu2::pTEF1-ADK-CYC1t and* *pADH1-DCK-ADH1t ::LEU2* *POL2-hphNT1* *POL3-natNT2*	This study	N/A
*S. cerevisiae*: yHO66 W303-1A *ADE2* *ura3::pGAP-HSV-TK and* *pADH1-hENT1::URA3* *leu2::pTEF1-ADK-CYC1t and* *pADH1-DCK-ADH1t ::LEU2* *pol3-D520V-natNT2*	This study	N/A
*S. cerevisiae*: yPZ4587 W303-1A *ura3::pGAP-HSV-TK and* *pADH1-hENT1::URA3*	This study	N/A
*S. cerevisiae*: yAE34 (Pol δ expression)	Yeeles et al, 2017	N/A
*S. cerevisiae*: yAE40 (Ctf4 expression)	Yeeles et al, 2015	N/A
*S. cerevisiae*: yAE41 (RFC expression)	Yeeles et al, 2017	N/A
*S. cerevisiae*: yAE42 (Top1 expression)	Yeeles et al, 2017	N/A
*S. cerevisiae*: yAE48 (Csm3/Tof1 expression)	Yeeles et al, 2017	N/A
*S. cerevisiae*: yAE88 (S-CDK truncated Clb5 *∆*1-100 expression)	Hill et al, 2020	N/A
*S. cerevisiae*: yAE95 (Pol α-primase expression)	Yeeles et al, 2015	N/A
*S. cerevisiae*: yAJ2 (Pol ε expression)	Yeeles et al, 2015	N/A
*S. cerevisiae*: yAJ25 (Pol ε^*∆*cat^ expression)	Yeeles et al, 2017	N/A
*S. cerevisiae*: yAM33 (Cdt1.Mcm2-7 expression)	Coster et al, 2014	N/A
*S. cerevisiae*: yEF4 (Ctf18-RFC expression)	(Fletcher et al, 2025)	N/A
*S. cerevisiae*: yJY106 (RPA expression)	Baretić et al, 2020	N/A
*S. cerevisiae*: yJY108 (Pol ε^exo-^ expression)	Guilliam and Yeeles, 2021	N/A
*S. cerevisiae*: yJY13 (Cdc45 expression)	Yeeles et al, 2015	N/A
*S. cerevisiae*: yJY26 (Dpb11 expression)	Yeeles et al, 2015	N/A
*S. cerevisiae*: yJY32 (Mrc1 expression)	Yeeles et al, 2017	N/A
*S. cerevisiae*: ySDK8 (DDK expression)	On et al, 2014	N/A
*S. cerevisiae*: ySDORC (ORC expression)	Frigola et al, 2013	N/A
*S. cerevisiae*: yTD6 (Sld3/7 expression)	Yeeles et al, 2015	N/A
*S. cerevisiae*: yTD8 (Sld2 expression)	Yeeles et al, 2015	N/A
*S. cerevisiae*: yVA28 (Pol δ^POL-DEAD^ expression)	Aria and Yeeles, 2019	N/A
*S. cerevisiae*: yHO33 W303-1A *bar1∆::hphNT1* *pep4∆::KanMX4* *his3::pGAL1-10 POL32-CBP/GAL4::HIS3* *ura3::pGAL1-10 pol3-D321A,E323A/POL31::URA3* (Pol δ^exo-^ expression)	This study	N/A
*S. cerevisiae*: yHO49 W303-1A *bar1∆::hphNT1* *pep4∆::KanMX4* *POL2-3XFLAG-natNT2* *ura3::pGAL1-10 DPB2/DPB3::URA3* *trp1::pGAL1-10 pol2-P301R/DPB4-CBP::TRP1* (Pol ε^P301R^ expression)	This study	N/A
**Recombinant DNA**		
bPZ1352: pUC57-Kan/ *DCK-ADH1t* *pTEF1-ADK-CYC1t*	This study	N/A
bPZ1353: pATP403red/ *pGAP-hENT1-TDH3t* *pPGK1-HSV-TK-PGK1t* *pADH1-DCK-ADH1t* *pTEF1-ADK-CYC1t*	This study	N/A
pAM3 (Cdc6 expression)	Frigola et al, 2013	N/A
pATP403red	Addgene	Gift from Jun Ishii RRID:Addgene_166027
pET28a-Mcm10 (Mcm10 expression)	Yeeles et al, 2015	N/A
pFJD5 (GINS expression)	Yeeles et al, 2015	N/A
pJM126 (RPA purification)	Addgene	49339
vHO14: pRS306/ *pol3-D321A,E323A- pGAL-1-10- POL31*	This study	N/A
vHO15: pFA6a-hphNT1/ *POL2*	This study	N/A
vHO16: pFA6a-natNT2/ *POL3*	This study	N/A
vHO17: pFA6a-hphNT1/ *pol2-D290A,E292A*	This study	N/A
vHO18: pFA6a-natNT2/ *pol3-D321A,E323A*	This study	N/A
vHO19: pFA6a-natNT2/ *pol3-D520V*	This study	N/A
vHO20: pFA6a-hphNT1/ *pol2-P301R*	This study	N/A
vHO21: pRS304/ *pol2-P301R- pGAL-1-10- DPB4-TEV-CBP*	This study	N/A
vHO23: pFA6a-hphNT1/ *pol2-M644G*	This study	N/A
vHO28: pFA6a-natNT2/ *pol3-L612G*	This study	N/A
vHO9: pRS305/ *pADH1-DCK-ADH1t* *pTEF1-ADK-CYC1t*	This study	N/A
vJY19 (PCNA purification)	Yeeles et al, 2017	N/A
vJY201: pFA6a URA3	This study	N/A
vRW31	This study	N/A
ZN3	Taylor and Yeeles, 2018	N/A
**Chemicals, enzymes, and other reagents**		
α-factor	GenScript	RP01002
[α-^32^P] dCTP	Hartmann Analytic	SCP-205
[α-^32^P] dTTP	Hartmann Analytic	SCP-206
1 Kb Plus DNA Ladder	Invitrogen	10787018
2-Mercaptoethanol	Sigma-Aldrich	M6250
Agarose	Lonza	50004
AhdI	New England Biolabs	R0584
Ammonium sulfate	Sigma-Aldrich	A5132
Ampicillin sodium	Formedium	AMP
BamHI-HF	New England Biolabs	R3136S
Bovine Serum Albumin (BSA)	Invitrogen	AM2616
Bromophenol Blue	Sigma-Aldrich	B0126
Caesium chloride	Sigma-Aldrich	C4036
Cytarabine (araC)	Cayman Chemical	16069
Cytarabine triphosphate (araCTP)	Jena Bioscience	NU-1170
Digoxygenin (DIG) Easy Hyb	Roche	11796895001
Dimethyl Sulfoxide (DMSO)	Fisher BioReagents	10103483
DL-Dithiothreitol (DTT)	Melford	D11000
DNA Molecular Weight Marker VII, DIG-labelled	Roche	11669940910
dNTP set (dATP, dCTP, dGTP, dTTP)	Invitrogen	10297018
Enhanced chemiluminescence (ECL) detection reagent	Cytiva	RPN2106
Ethanol	Honeywell	32221
Ethidium bromide (1% in water)	Sigma-Aldrich	46067
Ethylenediaminetetraacetic acid (EDTA)	Sigma-Aldrich	EDS
Ficoll-400	VWR	437092S
Gemcitabine (gemC)	Cayman Chemical	11690
Gemcitabine triphosphate (gemCTP)	Jena Bioscience	NU-1607
Glycerol	VWR	24388.320
Glycogen	Thermo Scientific	11883933
HEPES	Sigma-Aldrich	H3375
Hydrochloric acid	Fisher Chemical	10053023
L-Arginine	Thermo Scientific	A15738.14
L-Aspartic acid	Sigma-Aldrich	A8949
L-Cysteine	Thermo Scientific	J63745.14
L-Glutamic acid potassium salt monohydrate (potassium glutamate, KGlu)	Sigma-Aldrich	G1149
L-Histidine	Sigma-Aldrich	H6034
L-Isoleucine	Thermo Scientific	J63045.14
L-Leucine	Sigma-Aldrich	L8000
L-Lysine	Sigma-Aldrich	L5501
L-Methionine	Sigma-Aldrich	M5308
L-Phenylalanine	Thermo Scientific	J63925.22
L-Proline	Thermo Scientific	A10199.14
L-Serine	Thermo Scientific	J62187.09
L-Threonine	Thermo Scientific	J63709.30
L-Tryptophan	Sigma-Aldrich	T0254
L-Tyrosine	Thermo Scientific	J63511.09
L-Valine	Thermo Scientific	J62943.06
Lambda Exonuclease	New England Biolabs	M0262
Lyticase from *Arthrobacter luteus*	Sigma-Aldrich	L2524
M13mp18 Single-stranded DNA	New England Biolabs	N4040S
Magnesium acetate (Mg(OAc)_2_)	Sigma-Aldrich	M5661
Maleic acid	Sigma-Aldrich	M0375
N-Lauroylsarcosine sodium salt solution	Sigma-Aldrich	61747
NBT/BCIP Stock Solution	Roche	11681451001
Nonidet P40 Substitute (NP-40-S)	Roche	11754599001
Nt.BbvCI	New England Biolabs	R0632
NTP set (ATP, CTP, GTP, UTP)	Thermo Scientific	R0481
PageRuler™ Plus Prestained Protein Ladder, 10 to 250 kDa	Thermo Scientific	26619
Pepsin from porcine gastric mucosa	Sigma-Aldrich	P6887
Phenol:Chloroform:Isoamyl Alcohol 25:24:1 Saturated with 10 mM Tris, pH 8.0, 1 mM EDTA	Sigma-Aldrich	P2069
Ponceau S Staining Solution	Thermo Scientific	A40000279
Potassium chloride (KCl)	Fisher Chemical	10375810
Potassium hydroxide (KOH)	Sigma-Aldrich	221473
Potassium phosphate dibasic	Thermo Scientific Acros	10509263
Propidium Iodide	Biotium	40017
Proteinase K	New England Biolabs	P8107S
PstI-HF	New England Biolabs	R3140S
rCutSmart Buffer	New England Biolabs	B6004S
Ribonuclease A (RNaseA)	Sigma-Aldrich	R4875
Sepharose 4B gel matrix	Sigma-Aldrich	4B200
Skim Milk Powder	Sigma-Aldrich	70166
SmaI	New England Biolabs	R0141
Sodium acetate (NaOAc)	Fisher Chemical	10794761
Sodium chloride	Fisher Chemical	10616082
Sodium dodecyl sulfate (SDS)	Sigma-Aldrich	L3771
Sodium hydroxide (NaOH)	VWR	191373 M or 28244.262
Sodium phosphate dibasic	Sigma-Aldrich	04276
Sodium phosphate monobasic	Sigma-Aldrich	S0751
Sorbitol	Formedium	SOR02
Sucrose	Fisher Chemical	10346150
SYBR™ Safe DNA Gel Stain	Invitrogen	S33102
T4 DNA ligase	New England Biolabs	M0202M
Tri-sodium citrate dihydrate	Fisher Chemical	10396430
Trichloroacetic acid	Sigma-Aldrich	T6399
Trizma base	Sigma-Aldrich	T1503
TWEEN-20	VWR	663684B
Uracil	Sigma-Aldrich	U1128
Vidarabine (araA)	Cayman Chemical	18149
Vidarabine triphosphate (araATP)	Jena Biosciences	NU-1111
Xylene cyanol FF	Sigma-Aldrich	335940
Yeast Nitrogen base without Amino acids and without Thiamine	Formedium	CYN4701
Yeast Nitrogen base without Amino acids, without Ammonium sulphate and without Thiamine	Formedium	CYN4801
Cdc45	Yeeles et al, 2015	N/A
Cdc6	Frigola et al, 2013	N/A
Cdt1.Mcm2-7	Coster et al, 2014	N/A
Csm3/Tof1		N/A
Ctf18-RFC	Fletcher et al, 2025	N/A
Ctf4	Yeeles et al, 2015	N/A
DDK	On et al, 2014	N/A
Dpb11	Yeeles et al, 2015	N/A
GINS	Yeeles et al, 2015	N/A
Mcm10	Yeeles et al, 2015	N/A
Mrc1	Baretić et al, 2020	N/A
ORC	Frigola et al, 2013	N/A
PCNA	Yeeles et al, 2017	N/A
Pol ε	Yeeles et al, 2015	N/A
Pol δ	Yeeles et al, 2017	N/A
Pol α-primase	Yeeles et al, 2015	N/A
Pol ε^exo-^	Guilliam and Yeeles, 2021	N/A
Pol δ^exo-^	This study	N/A
Pol ε^P301R^	This study	N/A
Pol δ^POL-DEAD^	Aria and Yeeles, 2019	N/A
Pol ε^ΔCAT^	Yeeles et al, 2017	N/A
RFC	Yeeles et al, 2017	N/A
RPA	Baretić et al, 2020	N/A
S-CDK (truncated Clb5, Δ1-100)	Hill et al, 2020	N/A
Sld2	Yeeles et al, 2015	N/A
Sld3/7	Yeeles et al, 2015	N/A
Top1	Yeeles et al, 2015	N/A
**Oligonucleotides and other sequence-based reagents**		
See Appendix Table S1		
**Software**		
Fiji	Schindelin et al, 2012	https://fiji.sc/
Graphpad Prism 10 (v.10.3.0)	GraphPad	N/A
FlowJo (v10.10.1)	Waters Biosciences	https://www.flowjo.com/
EPSON Scan 2 (v.6.6.45.0)	Seiko Epson Corporation	N/A
BD FACSDiva (v8.0)	Waters Biosciences	N/A
Excel Version (v. 16.110.1)	Microsoft	

### DNA templates for replication reactions with purified budding yeast proteins

Replication reactions were carried out using the ARS306 ZN3 plasmid (Taylor and Yeeles, 2018). Modified forms of this plasmid were generated to introduce either an embedded araC, gemC, artificial abasic site or deoxycytidine (control) into the leading-strand template ~3 kbp left of ARS306 (Figs. 4 and EV4C). Briefly, ZN3 plasmid was modified to contain ZN3_Top1_3 duplex (Westhorpe et al, 2024) ligated into BamHI and PstI sites (vRW31). This was nicked with Nt.BbvCI, incubated with competitor oligo oRW25 and passed over a Sepharose 4B gel matrix to yield gapped DNA which was concentrated 10-fold, as previously described (Westhorpe et al, 2024, Appendix Fig. S1A). 150 μg gapped DNA was mixed with a 20-fold molar excess of either oHO106 (araC) or oHO107 (gemC) (synthesised by ATDBio), oHO113 (abasic) (synthesised by IDT), or oRW18 (control). Samples were then treated with T4 DNA Ligase (Appendix Fig. S1B,C) and the DNA isolated as previously described (Westhorpe et al, 2024).

All plasmids were caesium chloride gradient purified as in (Taylor and Yeeles, 2018). Reactions were performed using the following templates:

Experiments presented in Fig. 1D, EV1D, and EV2C were performed using circular ZN3.

Experiments presented in Figs. 1F,G, 2, 3, EV2D,E, EV3, and EV4A,B were performed using AhdI-linearised ZN3 (Taylor and Yeeles, 2018).

Experiments presented in Figs. 4 and EV4C were performed using control, araC, gemC, and abasic versions of AhdI-linearised vRW31.

### Standard replication reactions with purified budding yeast proteins

Replication reactions were performed as previously described (Taylor and Yeeles, 2018) with minor alterations. MCM loading and phosphorylation was performed in a reaction (50 μL or less) containing: 25 mM HEPES-KOH, pH 7.6; 100 mM KGlu; 0.01% NP-40-S; 1 mM DTT; 10 mM Mg(OAc)_2_; 0.1 mg/mL bovine serum albumin; 40 mM KCl; 3 mM ATP; 3 nM DNA template; 75 nM Cdt1.Mcm2-7; 40 nM Cdc6; 20 nM ORC; and 25 nM DDK (or 50 nM in Figs. 1D, 2B, EV1D, and EV2C). Following incubation at 24 °C for 10 min, S-CDK was added to 80 nM and incubation at 24 °C continued for a further 5 min. Reactions were then diluted fourfold into replication buffer, to give final replication reaction conditions of: 25 mM HEPES-KOH, pH 7.6; 250 mM KGlu; 0.01% NP-40-S; 1 mM DTT; 10 mM Mg(OAc)_2_; 0.1 mg/mL bovine serum albumin; 10 mM KCl; 0.75 nM indicated DNA template; 3.2 mM ATP; 0.2 mM C/G/UTP; 30 μM dA/dC/dG/dTTP; 18.75 nM Cdt1.Mcm2-7; 10 nM Cdc6; 6.25 nM DDK (or 12.5 nM); 5 nM ORC; and 33 nM [α-^32^P] dCTP (or [α-^32^P] dTTP, Fig. EV4A,B). Reactions were split and, where indicated, araA/araCTP or gemCTP was added to the written final concentration. DNA replication was initiated by addition of replication proteins from a master mix to final concentrations of: 30 nM Dpb11; 100 nM GINS; 30 nM Cdc45; 10 nM Mcm10; 20 nM Pol ε; 20 nM Ctf4; 100 nM RPA; 20 nM Csm3/Tof1; 20 nM RFC; 20 nM PCNA; 20 nM Pol α-primase; 10 nM Mrc1; 20 nM Sld2; 12.5 nM Sld3/7 and, unless otherwise indicated, 10 nM Pol δ and reactions were transferred to 30 °C. Reactions with circular ZN3 template additionally contained 20 nM Top1, and indicated reactions in Fig. 1D additionally contained 20 nM Ctf18-RFC. Pol δ buffer (Appendix Table S2) was used in reactions omitting Pol δ. Where indicated, polymerase mutants were added immediately before addition of the protein master mix. Reactions were quenched by addition of EDTA, pH 8.0 to 50 mM at the indicated timepoints.

### Pulse-chase replication reactions with purified budding yeast proteins

Pulse-chase reactions were performed as described for standard replication reactions, except the dCTP concentration during the pulse was lowered to 2.5 μM. During the chase, the dCTP concentration was raised to 600 μM.

### Primer extension reactions

Primed M13mp18 ssDNA template was prepared using the oligonucleotide JY180 as previously described (Fletcher et al, 2025). For primer extension reactions, 1.5 nM ssDNA template was incubated with 600 nM RPA at 30 °C for 10 min in a reaction (~125 μL) containing: 25 mM HEPES-KOH, pH 7.6; 100 mM KGlu; 10 mM Mg(OAc)_2_; 0.01% NP-40-S; 1 mM DTT; 0.1 mg/mL bovine serum albumin; 750 μM ATP; 30 μM dA/dGTP. Proteins were then added to final concentrations of: 20 nM PCNA; 5 nM RFC; 20 nM either Pol ε, Pol ε^exo-^, or Pol δ. Reactions were split (56 μL) and, where indicated, araATP was added to a final concentration of 10 μM and incubated for a further 5 min at 30 °C. DNA replication was initiated by addition of nucleotides from a master mix to final concentrations of: 33 nM [α-^32^P] dCTP, 30 μM dC/dTTP and transferred to 30 °C before quenching by addition of EDTA, pH 8.0 to 50 mM at the indicated timepoints. Reactions with Pol α were performed as above except were initiated by addition of 10 nM Pol α, contained 1 nM ssDNA template, 400 nM RPA, 80 μM dA/dC/dG/dTTP, 500 μM ATP (final concentrations) and did not contain RFC.

### Post-reaction sample processing

For native agarose gel analysis, samples were deproteinized by treatment with Proteinase K (20 units/mL) and SDS (0.25%) at 37 °C for 20 min, followed by phenol:chloroform:isoamyl alcohol (25:24:1) (PCI) extraction. Unincorporated radionuclide was removed using MicroSpin G-50 columns (Cytiva). 1/5 volume 5× native loading buffer (20 mM Tris-HCl, pH 8.0; 50 mM EDTA, pH 8.0; 10% Ficoll 400; 2% N-Lauroylsarcosine sodium salt solution) was added to samples before running on 1% modified TAE agarose gels (50 mM Tris-HCl, pH 7.9; 40 mM sodium acetate; 1 mM EDTA, pH 8.0) at 21 V overnight in electrophoresis buffer (50 mM Tris-HCl, pH 7.9; 40 mM sodium acetate; 1 mM EDTA, pH 8.0). Gels were dried onto Whatman 3MM paper (Cytiva) at 75 °C under vacuum, exposed on BAS-IP MS Storage Phosphor Screens (Cytiva), and imaged using a Typhoon phosphorimager (GE Healthcare).

For denaturing agarose gel analysis, 1/10 volume 10X alkaline loading buffer (10% sucrose; 500 mM NaOH; xylene cyanol in water) was added to samples before running on 0.7% alkaline agarose gels (30 mM NaOH, 2 mM EDTA) at 24 V for 16.5 h in alkaline electrophoresis buffer (30 mM NaOH, 2 mM EDTA) before radionuclide removal as above. Gels were additionally fixed at 4 °C with two washes in 5% trichloroacetic acid before neutralisation with 1 M Tris-HCl, pH 8.0 before being dried and imaged as above.

Following deproteinization, PCI extraction and radionuclide removal as above, reactions in Fig. 4B were additionally digested with SmaI (20 units/mL) in 1× rCutSmart buffer at 37 °C for 30 min before being analysed by denaturing agarose gel electrophoresis.

### Replication protein expression and purification

All proteins were expressed and purified as described previously. A list of proteins used in this study, their affinity tags, purification strategies, and final buffers are detailed in Appendix Table S2. Pol ε^P301R^ was obtained by site-directed mutagenesis of pAJ6 (Yeeles et al, 2015) using oligonucleotides oHO50 and oHO51 to produce vHO21. yVA2 (Aria and Yeeles, 2019) was transformed sequentially with linearised pRS306/*DPB2- pGAL-1-10- DPB3* (Yeeles et al, 2015), and linearised vHO21 to generate yHO49. Pol δ^exo-^ (D321A, E323A) was obtained by site-directed mutagenesis of pRS306/*POL3- pGAL-1-10- POL31* (Yeeles et al, 2017) using oligonucleotides oHO9 and oHO10 to produce vHO14. yJF1 (Frigola et al, 2013) was transformed sequentially with linearised pRS303/*POL32-CBP- pGAL-1-10- GAL4* (Yeeles et al, 2017) and linearised vHO14 to produce yHO33. Nanopore sequencing verified the presence of only intended mutations (Source Bioscience). Pol ε^P301R^ was purified following the method described for Pol ε mutants in (Aria and Yeeles, 2019). Pol δ^exo-^ was purified following the method described for Pol δ in (Yeeles et al, 2017) but eluted in a different final storage buffer (Appendix Table S2).

### Budding yeast strain construction

Yeast strains used in this work are listed in Reagents and Tools Table. When grown in the presence of either araA, araC, or gemC, strains were grown in the indicated synthetic medium without thiamine to avoid possible competition with araA for uptake (Iwashima et al, 1992) (see Appendix Table S3 for media details). *hENT1* (under the control of the *S. cerevisiae GAP* promoter) and *HSV-TK* (under the control of the *S. cerevisiae ADH1* promoter) genes were integrated at the *ura3-1* locus following transformation of linearised p306-BrdU-Inc plasmid (Viggiani and Aparicio, 2006) (yPZ4587). Synthetic *ADK* and *DCK* genes (codon-optimised for expression in *S. cerevisiae*, bPZ1352) were synthesised by Genscript and subcloned into pATP403red vector (Tominaga et al, 2021) to generate an *ADK* cassette under the control of the *S. cerevisiae TEF1* promoter and *CYC1* terminator, and a *DCK* cassette under the control of the *S.* *cerevisiae ADH1* promoter and terminator (bPZ1353). These cassettes were subcloned into the pRS305 vector using SacII and XhoI sites (vHO9) and integrated at the *leu2-3* locus following linearisation (yHO25).

*POL2* mutations were introduced by site-directed mutagenesis of vHO15 which contains the *POL2* gene amplified from W303-1A budding yeast ligated into PacI and AscI sites of pFA6a-hphNT1 vector upstream of the *hphNT1* cassette. The *pol2 exo-* allele (*pol2-D290A,E292A*) was generated using oligonucleotides oHO23 and oHO24 to produce vHO17. The *pol2-P301R* allele was generated using oligonucleotides oHO52 and oHO53 to produce vHO20. The *pol2-M644G* allele was generated using oligonucleotides oHO114 and oHO115 to produce vHO23. *POL3* mutations were introduced by site-directed mutagenesis of vHO16 which contains the *POL3* gene amplified from W303-1A budding yeast ligated into PacI and AscI sites of pFA6a-natNT2 vector upstream of the *natNT2* cassette. The *pol3-5DV* allele was generated using oligonucleotides oHO39 and oHO54 to produce vHO19. The *pol3 exo-* allele was generated using oligonucleotides oHO25 and oHO26 to produce vHO18. The *pol3-L612G* allele was generated using oligonucleotides oHO188 and oHO189 to produce vHO28. The indicated alleles were introduced into strains at the endogenous locus by transformation of PCR products using either oHO43 and oHO44 (*POL2*), or oHO45 and oHO46 (*POL3*) using standard procedures. Diagnostic PCRs and nanopore sequencing verified the presence of only intended mutations (Source Bioscience). The addition of the *hphNT1* or *natNT2* cassette downstream of the *POL2* or *POL3* genes, respectively, had no effect on growth (Appendix Fig. S2).

To generate strains for polymerase usage assays (Figs. 6 and EV6), the chromosomal *URA3* gene (*ura3-1*, chr V) was first replaced with the *KanMX4* cassette amplified by PCR from pFA6a-KanMX4 vector using oHO136 and oHO137 (*ura3∆::KanMX4*). The *URA3* cassette was then introduced ~849 bp to the right of ARS306 (chr III) following transformation with PCR products from vJY201 using either oHO138 and oHO139 (*URA3*-OR1), or oHO140 and oHO141 (*URA3*-OR2). Where indicated, the *RNH201* gene was replaced with the *hphMX6* cassette following transformation with PCR product from pFA6a-hphMX6 vector using PZ2836 and PZ2837 (*rnh201∆::hphMX6*).

### Budding yeast growth assays

Overnight cultures from single colonies of indicated budding yeast strains were grown to 1  × 10^7^ cells/mL in YEPD medium at 25 °C. Cells were then washed in sterile Milli-Q water, consecutively diluted threefold six times and transferred using a pronged spotter onto minimal media (MM) -thiamine -adenine agar plates containing either DMSO or DMSO-solubilised araA, araC, or gemC at the written concentrations. Where indicated, uracil and leucine were also omitted. Plates were incubated at the indicated temperature for 5 days and imaged approximately every 24 h with an Epson Perfection V850 Pro scanner using EPSON Scan 2 software (Seiko Epson Corporation). Growth of the ninefold diluted spot was quantified for each strain using FIJI as described in (Petropavlovskiy et al, 2020, and Appendix Fig. S3) and plotted as a percentage relative to growth on DMSO after 5 days using Graphpad Prism 10.

### Cell synchrony experiments

Overnight cultures from single colonies of indicated budding yeast strains were grown to 1  × 10^7^ cells/mL in YEPD medium at 25 °C. Cultures were arrested in G1-phase at 30 °C by addition of water-solubilised α-factor mating pheromone to 10 μg/mL for 75 min, before being re-added for a further 40 min. α-factor was then added to 2.5 μg/mL every 20 min until ≤10% cells were budded, as determined cytologically. Cells were released from arrest by washing with synthetic complete–thiamine–adenine–uracil–leucine (SC) medium without α-factor. Cells were then released into SC medium containing either DMSO or 100 μM DMSO-solubilised araA. For subsequent G1 arrests within the same experiment (Fig. 5E–G), cells were washed and resuspended in SC medium containing 10 μg/mL α-factor and left at 30 °C for 40 min. α-factor was then added to 2.5 μg/mL every 20 min until indicated, before being washed with and released into SC medium without α-factor.

### Flow cytometry

Approximately 1 × 10^7^ cells were pelleted and fixed in 70% ethanol at 4 °C for at least 2 h before being processed and stained as previously described (Jenkinson et al, 2023). Samples were diluted and sonicated immediately before being analysed on a LSRFortessa (Waters Biosciences). In total, 5000 ungated events were recorded per sample using the YG 585-A detector. Histograms were manually gated and visualised using FlowJo (Appendix Fig. S4).

### Protein extraction and western blotting

Protein was extracted from approximately 1 × 10^8^ pelleted yeast cells by bead beating at 4 °C with 0.5 mm glass beads (Sigma-Aldrich) in 20% trichloroacetic acid using a Precellys tissue homogeniser (Bertin Technologies). The precipitate was pelleted before being resuspended in Laemmli buffer (62.5 mM Tris-HCl, pH 6.8; 0.5% SDS; 10% glycerol; bromophenol blue; 5% 2-mercaptoethanol) and neutralised with Tris-HCl, pH 9.5 before boiling. For Rad53 (Fig. 5D,G), samples were analysed on NuPAGE 7% Tris-Acetate protein gels (Invitrogen). For ADK and DCK (Fig. EV5F), NuPAGE 4–12% Bis-Tris protein gels (Invitrogen) were used. Samples were transferred onto a PVDF membrane via the iBlot 2 gel transfer device (Invitrogen) following manufacturer’s instructions. Membranes were stained for 5 min with Ponceau S, imaged with an Epson Perfection V850 Pro scanner before being blocked in 5% skim milk in Tris-buffered saline + 0.1% TWEEN-20 (TBS-T) for 1 h at room temperature. Proteins were detected by immunoblotting using the following primary antibody dilutions (v:v) in 5% skim milk in TBS-T: anti-ADK 1:20,000; anti-DCK 1:20,000; anti-Rad53 1:2000. Membranes were then incubated with rabbit horseradish peroxidase-conjugated secondary antibody at 1:10,000 dilution. Blots were visualised using ECL (Cytiva) and developed onto Medical X-ray Film Super RX (Fujifilm) before being imaged using an Epson Perfection V850 Pro scanner.

### ssDNA probe generation for Southern blotting

Digoxygenin (DIG)-labelled ssDNA probe was made using PCR DIG Probe Synthesis Kit (Roche) with oligos oHO206 and oHO207 and vJY201 as a template, following manufacturer’s instructions. The PCR product was purified using QIAquick PCR Purification Kit (Qiagen), quantified by Tapestation D1000 DNA ScreenTape analysis (Agilent), and treated with Lambda Exonuclease (10 units/μg DNA, 200 μL reaction) at 37 °C to specifically degrade the oHO206 strand. After 40 min, reactions were quenched by adding EDTA, pH 8.0 to 50 mM and heat inactivated at 75 °C for 10 min.

### Alkaline treatment of genomic DNA and southern blotting

Overnight cultures from single colonies of indicated budding yeast strains were grown in YEPD medium at 25 °C (yHO105, yHO133, yHO134, yHO137, yHO148) or 30 °C (yHO136, yHO140, yHO142, yHO144, yHO145, yHO147). In total, 2.5 × 10^8^ pelleted cells were resuspended in 50 mL SC -thiamine -ade media (0.5 × 10^7^ cells/mL) containing either DMSO or araA at the indicated concentration. Cells were incubated at 30 °C, 200 rpm for either 30 (Fig. EV6B–E) or 31 h (Fig. 6D–F) before being pelleted, washed with PBS, frozen on dry ice, and stored at −80 °C. gDNA was isolated using 100/G Genomic-tip and the associated genomic DNA buffer set (Qiagen) following manufacturer’s instructions for yeast samples. Lyticase (solubilised in 1.2 M sorbitol; 100 mM sodium phosphate, pH 7.4; 715 mM 2-mercaptoethanol) was added to degrade yeast cell walls. DNA was analysed on 0.5% TAE agarose gels in 1× TAE buffer stained with SYBR Safe (Invitrogen). Unless otherwise stated, 4 μg DNA was precipitated with 0.3 M NaCl, 40 μg glycogen and 2 volumes cold 100% ethanol, before being washed with room temperature 70% ethanol and solubilised in water. Samples were alkali treated with 0.3 M KOH (40 μL reactions) and run through 0.7% alkaline agarose gels which were subsequently neutralised and stained with 0.5 μg/mL ethidium bromide as described in (McElhinny et al, 2010) before being imaged using a ChemiDoc MP Imaging System (Bio-Rad).

Gels were then depurinated (0.4 M HCl) for 10 min and denatured (0.5 M NaOH) for 30 min at room temperature before being transferred onto Hybond-N+ membranes (Cytiva) by capillary blotting in 20× SSC (3 M NaCl, 0.34 M trisodium citrate, pH 7.0) overnight. Membranes were dried onto Whatman 3MM paper, crosslinked at 120 mJ/cm^2^ using a UVP Crosslinker CL-3000 (Analytik Jena) and then equilibrated in pre-warmed DIG Easy Hyb (Roche) in hybridisation tubes (Cole-Parmer) in a HB-1D hybridiser oven (Techne) at 40 °C for 3 h. ssDNA probe was diluted in DIG Easy Hyb (Roche) to a final concentration of either 40 ng/mL (Fig. 6C) or 25 ng/mL (Figs. 6F and EV6E) and incubated with the membrane in hybridisation tubes at 40 °C for 18 h. The membrane was then washed twice for 5 min at room temperature with 2× SSC, 1% SDS; washed twice for 15 min with pre-warmed 1× SSC, 0.1% SDS at 65 °C; then washed once for 5 min at room temperature with 100 mM maleic acid; 150 mM NaCl, made to pH 7.5; 0.3% TWEEN-20 (Maleic Acid Buffer + 0.3% TWEEN-20) before blocking with 1X blocking solution as part of the DIG Luminescent Detection Kit (Roche) for 30 min at room temperature. The membrane was then incubated with anti-DIG-AP (1:5000 in 1× blocking solution) for another 30 min before two further 15-min washes at room temperature with Maleic Acid Buffer + 0.3% TWEEN-20. After a brief wash with 100 mM Tris-HCl, pH 7.4; 100 mM NaCl; made to pH 9.5 (Detection Buffer), signal was detected by adding NBT/BCIP (1:50 in detection buffer) directly onto the membrane. After overnight exposure, membranes were imaged using a ChemiDoc MP Imaging System.

Samples in Figs. 6B,C and EV6A were processed as above but were extracted from 50 mL YEPD cultures (at 1 × 10^7^ cells/mL) using Monarch HMW DNA Extraction Kit for Tissue (New England Biolabs), following manufacturer’s instructions for yeast samples using Lyticase (as above), and run through 1% alkaline agarose gels.

### Molecular weight markers

All replication reactions (and Fig. EV6A) used λ DNA-HindIII Digest as size standards, prepared as described in (Taylor and Yeeles, 2018). Western blots used PageRuler Plus Prestained Protein Ladder; native agarose gels used 1 Kb Plus DNA Ladder; and Southern blots used DIG-labelled DNA Molecular Weight Marker VII.

### Quantification and statistical analysis

Gel lanes were quantified using FIJI and intensity was plotted against molecular weight marker migrations using Graphpad Prism 10. Lane profiles for Figs. 1F, 1G, 2A, 2B, 2D, 3A, 3B, and 3C were generated using raw intensity values. Intensity values in lane profiles for Figs. 4B, 6C,F were normalised relative to either the right leading-strand product or overall lane signal, respectively, using Excel. Maximal leading-strand synthesis rates for Fig. 2E were calculated as described in (Fletcher et al, 2025). Statistical analysis was performed using Graphpad Prism 10 as described in figure legends.

## Supplementary information

Appendix

Peer Review File

Source data Fig. 1

Source data Fig. 2

Source data Fig. 3

Source data Fig. 4

Source data Fig. 5

Source data Fig. 6

Figure Source Data EV1

Figure Source Data EV2

Figure Source Data EV3

Figure Source Data EV4

Figure Source Data EV5

Figure Source Data EV6

Figure Source Data Appendix

Expanded View Figures

## Data Availability

This study includes no data deposited in external repositories. The source data of this paper are collected in the following database record: biostudies:S-SCDT-10_1038-S44318-026-00901-1.
