# Supplementary material for: Response of the replisome to araA and araC suggests a mechanism for arabinosyl nucleoside tolerance
Source: EMBO J. 2026 Aug 27;45(20):7191–225. doi: 10.1038/s44318-026-00901-1 (PMC13672804; doi:10.1038/s44318-026-00901-1)
Supplement: Supplementary file 1 [file 44318_2026_901_MOESM16_ESM.pdf]

Expanded View Figures

Figure EV1. Pol ε proofreading restricts araATP-induced helicase-polymerase uncoupling.

Related to Fig. 1. (**A**) Schematic representation of in vitro primer extension assay on singly primed ssDNA template. (**B**, **C**) Denaturing gel autoradiography of primer extension reactions performed with the indicated polymerase as in (**A**). (**D**) Denaturing (top) and native gel (bottom) autoradiography of replication reactions performed as in Fig. 1C. The native gel was overexposed to aid visualisation of weaker U* species. Source data are available online for this figure.

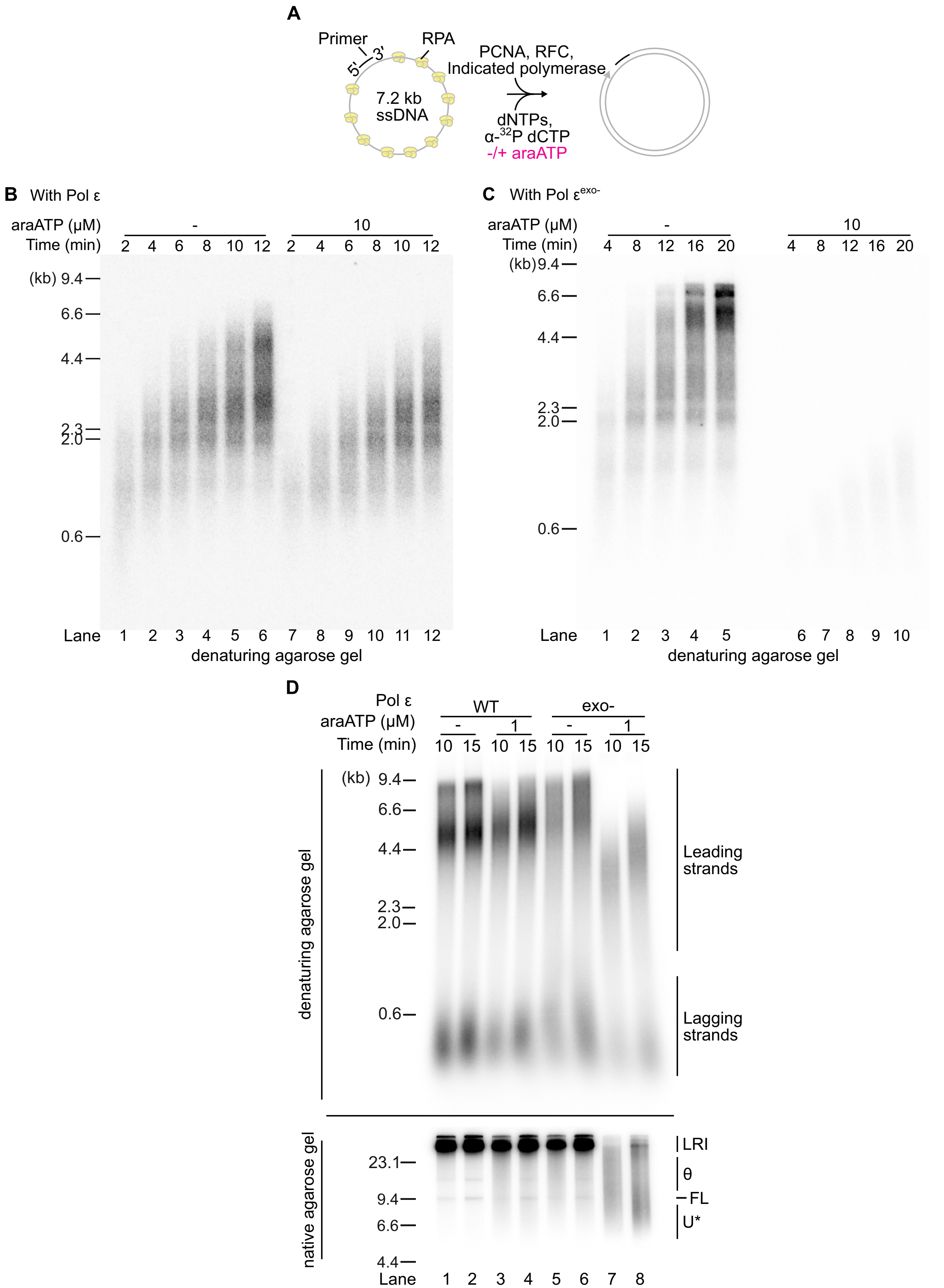
A
Primer
5'
3'
RPA
7.2 kb
ssDNA
PCNA, RFC,
Indicated polymerase
dNTPs,
α-32P dCTP
-/+ araATP
B With Pol ε
araATP (μM)
-
10
Time (min)
2 4 6 8 10 12 2 4 6 8 10 12
(kb) 9.4
6.6
4.4
2.3
2.0
0.6
Lane 1 2 3 4 5 6 7 8 9 10 11 12
denaturing agarose gel
C With Pol εexo-
araATP (μM)
-
10
Time (min)
4 8 12 16 20 4 8 12 16 20
(kb)9.4
6.6
4.4
2.3
2.0
0.6
Lane 1 2 3 4 5 6 7 8 9 10
denaturing agarose gel
D
Pol ε
WT
exo-
araATP (μM)
-
1
-
1
Time (min)
10 15 10 15 10 15 10 15
(kb) 9.4
6.6
4.4
2.3
2.0
0.6
denaturing agarose gel
Leading strands
Lagging strands
native agarose gel
23.1
9.4
6.6
4.4
LRI
θ
FL
U*
Lane 1 2 3 4 5 6 7 8

 © The Author(s)

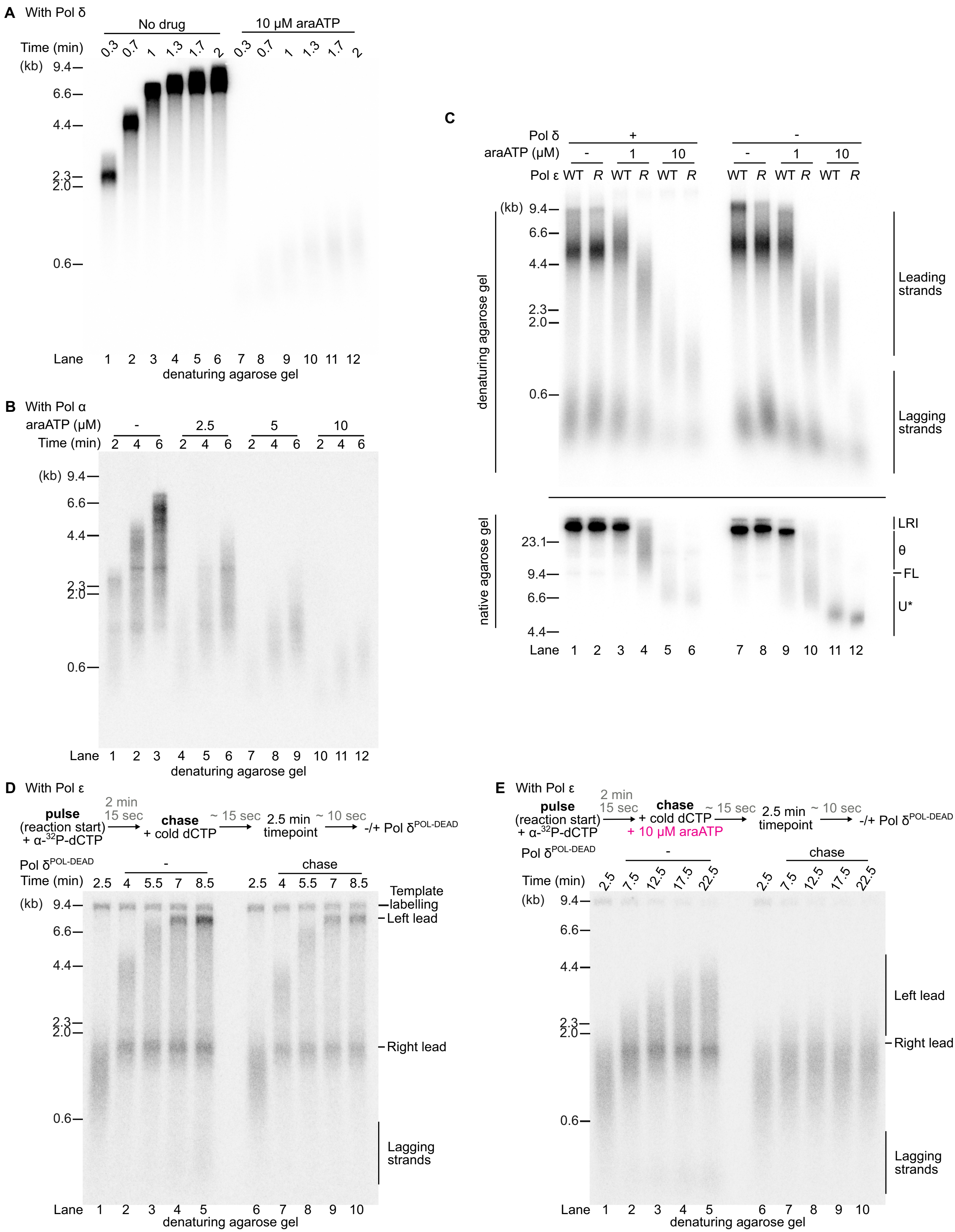
A With Pol δ
No drug
10 μM araATP
Time (min) 0.3 0.7 1 1.3 1.7 2 0.3 0.7 1 1.3 1.7 2
(kb) 9.4 6.6 4.4 2.3 2.0 0.6
Lane 1 2 3 4 5 6 7 8 9 10 11 12
denaturing agarose gel
B With Pol α
araATP (μM) - 2.5 5 10
Time (min) 2 4 6 2 4 6 2 4 6 2 4 6
(kb) 9.4 6.6 4.4 2.3 2.0 0.6
Lane 1 2 3 4 5 6 7 8 9 10 11 12
denaturing agarose gel
C
Pol δ + -
araATP (μM) - 1 10 - 1 10
Pol ε WT R WT R WT R WT R WT R WT R
(kb) 9.4 6.6 4.4 2.3 2.0 0.6
denaturing agarose gel
Leading strands
Lagging strands
native agarose gel
23.1 9.4 6.6 4.4
LRI
θ
FL
U*
Lane 1 2 3 4 5 6 7 8 9 10 11 12
D With Pol ε
pulse (reaction start) + α-32P-dCTP
2 min 15 sec
chase + cold dCTP
~ 15 sec
2.5 min timepoint
~ 10 sec
-/+ Pol δPOL-DEAD
Pol δPOL-DEAD - chase
Time (min) 2.5 4 5.5 7 8.5 2.5 4 5.5 7 8.5
(kb) 9.4 6.6 4.4 2.3 2.0 0.6
Template labelling
Left lead
Right lead
Lagging strands
Lane 1 2 3 4 5 6 7 8 9 10
denaturing agarose gel
E With Pol ε
pulse (reaction start) + α-32P-dCTP
2 min 15 sec
chase + cold dCTP + 10 μM araATP
~ 15 sec
2.5 min timepoint
~ 10 sec
-/+ Pol δPOL-DEAD
Pol δPOL-DEAD - chase
Time (min) 2.5 7.5 12.5 17.5 22.5 2.5 7.5 12.5 17.5 22.5
(kb) 9.4 6.6 4.4 2.3 2.0 0.6
Left lead
Right lead
Lagging strands
Lane 1 2 3 4 5 6 7 8 9 10
denaturing agarose gel

Figure EV2. Additional evidence for the role of Pol δ on the leading strand in response to araATP.

Related to Fig. 2. (**A**) Denaturing gel autoradiography of primer extension reaction performed as in Fig. EV1A with Pol δ. The image was overexposed to aid visualisation of weaker synthesis products (lanes 7–12). (**B**) Denaturing gel autoradiography of primer extension reaction performed as in (A) except with Pol α and without RFC. (**C**) Denaturing (top) and native gel (bottom) autoradiography of replication reactions performed as in Fig. 1C. Reactions were quenched after 10 min. Pol ε R = Pol ε^{P301R}. The native gel was overexposed to aid visualisation of weaker U* species. (**D**, **E**) Pulse-chase replication reactions performed on AhdI-linearised dsDNA template as indicated (and see "Methods") and analysed by denaturing gel autoradiography. Where indicated, araATP was added during the chase to a final concentration of 10 µM after all components were added. Source data are available online for this figure.

 © The Author(s)

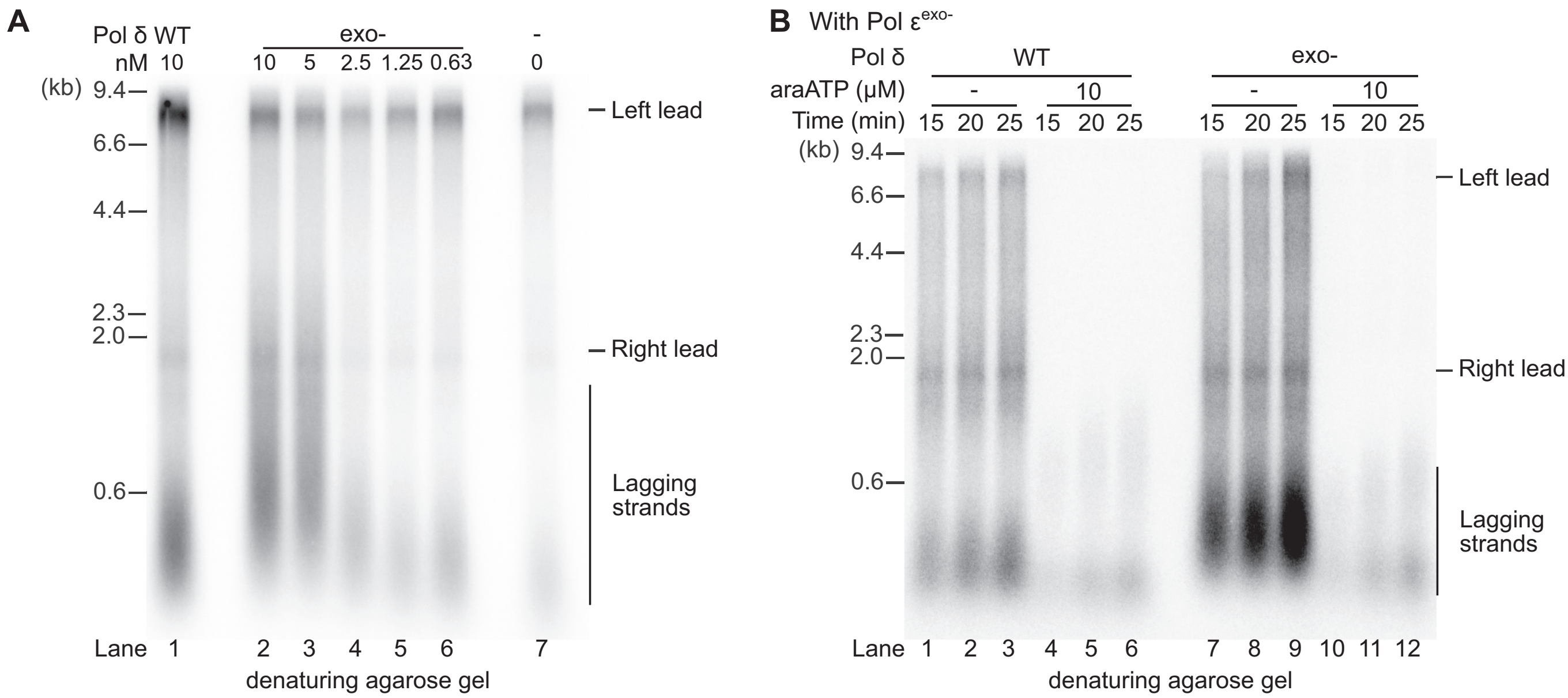

Figure EV3. Pol δ^{exo-} strand-displacement activity is suppressed at lower concentrations.

Related to Fig. 3. (**A**) Denaturing gel autoradiography of replication reactions performed on AhdI-linearised version dsDNA template as in Fig. 1E with indicated concentrations of Pol δ^{exo-}. (**B**) Denaturing gel autoradiography of replication reactions performed as in Fig. 3B, but using fourfold less (2.5 nM) Pol δ^{exo-}. Source data are available online for this figure.

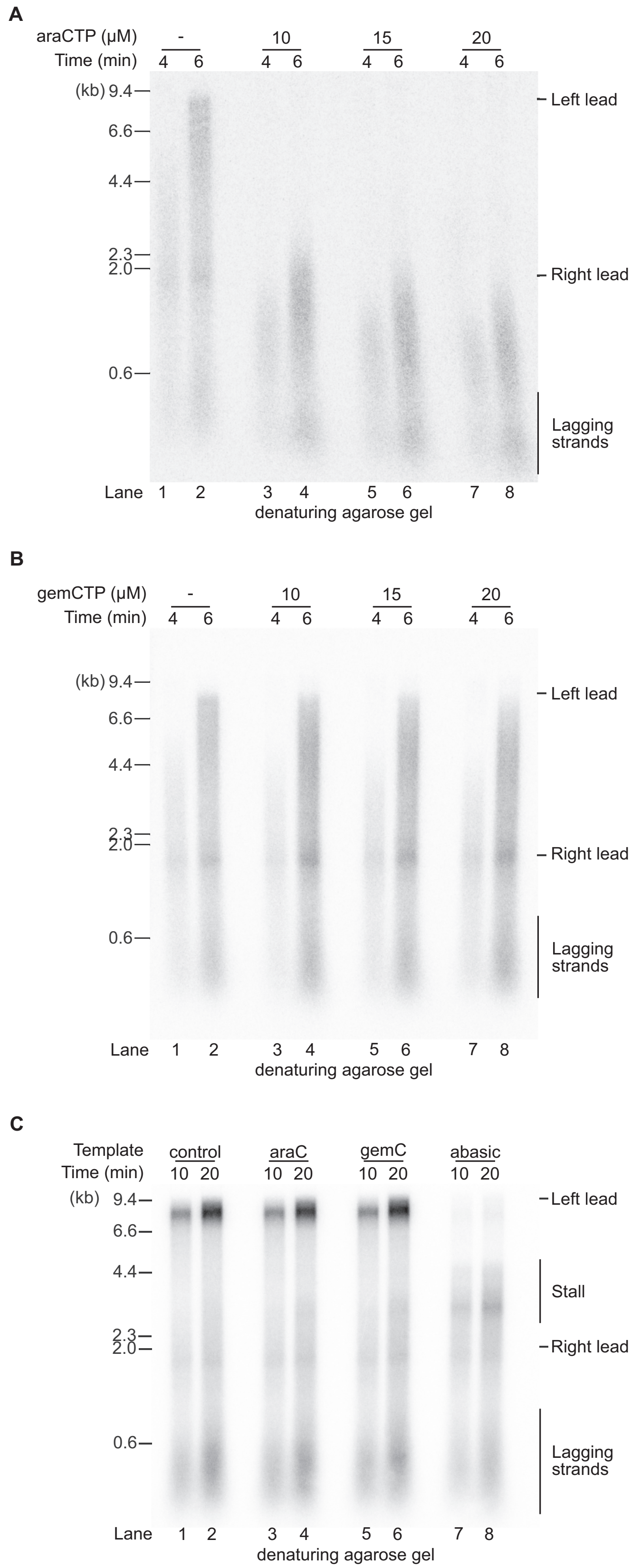
A
araCTP (μM)
-
10
15
20
Time (min)
4 6 4 6 4 6 4 6
(kb) 9.4
6.6
4.4
2.3
2.0
0.6
Left lead
Right lead
Lagging strands
Lane 1 2 3 4 5 6 7 8
denaturing agarose gel
B
gemCTP (μM)
-
10
15
20
Time (min)
4 6 4 6 4 6 4 6
(kb) 9.4
6.6
4.4
2.3
2.0
0.6
Left lead
Right lead
Lagging strands
Lane 1 2 3 4 5 6 7 8
denaturing agarose gel
C
Template
control
araC
gemC
abasic
Time (min)
10 20 10 20 10 20 10 20
(kb) 9.4
6.6
4.4
2.3
2.0
0.6
Left lead
Stall
Right lead
Lagging strands
Lane 1 2 3 4 5 6 7 8
denaturing agarose gel

 © The Author(s)

Figure EV4. Effects of araC and gemC on DNA synthesis when present in solution as triphosphates or embedded in the leading-strand template.

Related to Fig. 4. (**A**, **B**) Denaturing gel autoradiography of replication reactions with indicated concentrations of either araCTP (**A**) or gemCTP (**B**) performed on AhdI-linearised version dsDNA template as in Fig. 1E except α- ^{32}P dTTP radiolabel was used. (**C**) Denaturing gel autoradiography of replication reactions performed as in Fig. 4B, but without post-quench SmaI treatment. Source data are available online for this figure.

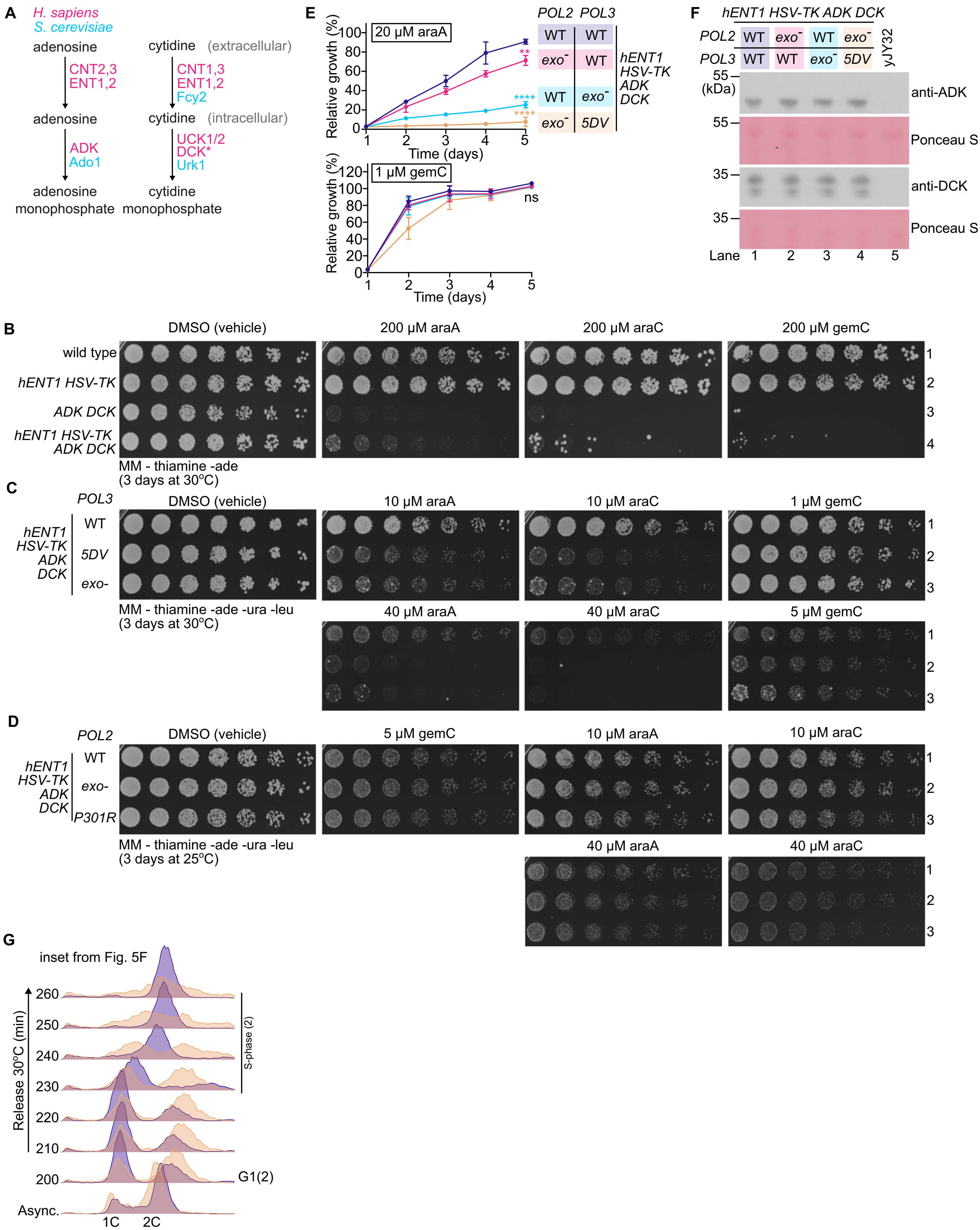
A
H. sapiens
S. cerevisiae
adenosine
CNT2,3
ENT1,2
adenosine
ADK
Ado1
adenosine monophosphate
cytidine (extracellular)
CNT1,3
ENT1,2
Fcy2
cytidine (intracellular)
UCK1/2
DCK*
Urk1
cytidine monophosphate
E
20 μM araA
Relative growth (%)
Time (days)
1 μM gemC
ns
POL2 POL3
WT WT
exo- WT
WT exo-
exo- 5DV
hENT1 HSV-TK ADK DCK
F
hENT1 HSV-TK ADK DCK
POL2 WT exo- WT exo-
POL3 WT WT exo- 5DV
yJY32
(kDa)
anti-ADK
Ponceau S
anti-DCK
Lane 1 2 3 4 5
B
DMSO (vehicle)
200 μM araA
200 μM araC
200 μM gemC
wild type
hENT1 HSV-TK
ADK DCK
hENT1 HSV-TK ADK DCK
MM - thiamine -ade
(3 days at 30°C)
C
POL3
WT
5DV
exo-
10 μM araA
10 μM araC
1 μM gemC
40 μM araA
40 μM araC
5 μM gemC
MM - thiamine -ade -ura -leu
(3 days at 30°C)
D
POL2
P301R
5 μM gemC
10 μM araA
10 μM araC
40 μM araA
40 μM araC
MM - thiamine -ade -ura -leu
(3 days at 25°C)
G
inset from Fig. 5F
Release 30°C (min)
S-phase (2)
G1(2)
Async.
1C
2C

© The Author(s)

Figure EV5. The role of proofreading in tolerating ara nucleosides in vivo.

Related to Fig. 5. (**A**) Schematic illustrating the human (pink) and yeast (blue) transporters and kinases involved in uptake and activation of adenosine and cytidine, respectively. *Whilst cytidine is not a preferred substrate for DCK, this kinase phosphorylates cytidine analogues. (**B**) Threefold serial dilutions of the indicated genotypes (yHO38, yPZ4587, yHO36, yHO25) spotted onto media with the indicated conditions. (**C**) Serial dilutions as in (**B**) of the indicated genotypes (yHO56, yHO66, yHO50). (**D**) Serial dilutions as in (**B**) of the indicated genotypes (yHO45, yHO43, yHO60). (**E**) Graphs quantifying growth over 5 days of strains as in Fig. 5A for indicated condition. Error bars represent standard deviation of three biological replicates. The measure of centre of the error bars represents the mean. Significance of differences relative to wild-type at 5-day timepoint was estimated by one-way ANOVA with Tukey's test for multiple comparisons, as in Fig. 5B. *P* values, additional comparisons and test summaries are shown in Appendix Table S4. (**F**) Western blots from strains in Fig. 5A alongside yJY32 (control) against ADK and DCK. (**G**) Zoomed-in view of flow cytometry profile presented in Fig. 5F. Source data are available online for this figure.

© The Author(s)

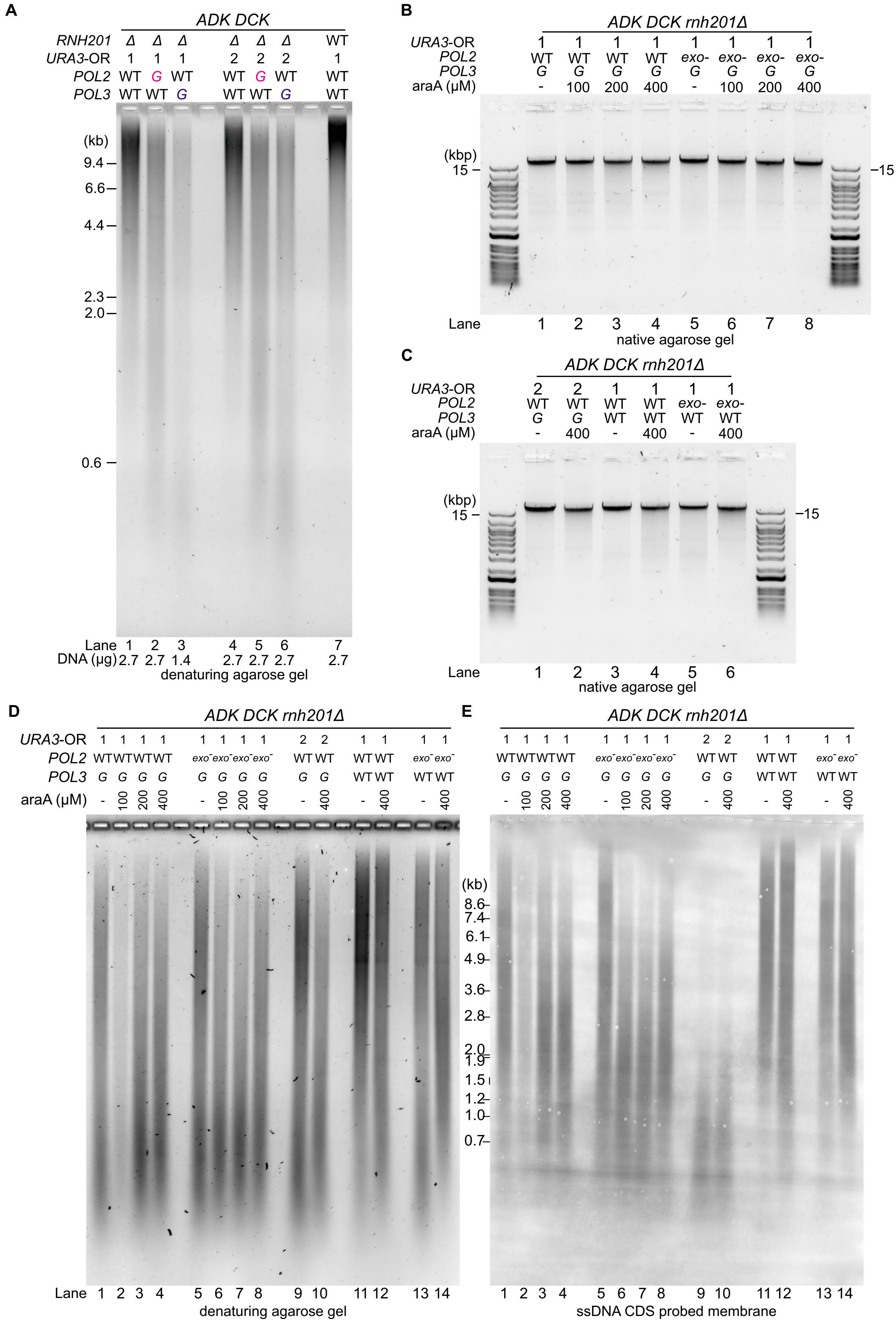
A
ADK DCK
RNH201 Δ Δ Δ Δ Δ Δ WT
URA3-OR 1 1 1 2 2 2 1
POL2 WT G WT WT G WT WT
POL3 WT WT G WT WT G WT
(kb)
9.4
6.6
4.4
2.3
2.0
0.6
Lane 1 2 3 4 5 6 7
DNA (μg) 2.7 2.7 1.4 2.7 2.7 2.7 2.7
denaturing agarose gel
B
ADK DCK rnh201Δ
URA3-OR 1 1 1 1 1 1 1 1
POL2 WT WT WT WT exo- exo- exo- exo-
POL3 G G G G G G G G
araA (μM) - 100 200 400 - 100 200 400
(kbp)
15
15
Lane 1 2 3 4 5 6 7 8
native agarose gel
C
ADK DCK rnh201Δ
URA3-OR 2 2 1 1 1 1
POL2 WT WT WT WT exo- exo-
POL3 G G WT WT WT WT
araA (μM) - 400 - 400 - 400
(kbp)
15
15
Lane 1 2 3 4 5 6
native agarose gel
D
ADK DCK rnh201Δ
URA3-OR 1 1 1 1 1 1 1 1 2 2 1 1 1 1
POL2 WT WT WT WT exo⁻ exo⁻ exo⁻ exo⁻ WT WT WT WT exo⁻ exo⁻
POL3 G G G G G G G G G G WT WT WT WT
araA (μM) - 100 200 400 - 100 200 400 - 400 - 400 - 400
Lane 1 2 3 4 5 6 7 8 9 10 11 12 13 14
denaturing agarose gel
E
ADK DCK rnh201Δ
1 1 1 1 1 1 1 1 2 2 1 1 1 1
WT WT WT WT exo⁻ exo⁻ exo⁻ exo⁻ WT WT WT WT exo⁻ exo⁻
G G G G G G G G G G WT WT WT WT
- 100 200 400 - 100 200 400 - 400 - 400 - 400
(kb)
8.6
7.4
6.1
4.9
3.6
2.8
2.0
1.9
1.5
1.2
1.0
0.7
1 2 3 4 5 6 7 8 9 10 11 12 13 14
ssDNA CDS probed membrane

 © The Author(s)

Figure EV6. Additional evidence for greater Pol δ utility on the leading strand in vivo in response to araA.

Related to Fig. 6. (**A**) Independent replicate of gel presented in Fig. 6B with addition of strain yHO105 (lane 7). Amount of DNA loaded is indicated under each lane. (**B**, **C**) Native gel analysis of gDNA from indicated strains (yHO142, yHO147, yHO144, yHO133, yHO148) treated with either DMSO (−) or araA. (**D**) Alkali-treated gDNA samples from (**B**, **C**) analysed by denaturing agarose gel electrophoresis and stained with ethidium bromide as in (**A**). (**E**) Southern blot of denaturing gel in (**D**) probed as in Fig. 6C. Source data are available online for this figure.

© The Author(s)
