# Supplementary material for: Response of the replisome to araA and araC suggests a mechanism for arabinosyl nucleoside tolerance
Source: EMBO J. 2026 Aug 27;45(20):7191–225. doi: 10.1038/s44318-026-00901-1 (PMC13672804; doi:10.1038/s44318-026-00901-1)
Supplement: Supplementary file 2 [file 44318_2026_901_MOESM1_ESM.pdf]

Appendix for
Response of the replisome to araA and araC suggests a mechanism for arabinosyl nucleoside tolerance

Harry Orrin and Joseph T. P. Yeeles

Table of Contents

Appendix Figure S1	Page 2
Appendix Figure S2	Page 3
Appendix Figure S3	Page 4
Appendix Figure S4	Page 5
Appendix Figure S5	Pages 6-8
Appendix Table S1	Pages 9-13
Appendix Table S2	Pages 14-17
Appendix Table S3	Page 18
Appendix Table S4	Pages 19-21

Appendix Figures (S1-5)

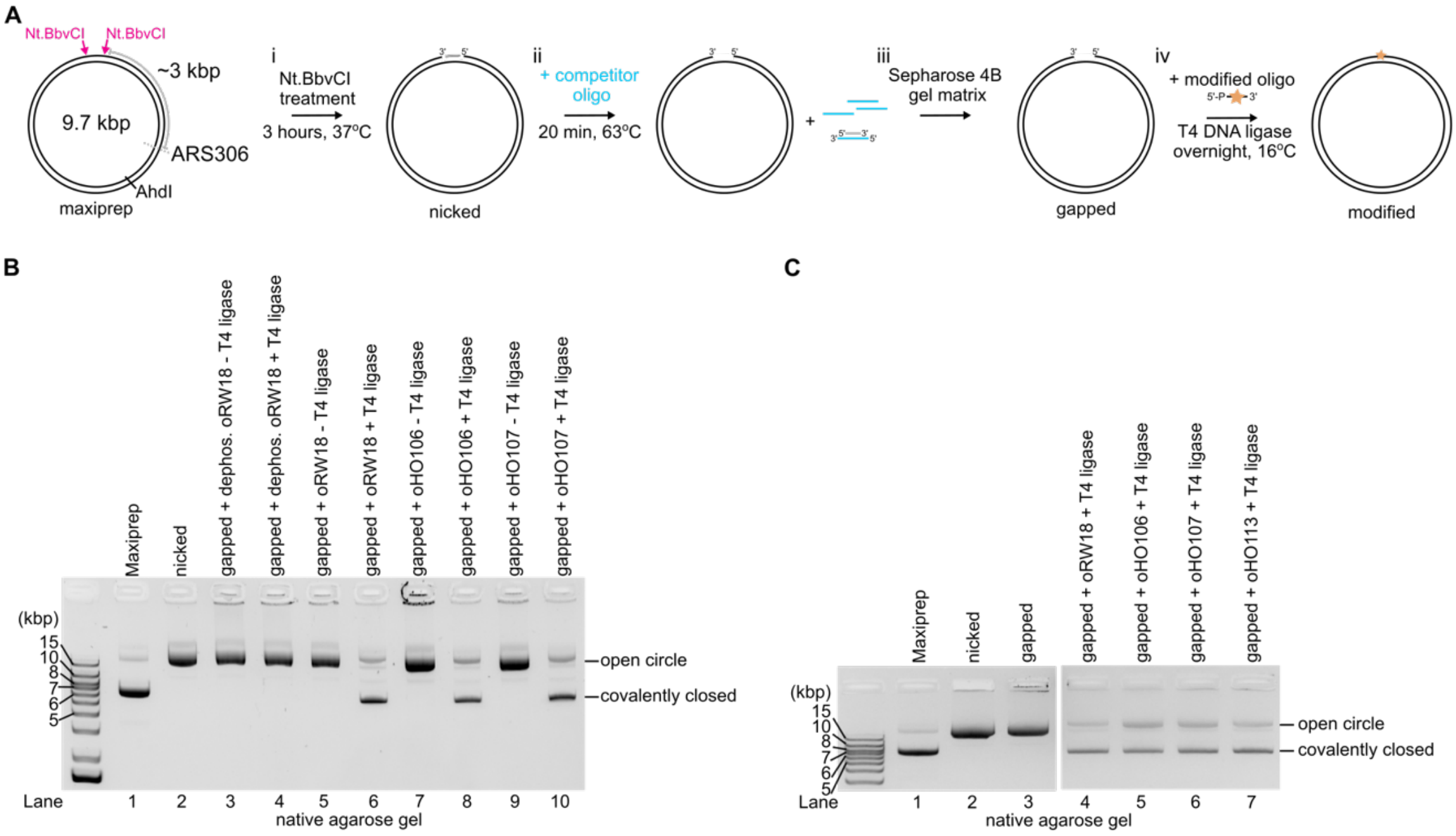

Appendix Figure S1. Generation of modified replication templates.

(A) Schematic illustrating the steps used to generate modified replication templates (for further details, see Methods).

(B) Native gel analysis of different steps of modified replication template preparation. Gel was stained with ethidium bromide which causes negative supercoiling and thus faster migration of circular, covalently-closed dsDNA. vRW31 isolation from Maxiprep kit (Lane 1). Post- Nt.BbvCI treatment (Lane 2). Gapped DNA incubated -/+ T4 DNA ligase with dephosphorylated oRW18 (Lanes 3-4); oRW18 (Lanes 5-6); oHO105 (Lanes 7-8); and oHO107 (Lanes 9-10).

(C) Independent replicates of template generation as in (B) but including gapped alone (Lane 3) and oHO113 + T4 DNA Ligase (Lane 7).

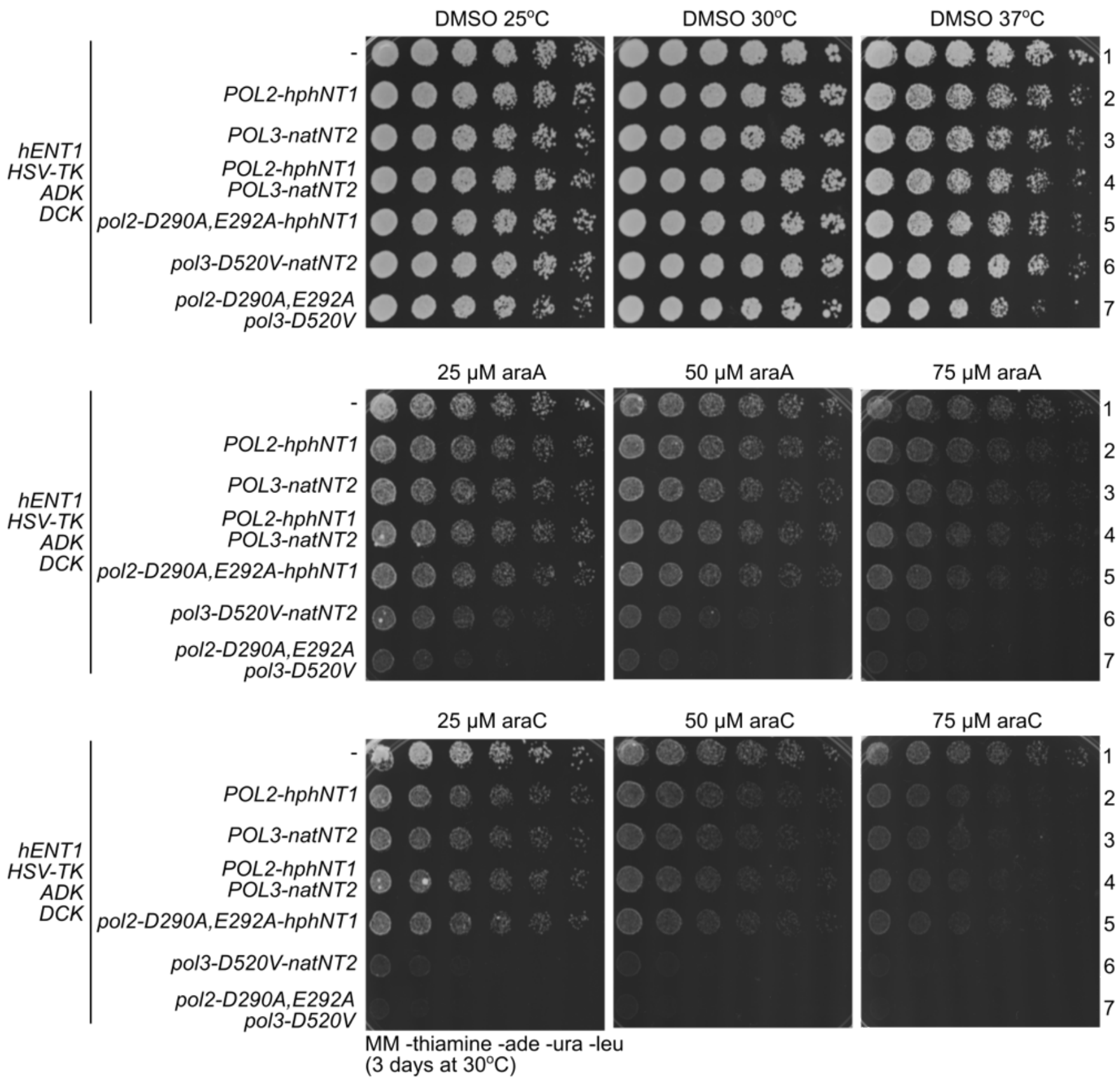

Appendix Figure S2. Marker insertion downstream of either *POL2* or *POL3* genes has no effect on cell growth.

Three-fold serial dilutions of the indicated genotypes (yHO25, yHO45, yHO56, yHO64, yHO43, yHO66, yHO62) spotted onto media with the indicated conditions.

From Figure 5A, 10 μM araA

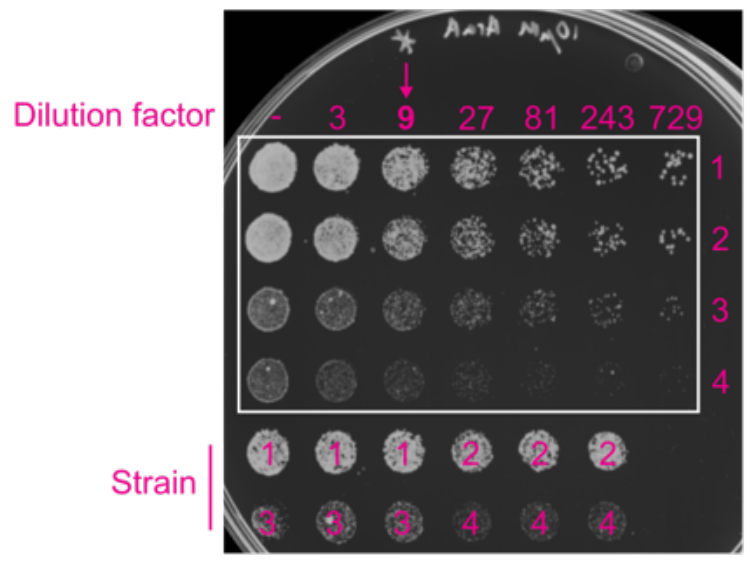

Appendix Figure S3. Quantification of growth assays.

Uncropped image of three-fold serial dilution from Figure 5A, 10 μM araA, as an example.

For each strain, 9-fold dilution (indicated) was spotted in triplicate beneath cropped gel image (white rectangle). These spots were quantified using FIJI approximately every 24 hours, averaged using Excel, and converted into a percentage relative to growth on DMSO after 5 days, as described in Methods. This was done a total of 3 times from three independent yeast cultures and plotted using Prism, where error bars represent standard deviation.

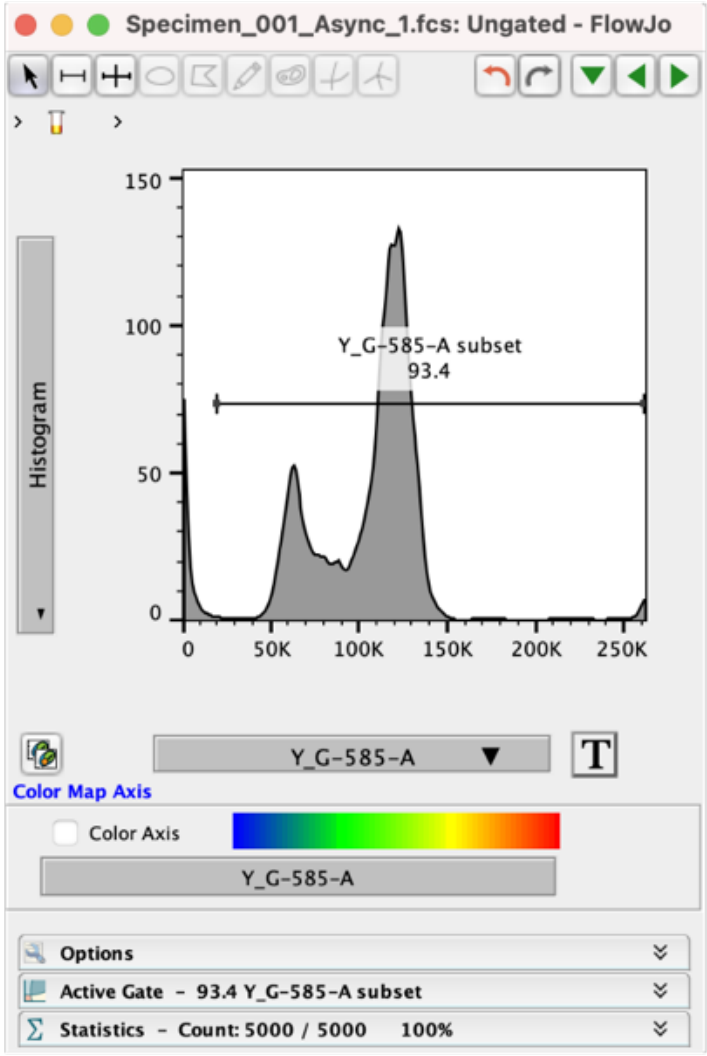

Appendix Figure S4. Gating strategy for flow cytometry analysis of DNA content.

An asynchronous sample histogram (Y-G-585-A vs Count) was manually gated as above in FlowJo and this was applied to all other samples in the same experiment.

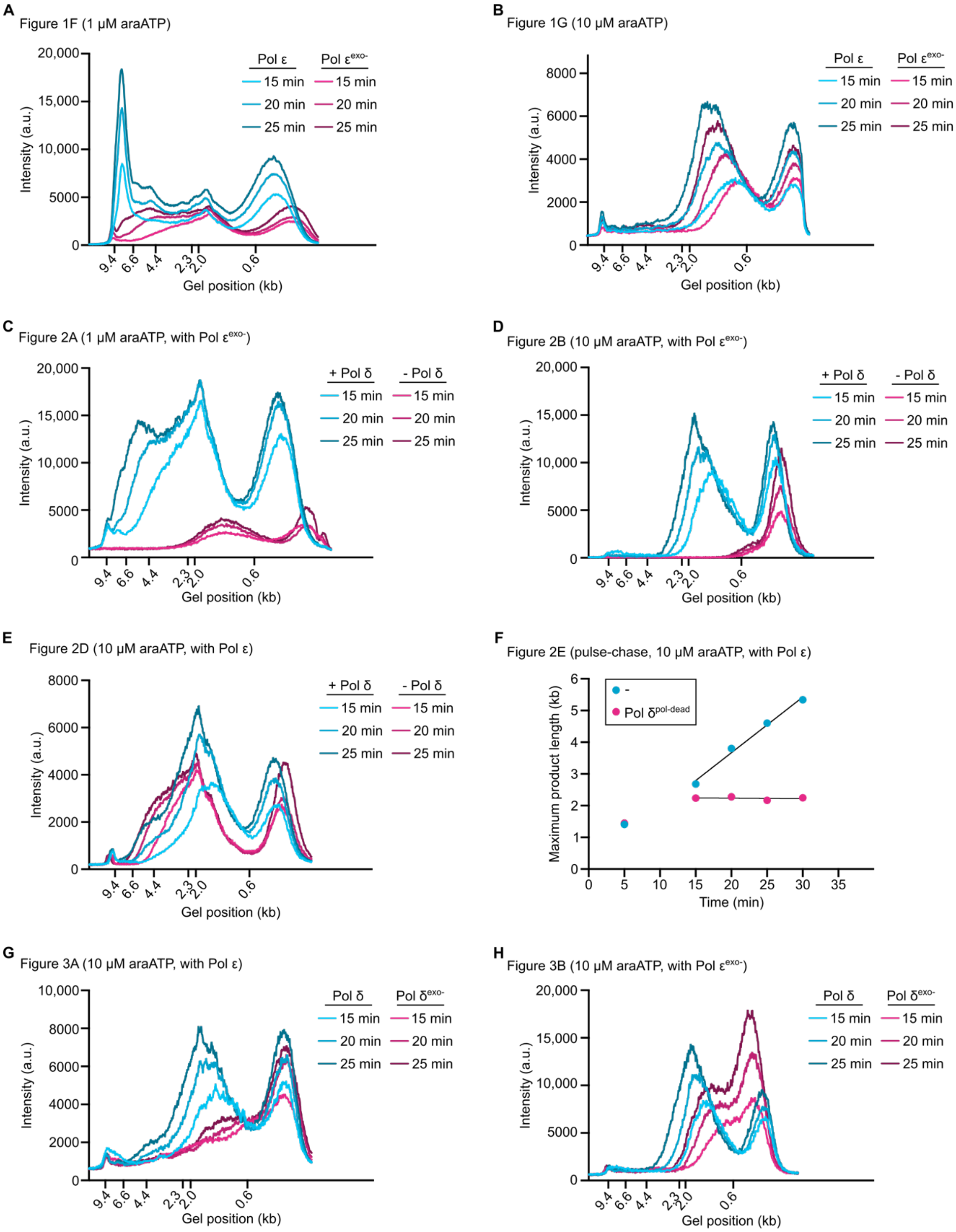
A
Figure 1F (1 µM araATP)
Pol ε
Pol ε exo-
15 min
20 min
25 min
Intensity (a.u.)
Gel position (kb)
B
Figure 1G (10 µM araATP)
C
Figure 2A (1 µM araATP, with Pol ε exo-)
+ Pol δ
- Pol δ
D
Figure 2B (10 µM araATP, with Pol ε exo-)
E
Figure 2D (10 µM araATP, with Pol ε)
F
Figure 2E (pulse-chase, 10 µM araATP, with Pol ε)
-
Pol δ pol-dead
Maximum product length (kb)
Time (min)
G
Figure 3A (10 µM araATP, with Pol ε)
Pol δ
Pol δ exo-
H
Figure 3B (10 µM araATP, with Pol ε exo-)

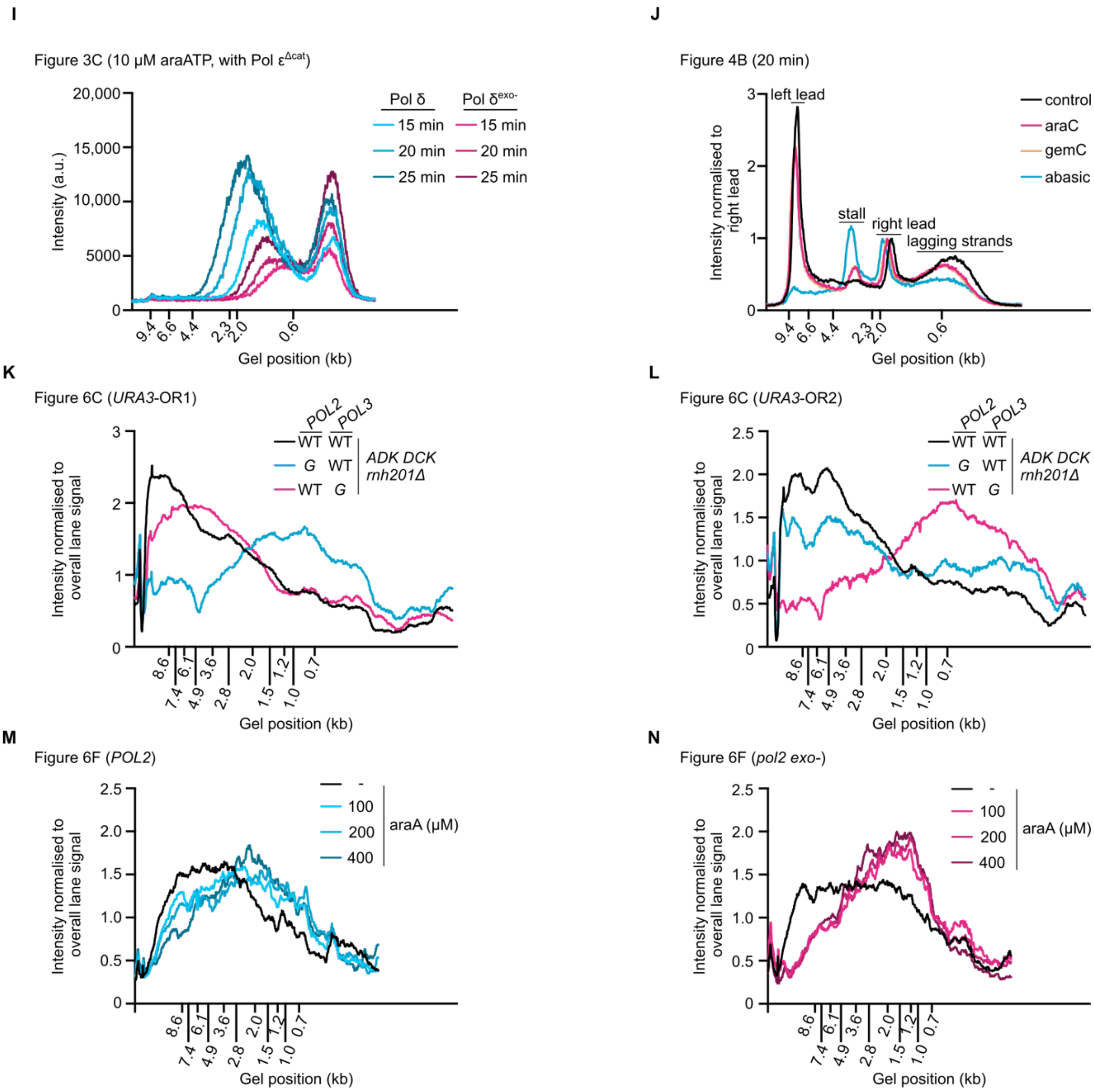

Appendix Figure S5. Quantification of indicated gels

(A) Intensity profiles of lanes 4-6 and 10-12 of gel presented in Figure 1F.

(B) Intensity profiles of lanes 7-12 of gel presented in Figure 1G.

(C) Intensity profiles of lanes 4-6 and 10-12 of gel presented in Figure 2A.

(D) Intensity profiles of lanes 4-6 and 10-12 of gel presented in Figure 2B.

(E) Intensity profiles of lanes 7-12 of gel presented in Figure 2D.

(F) Maximum synthesis rates of experiment presented in Figure 2E fit to a linear regression (see Methods).

(G) Intensity profiles of lanes 4-6 and 10-12 of gel presented in Figure 3A.

(H) Intensity profiles of lanes 4-6 and 10-12 of gel presented in Figure 3B.

(I) Intensity profiles of lanes 4-6 and 10-12 of gel presented in Figure 3C.

(J) Intensity profiles of lanes 2, 4, 6, and 8 of gel presented in Figure 4B normalised to the right lead product.

(K-L) Intensity profiles of either lanes 1-3 (K) or 4-6 (L) of blot presented in Figure 6C normalised to overall lane signal.

(M-N) Intensity profiles of either lanes 1-4 (M) or 5-8 (N) of blot presented in Figure 6F normalised to overall lane signal.

Appendix Table S1. Oligonucleotides used in this study

Identifier	Purpose	Sequence (5'-3')	Source
JY180	To singly prime M13mp18 ssDNA for primer extension reactions.	GAATAATGGAAGGGTTAGAACCTACCAT	(Yeeles *et al*, 2017)
oHO10	3' PCR. Site-directed mutagenesis of synthetic *POL3*-containing plasmid (pRS306/*POL3-pGAL-1-10- POL31*) to generate *pol3-D321A,E323A* allele.	TAGCGAAAGACATGATTCTCAATG	This study
oHO106	5' phosphorylated oligo used to generate a modified replication template containing a single internal araC base.	P-TCAGCAAAAAGA/araC/TTCGAAAAATTTTTCC	This study
oHO107	5' phosphorylated oligo used to generate a modified replication template containing a single internal gemC base.	P-TCAGCAAAAAGA/gemC/TTCGAAAAATTTTTCC	This study
oHO113	5' phosphorylated oligo used to generate a modified replication template containing a single internal 1',2'-dideoxyribose spacer (idSp) modification (artificial abasic site).	P-TCAGCAAAAAGA/idSp/TTCGAAAAATTTTTCC	This study
oHO114	5' PCR. Site-directed mutagenesis of W303 *S. cerevisiae POL2* gene to generate *pol2-M644G* allele.	TGTCGCCTCTGGATACCCAAACATC	This study
oHO115	3' PCR. Site-directed mutagenesis of W303 *S. cerevisiae POL2* gene to generate *pol2-M644G* allele.	TCTACATGATAGATCAAAGG	This study
oHO136	5' PCR. Generate *KanMX4* cassette to knock out W303 *S. cerevisiae ura3-1* cassette.	GATAAGTTTTGACCATCAAAGAAGGTTAATGTGGCTGTGGTTTCAGGGTCCATAAAGCTTCGGATCCCCGGGTTAATTAA	This study

oHO137	3' PCR. Generate *KanMX4* cassette to knock out W303 *S. cerevisiae ura3-1* cassette.	ATACTGTTACTTGGTTCTGGCGAGGTATTGGATAGTTCCTTTTTATAAAGGCCATGAAGCGAATTCGAGCTCGTTTAAAC	This study
oHO138	5' PCR. Amplify *URA3* cassette for integration near ARS306 on chr III in W303 *S. cerevisiae* in OR1.	CACAAAGTAGTGATGACCCACTCAGGTGTTAAAGTGTGTCATATCTGCAAATGACACTGTCTGTACAATACCAGTGACTG	This study
oHO139	3' PCR. Amplify URA3 cassette for integration near ARS306 on chr III in W303 *S. cerevisiae* in OR1.	GTAGTAATAGCATACGTGTAATGATAGACGATTTTCATTTTAATTCTAGTCCTTTATGACATCGATGAATTCGAGCTCG	This study
oHO140	5' PCR. Amplify *URA3* cassette for integration near ARS306 on chr III in W303 *S. cerevisiae* in OR2.	GTAGTAATAGCATACGTGTAATGATAGACGATTTTCATTTTAATTCTAGTCCTTTATGAGCTGTACAATACCAGTGACTG	This study
oHO141	3' PCR. Amplify URA3 cassette for integration near ARS306 on chr III in W303 *S. cerevisiae* in OR2.	CACAAAGTAGTGATGACCCACTCAGGTGTTAAAGTGTGTCATATCTGCAAATGACACTGTCATCGATGAATTCGAGCTCG	This study
oHO188	5' PCR. Site-directed mutagenesis of W303 *S. cerevisiae POL3* gene to generate *pol3-L612G* allele.	TTTCAATTCTGGATATCCAAGTATTATGATG	This study
oHO189	3' PCR. Site-directed mutagenesis of W303 *S. cerevisiae POL3* gene to generate *pol3-L612G* allele.	TCCAAAGTTGCAATCG	This study
oHO206	5' PCR. 5' phosphorylated primer used with oHO207 to generate PCR product for probing URA3 gene.	P-CAGTTAAGCCGCTAAAGGCA	This study
oHO207	3' PCR. 5' 5x phosphorothioated primer used with oHO206 to generate PCR product for probing URA3 gene.	C*C*G*C*T*AACAATACCTGGGCC	This study

oHO23	5' PCR. Site-directed mutagenesis of W303 *S. cerevisiae POL2* gene to generate *pol2-D290A,E292A* allele.	TAGCAACCACGAAGCCGCCTTTAAAATTC	This study
oHO24	3' PCR. Site-directed mutagenesis of W303 *S. cerevisiae POL2* gene to generate *pol2-D290A,E292A* allele.	TTGCAAATGCCATTACCACAGGGTC	This study
oHO25	5' PCR. Site-directed mutagenesis of W303 *S. cerevisiae POL3* gene to generate *pol3-D321A,E323A* allele.	TCGCATGTGCTGGTAGGATTG	This study
oHO26	3' PCR. Site-directed mutagenesis of W303 *S. cerevisiae POL3* gene to generate *pol3-D321A,E323A* allele.	TTGCAAAGGACATGATACGCAATG	This study
oHO39	5' PCR. Site-directed mutagenesis of W303 *S. cerevisiae POL3* gene to generate *pol3-D520V* allele.	CTGTTTGAAAGTGGCCTACCTGC	This study
oHO43	5' PCR. Amplify W303 *S. cerevisiae POL2-hphNT1* construct and its alleles to replace endogenous gene.	ATGATGTTTGGCAAGAAAAAAAAC	This study
oHO44	3' PCR. Contains 5' overhang homologous to 50 bp immediately after TGA stop codon. Amplify W303 *S. cerevisiae POL2-hphNT1* construct and its alleles to replace endogenous gene.	TTTTTTTCATGGTAAAGAGGCCATTGAACCTCGCGTTATATACTGCTTACTTAAAGCCTTCGAGCGTC	This study
oHO45	5' PCR. Amplify W303 *S. cerevisiae POL3-natNT2* construct and its alleles to replace endogenous gene.	ATGAGTGAAAAAAGATCCC	This study
oHO46	3' PCR. Contains 5' overhang homologous to 54 bp immediately	CAAAAAGTTGTTAGCCTTTCTTAATCCTAATATGATGTGCCACCCTATCGTTTTGAGCTCGATTACAACAGG	This study

	after TGA stop codon. Amplify W303 *S. cerevisiae POL3-natNT2* construct and its alleles to replace endogenous gene.		
oHO50	5' PCR. Site-directed mutagenesis of synthetic *POL2*-containing plasmid (pAJ6) to generate *pol2-P301R* allele.	ATTGAAGTTCAGAGACTCTGCTG	This study
oHO51	3' PCR. Site-directed mutagenesis of synthetic *POL2*-containing plasmid (pAJ6) to generate *pol2-P301R* allele.	GGTGGCTTAGTAGTTTC	This study
oHO52	5' PCR. Site-directed mutagenesis of W303 *S. cerevisiae POL2* gene to generate *pol2-P301R* allele.	TTTAAAATTCAGAGATTCCGCCGTAG ATCAAATAATGATG	This study
oHO53	3' PCR. Site-directed mutagenesis of W303 *S. cerevisiae POL2* gene to generate *pol2-P301R* allele.	GGCGGCTTCGTGGTT	This study
oHO54	3' PCR. Site-directed mutagenesis of W303 *S. cerevisiae POL3* gene to generate *pol3-D520V* allele.	TAAACGGCCAACCTTCTTC	This study
oHO9	5' PCR. Site-directed mutagenesis of synthetic *POL3*-containing plasmid (pRS306/*POL3-pGAL-1-10- POL31*) to generate *pol3-D321A,E323A* allele.	TCGCTTGTGCTGGTAGAATTGG	This study
oRW18	5' phosphorylated oligo used to generate a modified replication template containing a single deoxycytidine (control).	P-TCAGCAAAAAGACTTCGAAAAATTTTTCC	(Westhorpe *et al*, 2024)
oRW25	Competitor oligo used to generate modified replication templates.	GTTGGTACTGCGCTGAGGAAA AATTTTTCGAAGTCTTTTTGCT GAGGTTGGTACTGCG	(Westhorpe *et al*, 2024)

PZ2836	5’ PCR. Generate *hphMX6* cassette to knock out W303 *S. cerevisiae RNH201.*	CTAATGAGAGTGTCGAAAAACCTTGAAAACAACTACTGCACACCAAATTGATACGATTAACGGATCCCCGGGTTAATTAA	This study
PZ2837	3’ PCR. Generate *hphMX6* cassette to knock out W303 *S. cerevisiae RNH201.*	TGAAGTTATGACATATGTAGTATTACATGAAGATATATAGTATGTGCAAACTGGAGGTGAGAATTCGAGCTCGTTTAAAC	This study

Appendix Table S2. Details of proteins used in this study

Protein	Affinity tag	Purified as described in	Purification steps	Final buffer
Cdc45	Internal 2xFLAG tag	(Yeeles *et al*, 2015)	1. Anti-FLAG M2 Agarose<br>2. Bio-Gel HT Hydroxyapatite	25 mM HEPES-KOH, pH 7.6<br>10% glycerol<br>1 mM EDTA<br>1 mM DTT<br>300mM KOAc
Cdc6	N-terminal cleavable GST tag	(Frigola *et al*, 2013)	1.Glutathione Sepharose 4B<br>2.Bio-Gel HT Hydroxyapatite	50 mM K_2HPO_4/KH_2PO_4, pH 7.5<br>5 mM $MgCl_2$<br>1% Triton X-100<br>1 mM DTT<br>0.4 M KOAc<br>15% glycerol
Cdt1.Mcm2-7	N-terminal cleavable CBP tag on Mcm3	(Coster *et al*, 2014)	1. Calmodulin Sepharose 4B<br>2. Superdex 200	45 mM HEPES-KOH, pH 7.6<br>100 mM KOAc<br>5 mM MgOAc<br>0.02% NP40<br>10% glycerol
Csm3/Tof1	Cleavable Nterminal CBP tag on Csm3	(Baretić *et al*, 2020)	1. Calmodulin Sepharose 5B<br>2. TEV cleavage/elution<br>3. MonoQ<br>4. Superdex 200	25 mM Tris-HCl pH 7.2<br>10 % glycerol<br>0.02% NP-40-S<br>1mM DTT<br>150 mM NaCl
Ctf18-RFC	N-terminal CBP tag on Rfc3	(Fletcher *et al*, 2025)	1. Calmodulin Sepharose 4B<br>2. MonoS<br>3. Superdex 200	25 mM HEPES-KOH pH 7.6<br>10% glycerol<br>1 mM DTT<br>1 mM EDTA<br>300 mM NaCl
Ctf4	N-terminal CBP tag	(Yeeles *et al*, 2015)	1. Calmodulin Sepharose 4B<br>2. MonoQ<br>3. Superdex 200	25 mM Tris-HCl pH 7.2<br>10% glycerol<br>1 mM DTT<br>75 mM NaCl<br>1 mM EDTA
DDK	CBP tag on Dbf4	(On *et al*, 2014)	1. Calmodulin Sepharose 4B<br>2. Lambda phosphatase dephosphorylation<br>3. Superdex 200	25 mM HEPES-KOH, pH 7.6<br>0.1 mM EGTA<br>0.1 mM EDTA<br>0.02% NP40<br>10% glycerol

				100 mM KGlu<br>2 mM β-mercaptoethanol
Dpb11	C-terminal 3xFLAG tag	(Yeeles *et al*, 2015)	1. Anti-FLAG M2 Agarose<br>2. MonoS	25 mM HEPES-KOH, pH 7.6 10% glycerol<br>0.02% NP-40-S<br><br>1 mM EDTA<br>1 mM DTT<br>300mM KOAc
GINS	N-terminal His tag on Psf3	(Yeeles *et al*, 2015)	1. Ni-NTA Agarose<br>2. MonoQ<br>3. Superdex 200	25 mM HEPES-KOH, pH 7.6<br>10% glycerol<br>0.02% NP-40-S<br>1 mM EDTA<br>1 mM DTT<br>200mM KOAc
Mcm10	N-terminal His tag	(Yeeles *et al*, 2015)	1. Ni-NTA Agarose<br>2. MonoS (2x)	25 mM HEPES-KOH, pH 7.6<br>10% glycerol<br>0.01% NP-40-S<br>1 mM EDTA<br>1 mM DTT<br>200 mM KGlu
Mrc1	C-terminal 2xFLAG tag	(Baretić *et al*, 2020)	1. Anti-FLAG M2 Agarose<br>2. Superose 6	25 mM Tris-HCl pH 7.2<br>10% glycerol<br>0.02% NP-40-S<br>1mM DTT<br>150 mM NaCl
ORC	Cleavable CBP tag on Orc1	(Frigola *et al*, 2013)	1.Calmodulin Sepharose 4B<br>2. Superdex 200	25 mM HEPES-KOH pH 7.6,<br>0.05% NP-40-S 10% glycerol<br>300 mM KOAc
PCNA	Untagged	(Yeeles *et al*, 2017)	1. Nucleic acid precipitation with Polymin P<br>2. Ammonium sulfate precipitation<br>3. HiTrap SP HP (flow through)<br>4. HiTrap Heparin HP (flow through)<br>5. HiTrap DEAE Fast	25 mM Tris-HCl, pH 7.2<br>10% glycerol<br>1 mM EDTA<br>150 mM NaCl

			Flow<br>6. MonoQ<br>7. Superdex 200	
Pol ε	C-terminal CBP tag on Dpb4	(Yeeles *et al*, 2015)	1. Calmodulin Sepharose 4B<br>2. HiTrap Heparin HP<br>3. Superdex 200	25 mM HEPES-KOH, pH 7.6<br>10% glycerol<br>1 mM DTT<br>500 mM KOAc
Pol δ	C-terminal CBP tag on Pol32	(Yeeles *et al*, 2017)<br>Note different final buffer	1. Calmodulin Sepharose 4B<br>2. HiTrap Heparin HP<br>3. Superdex 200	25 mM Tris-HCl pH 7.2<br>10% glycerol<br>0.02% NP-40-S<br>1 mM EDTA<br>1 mM DTT<br>250 mM NaCl
Pol α-primase	N-terminal CBP tag on Pri1	(Yeeles *et al*, 2015)	1. Calmodulin Sepharose 4B<br>2. MonoQ<br>3. Superdex 200	25mM HEPES-KOH pH 7.6<br>10% glycerol<br>400 mM KOAc<br>0.02% NP-40-S<br>1mM DTT<br>1 mM EDTA
Pol ε^{exo-}	C-terminal CBP tag on Dpb4. Endogenous Pol2 3X FLAG-tagged	(Guilliam & Yeeles, 2021)	1. Calmodulin Sepharose 4B<br>2. Anti-FLAG M2 Agarose (flow through)<br>2. HiTrap Heparin HP<br>3. Superdex 200	25 mM HEPES-KOH, pH 7.6<br>10% glycerol<br>1 mM DTT<br>500 mM KOAc
Pol δ^{exo-}	C-terminal CBP tag on Pol32	This study	1. Calmodulin Sepharose 4B<br>2. HiTrap Heparin HP<br>3. Superdex 200	25 mM Tris-HCl pH 7.2<br>10% glycerol<br>0.02% NP-40-S<br>1 mM EDTA<br>1 mM DTT<br>250 mM NaCl
Pol ε^{P301R}	C-terminal CBP tag on Dpb4. Endogenous Pol2 3X FLAG-tagged	This study	1. Calmodulin Sepharose 4B<br>2. Anti-FLAG M2 Agarose (flow through)<br>2. HiTrap Heparin HP<br>3. Superdex 200	25 mM HEPES-KOH, pH 7.6<br>10% glycerol<br>1 mM DTT<br>500 mM KOAc
Pol $\delta^{POL-DEAD}$	C-terminal CBP tag on Pol32	(Aria & Yeeles, 2019)	1. Nucleic acid precipitation with Polymin P<br>2. Ammonium sulfate precipitation<br>3. Calmodulin Sepharose 4B<br>4. MonoQ	25 mM HEPES-KOH, pH 7.6<br>40% glycerol<br>300 mM KOAc<br>0.02% NP-40-S<br>1 mM DTT

Pol $\varepsilon^{\Delta CAT}$	C-terminal CBP tag on Dpb4	(Yeeles *et al*, 2017)	1. Calmodulin Sepharose 4B<br>2. MonoQ<br>3. Superdex 200	25 mM HEPES-KOH, pH 7.6<br>10% glycerol<br>1 mM DTT<br>500 mM KOAc
RFC	N-terminal CBP tag on Rfc3	(Yeeles *et al*, 2017)	1. Calmodulin Sepharose 4B<br>2. MonoS<br>3. Superdex 200	25 mM HEPES-KOH pH 7.6<br>10% glycerol<br>1 mM DTT<br>1 mM EDTA<br>150 mM NaCl
RPA	Untagged	(Baretić *et al*, 2020)	1. Nucleic acid precipitation with Polymin P<br>2. Ammonium sulfate precipitation<br>3. HiTrap Blue HP<br>4. ssDNA Cellulose<br>5. MonoQ	25 mM Tris-HCl, pH 7.5<br>1 mM EDTA<br>10% glycerol<br>100 mM NaCl<br>1 mM DTT
S-CDK (truncated Clb5, Δ1-100)	Cleavable CPB tag on Clb5	(Yeeles *et al*, 2015)	1. Calmodulin Sepharose 4B<br>2. TEV cleavage (elution)<br>3. Superose 6	40 mM HEPES-KOH, pH 7.6<br>10% glycerol,<br>0.02% NP-40-S<br>300 mM KOAc
Sld2	C-terminal 3xFLAG tag	(Yeeles *et al*, 2015)	1. Ammonium sulfate precipitation<br>2. Anti-FLAG M2 Agarose<br>3. HiTrap SP HP	25 mM HEPES-KOH, pH 7.6<br>0.02% NP-40-S,<br>1 mM EDTA<br>1 mM DTT<br>40% glycerol<br>350 mM KCl
Sld3/7	Cleavable C-terminal TCP tag	(Yeeles *et al*, 2015)	1. IgG Sepharose Fast Flow<br>2. TEV removal with Ni-NTA Agarose<br>3. Superdex 200	25 mM HEPES-KOH, pH 7.6<br>10% glycerol,<br>0.02% NP-40-S<br>1 mM EDTA<br>1 mM DTT<br>500 mM KCl
Top1	Cleavable N-terminal CBP tag	(Yeeles *et al*, 2015)	1. Calmodulin Sepharose 4B<br>2. TEV removal with Talon column<br>3. Superdex 200	25 mM HEPES-KOH, pH 7.6<br>10% glycerol<br>400 mM KOAc<br>0.02% NP-40-S<br>1mM DTT

Appendix Table S3. Details of budding yeast media used in this study

Medium	Composition
YEPD<br>(Yeast Extract, Peptone, Dextrose)	1.1% (w/v) yeast extract<br>2.2% (w/v) peptone<br>0.0055% (w/v) adenine sulphate<br>2% (w/v) glucose
Minimal agar plates -thiamine<br>(auxotrophic markers omitted as indicated)	0.7% (w/v) yeast nitrogen base without amino acids and without thiamine<br>0.08 mg/mL leucine<br>0.04 mg/mL methionine, adenine sulphate, tryptophan, histidine, uracil<br>2% (w/v) glucose<br>2% (w/v) agar
S mix -thiamine	68.3% (w/w) ammonium sulphate<br>23% (w/w) yeast nitrogen base without amino acids, without ammonium sulphate, without thiamine<br>7% (w/w) potassium phosphate dibasic<br>0.23% (w/w) aspartic acid<br>0.23% (w/w) threonine<br>0.23% (w/w) valine<br>0.18% (w/w) phenylalanine<br>0.1% (w/w) isoleucine<br>0.1% (w/w) lysine<br>0.1% (w/w) tyrosine<br>0.07% (w/w) arginine<br>0.07% (w/w) cysteine<br>0.07% (w/w) proline<br>0.07% (w/w) serine
SC -thiamine<br>(synthetic complete, auxotrophic markers omitted as indicated)	0.7% (w/w) S mix -thiamine<br>0.08 mg/mL leucine<br>0.04 mg/mL methionine, adenine sulphate, tryptophan, histidine, uracil<br>2% (v/v) glucose

Appendix Table S4. Reports of one-way ANOVAs and, where appropriate, Tukey's multiple comparisons tests (related to Figures 5B and EV5E, 5-day timepoint)

ANOVA, analysis of variance. SS, sum of squares. DF, degrees of freedom. MS, mean square. SE, standard error. Diff., difference. CI, confidence interval. Ns = not significant ($p > 0.05$); * = $p \leq 0.05$; ** = $p \leq 0.01$; *** = $p \leq 0.001$; **** = $p \leq 0.0001$.

**Figure 5B, DMSO**					
**ANOVA**	**SS**	**DF**	**MS**	**F (DFn, DFd)**	**P value**
Treatment (between columns)	39.41	3	13.14	F (3, 8) = 0.9382	P=0.4661
Residual (within columns)	112.0	8	14.00		

**Figure 5B, 10 μM araA**									
**ANOVA**		**SS**		**DF**	**MS**	**F (DFn, DFd)**		**P value**	
Treatment (between columns)		14087		3	4696	F (3, 8) = 144.8		P<0.0001	
Residual (within columns)		259.5		8	32.44				
**Tukey's multiple comparisons test**									
**1**	**2**	**Mean 1**	**Mean 2**	**Mean diff.**	**SE of diff.**	**95% CI of diff.**	**Below threshold?**	**Summary**	**Adjusted P value**
WT	*pol2 exo-*	102.9	102.8	0.1300	4.650	-14.76 to 15.02	No	ns	0.99999145
WT	*pol3 5DV*	102.9	56.86	46.02	4.650	31.12 to 60.91	Yes	****	0.00004277
WT	*pol2 exo- pol3 5DV*	102.9	21.29	81.59	4.650	66.70 to 96.49	Yes	****	0.00000053
pol2 exo-	*pol3 5DV*	102.8	56.86	45.89	4.650	30.99 to 60.78	Yes	****	0.00004368
pol2 exo-	*pol2 exo- pol3 5DV*	102.8	21.29	81.46	4.650	66.57 to 96.36	Yes	****	0.00000054
pol3 5DV	*pol2 exo- pol3 5DV*	56.86	21.29	35.58	4.650	20.68 to 50.47	Yes	***	0.00027634

Figure 5B, 10 μM araC					
**ANOVA**	**SS**	**DF**	**MS**	**F (DFn, DFd)**	**P value**
Treatment (between columns)	22949	3	7650	F (3, 8) = 321.5	P<0.0001
Residual (within columns)	190.3	8	23.79		

**Tukey's multiple comparisons test**									
**1**	**2**	**Mean 1**	**Mean 2**	**Mean diff.**	**SE of diff.**	**95% CI of diff.**	**Below threshold?**	**Summary**	**Adjusted P value**
WT	*pol2 exo-*	100.4	101.5	-1.137	3.983	-13.89 to 11.62	No	ns	0.99126512
WT	*pol3 5DV*	100.4	21.51	78.89	3.983	66.13 to 91.64	Yes	****	0.00000018
WT	*pol2 exo- pol3 5DV*	100.4	6.757	93.64	3.983	80.89 to 106.4	Yes	****	0.00000004
pol2 exo-	*pol3 5DV*	101.5	21.51	80.02	3.983	67.27 to 92.78	Yes	****	0.00000016
pol2 exo-	*pol2 exo- pol3 5DV*	101.5	6.757	94.78	3.983	82.03 to 107.5	Yes	****	0.00000004
pol3 5DV	*pol2 exo- pol3 5DV*	21.51	6.757	14.76	3.983	2.003 to 27.51	Yes	*	0.02483662

Figure 5B, 5 μM gemC					
**ANOVA**	**SS**	**DF**	**MS**	**F (DFn, DFd)**	**P value**
Treatment (between columns)	236.1	3	78.71	F (3, 8) = 2.648	P=0.1205
Residual (within columns)	237.8	8	29.73		

**Figure EV5E, 20 μM araA**					
**ANOVA**	**SS**	**DF**	**MS**	**F (DFn, DFd)**	**P value**
Treatment (between columns)	13474	3	4491	F (3, 8) = 267.6	P<0.0001
Residual (within columns)	134.3	8	16.79		

**Tukey's multiple comparisons test**									
**1**	**2**	**Mean 1**	**Mean 2**	**Mean diff.**	**SE of diff.**	**95% CI of diff.**	**Below threshold?**	**Summary**	**Adjusted P value**
WT	*pol2 exo-*	90.72	71.28	19.44	3.345	8.727 to 30.15	Yes	**	0.00179279
WT	*pol3 5DV*	90.72	25.43	65.30	3.345	54.58 to 76.01	Yes	****	0.00000020
WT	*pol2 exo- pol3 5DV*	90.72	7.787	82.94	3.345	72.22 to 93.65	Yes	****	0.00000003
pol2 exo-	*pol3 5DV*	71.28	25.43	45.86	3.345	35.14 to 56.57	Yes	****	0.00000369
pol2 exo-	*pol2 exo- pol3 5DV*	71.28	7.787	63.50	3.345	52.78 to 74.21	Yes	****	0.00000026
pol3 5DV	*pol2 exo- pol3 5DV*	25.43	7.787	17.64	3.345	6.927 to 28.35	Yes	**	0.00333107

**Figure EV5E, 1 μM gemC**					
**ANOVA**	**SS**	**DF**	**MS**	**F (DFn, DFd)**	**P value**
Treatment (between columns)	40.04	3	13.35	F (3, 8) = 2.839	P=0.1058
Residual (within columns)	37.60	8	4.700		
