# Supplementary material for: Response of the replisome to araA and araC suggests a mechanism for arabinosyl nucleoside tolerance
Source: EMBO J. 2026 Aug 27;45(20):7191–225. doi: 10.1038/s44318-026-00901-1 (PMC13672804; doi:10.1038/s44318-026-00901-1)
Supplement: Supplementary file 3 [file 44318_2026_901_MOESM2_ESM.pdf]

THE EMBO JOURNAL

Response of the replisome to araA and araC suggests a mechanism for arabinosyl nucleoside tolerance

Harry Orrin and Joseph Yeeles

Corresponding author(s): Joseph Yeeles (jyeeles@mrclmb.ac.uk) , Harry Orrin (horrin@mrclmb.ac.uk)

Review Timeline:

Submission Date:	24th Apr 26
Editorial Decision:	29th May 26
Revision Received:	3rd Jul 26
Editorial Decision:	27th Jul 26
Revision Received:	31st Jul 26
Accepted:	4th Aug 26

Editor: Hartmut Vodermaier

Transaction Report:

(Note: With the exception of the correction of typographical or spelling errors that could be a source of ambiguity, letters and reports are not edited. Depending on transfer agreements, referee reports obtained elsewhere may or may not be included in this compilation. Referee reports are anonymous unless the Referee chooses to sign their reports.)

1st Editorial Decision 29th May 2026

Dr. Joseph TP Yeeles
MRC Laboratory of Molecular Biology (LMB)
Protein and Nucleic Acids Chemistry
Francis Crick Avenue
Cambridge CB2 0QH
United Kingdom

29th May 2026

Re: EMBOJ-2026-124467
How the eukaryotic replisome tolerates nucleoside analogue chemotherapies

Dear Joe,

Thank you for submitting your manuscript on nucleoside analogue tolerance by eukaryotic replisomes for our consideration. I sent it to three expert referees, who have now returned the reports copied below. As you will see, the referees appreciate the interest/importance of the topic and the overall quality of the work, but also raise some concerns with specific experiments and with a certain level of overinterpretation; especially referee 3 notes that not all of the results can be considered fully unexpected based on previous work.

Having further discussed these reports, we on balance concluded that we would be open to considering a revised version further for EMBO Journal publication. It will however be important to satisfactorily address the various technical points, in particular regarding drug concentrations, as well as to more carefully interpret the implications and findings, and to better discuss them in the context of existing knowledge on the ability of Pol delta to proofread errors made by Pol epsilon (and whether this might involve intrinsic and/or extrinsic proofreading).

Since we generally only allow a single major-revision round, please do not hesitate to contact me already at an early stage in case you would like to discuss options for best addressing key points of the referees. We also remain open to extending the regular three-months revision period if needed; our 'scooping protection' (meaning that competing work appearing elsewhere in the meantime will not affect our considerations of your study) would of course remain valid also throughout such an extension.

Further information on preparing, formatting and uploading a revised manuscript can be found below and in our Guide to Authors. Thank you again for the opportunity to consider this work for The EMBO Journal.

With kind regards,

Hartmut

Hartmut Vodermaier, PhD
Senior Editor, The EMBO Journal
h.vodermaier@embojournal.org

*** PLEASE NOTE: All revised manuscript are subject to initial checks for completeness and adherence to our formatting guidelines. Revisions may be returned to the authors and delayed in their editorial re-evaluation if they fail to comply to the following requirements. As a first step please read our guidelines for revised submissions:
https://link.springer.com/journal/44318/submission-guidelines#cms-Revised-submissions

1) Every manuscript requires a Data Availability section (even if only stating that no deposited datasets are included). Primary datasets or computer code produced in the current study have to be deposited in appropriate public repositories prior to resubmission, and reviewer access details provided in case that public access is not yet allowed.

2) Each figure legend must specify
- size of the scale bars that are mandatory for all micrograph panels
- the statistical test used to generate error bars and P-values
- the type error bars (e.g., S.E.M., S.D.)
- the number (n) and nature (biological or technical replicate) of independent experiments underlying each data point
- Figures may not include error bars for experiments with n<3; scatter plots showing individual data points should be used instead.

3) Revised manuscript text (including main tables, and figure legends for main and EV figures) has to be submitted as editable text file (e.g., .docx format). We encourage highlighting of changes (e.g., via text color) for the referees' reference.

4) Each main and each Expanded View (EV) figure should be uploaded as individual production-quality files (preferably in .eps, .tif, .jpg formats). For suggestions on figure preparation/layout, please refer to our Figure Preparation Guidelines: https://media.springernature.com/original/springer-cms/rest/v1/content/27825798/data/v1

5) Point-by-point response letters should include the original referee comments in full together with your detailed responses to them (and to specific editor requests if applicable), and also be uploaded as editable (e.g., .docx) text files.

6) Please complete our Author Checklist, and make sure that information entered into the checklist is also reflected in the manuscript; the checklist will be available to readers as part of the Review Process File.

7) All authors listed as (co-)corresponding need to deposit, in their respective author profiles in our submission system, a unique ORCiD identifier linked to their name. Please see our Guide to Authors for detailed instructions.

8) Please note that supplementary information at EMBO Press has been superseded by the 'Expanded View' for inclusion of additional figures, tables, movies or datasets; with up to five EV Figures being typeset and directly accessible in the HTML version of the article.

9) To facilitate reproducibilty and cross-laboratory adoption of methodologies, please structure the Materials & Methods section as outlined in our guide to authors, including a completed Reagents and Tools Table.

10) Digital image enhancement is acceptable practice, as long as it accurately represents the original data and conforms to community standards. If a figure has been subjected to significant electronic manipulation, this must be clearly noted in the figure legend and/or the 'Materials and Methods' section. The editors reserve the right to request original versions of figures and the original images that were used to assemble the figure. Finally, we generally encourage uploading of numerical as well as gel/blot image source data.

At EMBO Press we ask authors to provide source data for the main manuscript figures. You will receive a separate email with instructions for providing source data with your revised manuscript, including how to upload and organize the files.

In the interest of ensuring the conceptual advance provided by the work, we recommend submitting a revision within 3 months (27th Aug 2026). Please discuss the revision progress ahead of this time with the editor if you require more time to complete the revisions. Use the link below to submit your revision:

Link Not Available

Referee #1:

The manuscript by Orrin and Yeeles reports a detailed analysis of how the yeast replisome handles arabinosyl (ara) nucleoside analogues that are commonly used as chemotherapeutic agents in humans. The authors show that DNA polymerase delta plays a major role in sustaining replication in the presence of ara analogues, taking over DNA synthesis and proofreading on the leading strand, and limiting polymerase-helicase uncoupling, with these functions becoming more prominent when DNA polymerase epsilon proofreading is defective. The work uses a sophisticated arsenal of tools, including the complete replisome reconstituted in vitro and a genetic system allowing DNA polymerase assignment to leading and lagging strands. These approaches are almost too good for the question at hand, as the significance of a detailed understanding of how yeast proteins interact with specific human drugs is uncertain. Nevertheless, the study is of high quality and reveals the dynamics of replication machinery when challenged with chain-terminating nucleoside analogues. The following revisions would strengthen the conclusions and improve clarity.

Major comments:

1) Please provide quantification of relevant endpoints and statistical analysis for all gels, and also include indicators of statistical significance in the drug sensitivity assays.

2) All nucleoside incorporation experiments were done with araATP, but studies of template-embedded analogues only included araC and gemC. Please discuss this discrepancy and how it affects the overall conclusions.

3) Experimental details are not always explained clearly. The study alternates between using circular and linear DNA substrates,

providing no rationale for the substrate choice and sometimes confusing them (see minor comments for examples). I suggest moving Figure EV1D to the main text. Figure panels also need more detailed titles and legends.

4) The model in Figure 7 is overreaching. It proposes a very specific scenario not directly supported by the data. There does not seem to be evidence that ara nucleotides specifically affect the handover to pol epsilon at origins. Why cannot they affect pol epsilon processivity during elongation? It would be better to end with a more general model that this study directly supports and discuss different hypotheses for how pol delta participation in leading strand synthesis could be enhanced.

5) The discussion of implications for cancer therapy (p. 15-16) lacks depth. The various statements in this paragraph appear disconnected. Ultimately, it remains unclear how the findings in this work could help improve therapy and for which cancers.

Minor comments:

6) P. 3, ln. 71: Change "in this manuscript" to "in this study" or similar.
7) P. 4, ln. 101: Delete "corresponding" unless supported by quantification (the increase in theta intermediates seems very small compared to the loss of LRIs).
8) P. 5, ln. 130: "under these conditions" seems incorrect: unlike Fig. 1E, Fig. EV1E shows replication of the circular template.
9) P. 6, ln. 162-163: The conclusion sounds premature. The experiments described in the following paragraphs, indeed, provide evidence for pol delta synthesis on the leading strand, but this paragraph only describes inhibition of synthesis by the catalytically dead pol delta. I suggest rephrasing the conclusion.
10) P. 6, ln. 172: "we repeated the experiment" is incorrect (again, different assays were used in Fig. 2C and Fig. EV2D).
11) P. 9, ln. 260-261: "began to sensitize cells at higher concentrations" - the difference seems to be in the assays, not concentrations. Figure EV5D shows no effect at 40 uM concentration.
12) P. 29, ln. 520-524: "Figures were performed" should read "Assays/experiments/reactions in figures were performed".
13) Materials and Methods subtitles "Budding yeast replication reactions" are misleading, as the reactions are with purified proteins rather than yeast.
14) Figures 2A and EV2A-B: indicate which pol epsilon variant was used.
15) Figure 4B-C: indicate that the reaction products were digested with SmaI.
16) Figure EV3A legend: "as in Figure 1C" - the DNA template in Figure 1C is circular.
17) Figure EV4 legend is confusing.

Referee #2:

This manuscript focuses on the DNA replication of nucleotide analogues (NAs) and employs both an in vitro assay using a reconstituted replication system and in vivo assays, including measurements of polymerase usage. This is an extensive study that addresses changes in DNA polymerase functions in response to NAs and provides valuable case studies illustrating how replication forks and their components can be flexible. In my opinion, while the overall structure of this study is sound and well described, I have several comments that may help further improve the manuscript.

Figure 1:
At 10 μM, araATP causes a clearly more severe perturbation of both leading and lagging strand synthesis. I wonder whether the authors can exclude the possibility that this strong effect is due to inhibition of DNA unwinding by the MCM helicase.

Figure 2:
While the data support the conclusions, the explanation of the experiments is confusing, taking several reads to understand the data presented. We suggest this could be simplified by presenting the information in an alternative order, presenting Figure 2B, 2C, then 2A. This would establish the effect of Pol delta omission first, then move to the confirmatory pulse-chase experiment, which is relatively complicated. An interpretation should be provided for the lack of difference observed with WT Pol epsilon in Figures 2B and 2C, in contrast to Figure 2A, and also why exo- gives different results.

Figure 3:
The description of results in the "Pol δ proofreads the leading strand..." section is not clearly matched to what the section title and Figure 3 aim to show. A description of how the assay/results demonstrate leading strand proofreading should be provided to aid the reader.

Figure 6:
This figure provides strong in vivo evidence for the involvement of Pol delta in leading-strand DNA synthesis. To show that DNA fragmentation results from cleavage at ribonucleotides, rather than from the nature of the pol2-exo- cells, araA treatment, or their combination, I suggest that the authors explain this part more carefully using the control experiments. For example, they could directly compare "POL3-WT pol2-exo- + araA" in Figure EV6E, lane 14, with "pol3-L612G pol2-exo- + araA" in Figure 6F, lane 8.

Minor:
In lines 177-180, I suggest that the authors avoid discussing differences between samples from different experimental batches.

There appears to be a dominant negative effect in Figure 3B, where having Pol deltaexo- is worse than omitting Pol dleta (Figure 2B). Could the authors explain the cause of this in the text? Is this due to different experiment baches?

Please provide the amino acid changes in the Pol epsilon exo- mutant when first mentioned.

In lines 404-406, the authors suggest a role for CTF18-RFC in minimizing the uncoupling of leading-strand synthesis. However, in Figure 1D, they did not observe a clear effect upon addition of this factor. I wonder how the authors reconcile these two parts.

Referee #3:

The aim of this manuscript is to explain how the replisome can tolerate arabinosyl (ara) analogs, specifically cytarabine (araCTP) and vidarabine (araATP). AraC is in clinical use in treatment against acute myeloid leukemia, whereas araA is primarily used as an antiviral agent due to its rapid deamination in human cells. This study uses S. cerevisiae as a model system, which has the strength of allowing both the use of a complete reconstituted in vitro replication system and genetically modified yeast strains to test models in vivo. The eukaryotic replisome has so far been found to be functionally conserved between humans and yeast. Thus, studies in yeast will be relevant for understanding how the studied nucleoside analogs affect the replisome in human cells. The conclusions drawn in this manuscript can be summarized as follows, araATP and araCTP inhibit DNA synthesis when incorporated into DNA. However, Pol delta proofreads stalled leading strand synthesis and extends DNA synthesis on the leading strand to limit helicase-polymerase uncoupling and intra-S checkpoint activation. Thus, the conclusion is that Pol delta-mediated removal of araA and araC from nascent strands is likely the primary resistance mechanism towards these drugs. This is supported in part by experiments showing that a template-embedded araA or araC does not stall the replisome.

Although the experiments are technically sound and the gels are of high quality, there are some major concerns that need to be raised. The novelty is to a certain degree limited. It has been known for a long time that the proofreading function of Pol epsilon and Pol delta suppresses the inhibitory action of nucleoside analogs considered to be chain terminators. In addition, it is also well documented that Pol delta will proofread errors made by Pol epsilon via a mechanism called extrinsic proofreading. It is therefore expected that Pol delta will proofread incorporated araA and araC. Finally, the nucleotide concentrations used in this series of experiments are of major importance, since there will be competition between ATP/CTP/GTP/UTP, dATP/dCTP/dGTP/dTTP, and araATP or araCTP. A higher concentration of araCTP or araATP relative to the normal nucleotides may lead to an overestimation of how frequently araCTP or araATP is incorporated into the genome. In my opinion, the relative nucleotide concentrations have not been sufficiently addressed. Specific points will follow.

Major concerns.

Page 1. TITLE The title generalizes the findings in this manuscript to all nucleoside analogs. I believe the title should reflect that only two nucleotide analogs were studied, araATP and araCTP (gemcitabine was also studied, but it depletes dNTP pools via inhibition of ribonucleotide reductase).

Page 1. ABSTRACT The abstract follows the same pattern as the title, it generalizes the results from araA and araC as being valid for all nucleoside analogs, which may or may not be true. Please revise the abstract so that it reflects that the study is restricted to araA and araC.

Page 1. ABSTRACT The abstract ends with the following statement: "This study defines the initial response of the eukaryotic replisome to these chemotherapies and may have implications for their use in the clinic." It is not obvious to me how these findings "may have implications for their use in the clinic," given that araC has been in clinical use for at least 55 years. I believe that a finding which truly revolutionizes treatment strategies would be needed for clinicians to change their current practice. This point is elaborated on in the discussion section on page 15, but the question remains, why would clinicians choose chemotherapy when patients harboring exonuclease-disrupting mutations in either or both Pol epsilon and Pol delta respond well to immune checkpoint inhibitor therapy?

Page 4, line 83. The manuscript states: "Since human DNA replication is yet to be fully reconstituted, we utilized ... an established in vitro replication system with purified budding yeast proteins." I understand the need to provide a rationale for the choice of model system. However, the human replication fork has already been reconstituted by the authors, and I believe this study could have been conducted in the human system. I therefore find the current argument somewhat weak and wonder whether a stronger justification exists. Are there meaningful differences between the human and yeast replisome that would favor the yeast system? The tools are available to test this in vitro, even if the human system is unable to activate an origin.

Page 4, line 96. This is the first mentioning of what concentration of araATP was added in specific experiments. The authors first

used 1 µM araATP with little effect and then increased the concentration to 10 µM araATP and saw a clear inhibition of DNA synthesis. I have a series of questions regarding nucleotide concentrations.

a) Why did you choose 1 and 10 µM araATP, respectively?

b) Is there a correlation with the expected in vivo concentration of araATP in the yeast strains described later?

c) I read in the materials and methods that 3.2 mM ATP and 200 µM each of C/G/UTP are used, while 30 µM each of dA/dC/dG/dTTP is used. This does not reflect the estimated in vivo concentrations for these nucleotides. On what basis were these nucleotide concentrations chosen?

d) How will the relative concentrations of araATP, araCTP, NTPs, and dNTPs affect the function of the replisome? The frequency with which araATP and araCTP are incorporated will certainly be influenced, as will the capacity to extend incorporated araATP and araCTP. Furthermore, a reduced frequency of araATP or araCTP incorporation will likely influence the dependence on extrinsic proofreading by Pol delta during leading strand synthesis. The same outcome is likely if the ratio between araNTP and dNTP is altered, e.g. 1:3 (when 10 µM araNTP is used) compared to 1:30 (when 1 µM araNTP is used), or even 1:300.

e) In general, throughout the manuscript, the discussion and arguments regarding the effects of specific nucleotide concentrations are missing or underdeveloped.

Page 5, lines 133-136. "We conclude that under low drug concentrations (1 µM) uncoupling of leading-strand synthesis from helicase unwinding is minimised by Pol proofreading, however this activity has a less significant effect at higher drug concentrations (10 µM)." This sentence illustrates the importance of understanding which nucleotide concentrations should be used to make the study biologically relevant. From a biochemical perspective, it is understandable why the authors chose to focus on what happens in the presence of 10 µM araATP. However, to what extent is this relevant if the in vivo concentration is much lower, and more importantly, lower relative to the concentrations of NTPs and dNTPs? Could you please justify why 10 µM araATP is a more relevant condition than 1 µM araATP?

Page 5, line 147. "Since Pol epsilon proofreading does not significantly affect replication rate in the presence of 10 µM araATP (Fig. 1F), and Pol alpha cannot support efficient leading-strand synthesis under these conditions (Yeeles et al., 2017),..." Pol alpha is needed for the initiation of leading strand synthesis as well as for Okazaki fragment synthesis during lagging strand synthesis in the replisome. Please add in vitro assays that directly analyze the inhibition of Pol alpha. There is a paragraph discussing the effect of Pol alpha, however, the reasoning is based on products that are extended by Pol delta, which is itself also affected by the added araNTP. Could you please clarify where and how the inhibition of Pol alpha influences the results in the biochemical analysis? This is particularly important for interpreting the in vivo results. Pol alpha will most likely be inhibited in yeast cells, if so, how will this affect cell growth in the presence or absence of Pol delta proofreading? It should also be noted that Pol delta proofreads Pol alpha and extyends primers synthesize dby Pol alpha, both when initiating leading strand synthesis and during lagging strand synthesis.

Page 14, lines 437-438. It is concluded that the observed effect of gemcitabine on yeast cells is most likely caused by inhibition of ribonucleotide reductase. Is it known whether araA or araC influence dNTP pools in vivo, either by modifying the activity of ribonucleotide reductase or through some other enzyme involved in nucleotide metabolism? Altered dNTP pools may shift the balance between exonuclease and polymerase activity as well as the processivity and rate of synthesis.

We are very grateful to the editors and referees for their time in critically assessing our manuscript. Each comment has been addressed in our point-by-point responses below. The new manuscript has been revised accordingly, with any text changes highlighted in yellow.

Referee #1:

The manuscript by Orrin and Yeeles reports a detailed analysis of how the yeast replisome handles arabinosyl (ara) nucleoside analogues that are commonly used as chemotherapeutic agents in humans. The authors show that DNA polymerase delta plays a major role in sustaining replication in the presence of ara analogues, taking over DNA synthesis and proofreading on the leading strand, and limiting polymerase-helicase uncoupling, with these functions becoming more prominent when DNA polymerase epsilon proofreading is defective. The work uses a sophisticated arsenal of tools, including the complete replisome reconstituted in vitro and a genetic system allowing DNA polymerase assignment to leading and lagging strands. These approaches are almost too good for the question at hand, as the significance of a detailed understanding of how yeast proteins interact with specific human drugs is uncertain. Nevertheless, the study is of high quality and reveals the dynamics of replication machinery when challenged with chain-terminating nucleoside analogues. The following revisions would strengthen the conclusions and improve clarity.

We are pleased the reviewer determines our study to be of high quality and we appreciate the constructive feedback provided.

Major comments:

1) Please provide quantification of relevant endpoints and statistical analysis for all gels, and also include indicators of statistical significance in the drug sensitivity assays.

We apologise for the lack of statistical significance indicators present in our quantification of drug sensitivity assays presented in **Figures 5B and EV5E**. We have now performed one-way ANOVA analyses for each condition at the 5-day timepoint, followed by post-hoc Tukey's multiple comparisons tests between strains when significance was detected (*p*-values reported in **Appendix Table 4**). These results support our initial conclusions, and we have updated **Figures 5B and EV5E** and their legends accordingly.
We have quantified relevant lanes of gels presented in **Figures 1F, 1G, 2A, 2B, 2D, 3A, 3B, 3C, 4B, 6C, 6F**, and have additionally calculated maximum synthesis rates of reactions

presented in **Figure 2E** of the revised manuscript (**Appendix Figure 5**). These results support our initial conclusions.
The Methods section has been updated to include a 'Quantification and Statistical Analysis' section, describing how these analyses were performed.

2) All nucleoside incorporation experiments were done with araATP, but studies of template-embedded analogues only included araC and gemC. Please discuss this discrepancy and how it affects the overall conclusions.

By comparing two template-embedded cytidine analogues, we were able to directly test the relative effects of either a difluororibose (gemC) or arabinose (araC) sugar moiety on DNA synthesis. The fact that both are cytidine analogues also enabled their incorporation at precisely the same position on each template, eliminating the possible influence of sequence context on stalling frequency. Nevertheless, we would expect a template-embedded araA to stall DNA synthesis to a similar extent. We have now added an *in vitro* DNA replication experiment (**Fig. EV4A**) showing that like araATP, araCTP inhibits synthesis in this system in a dose-dependent manner.

3) Experimental details are not always explained clearly. The study alternates between using circular and linear DNA substrates, providing no rationale for the substrate choice and sometimes confusing them (see minor comments for examples). I suggest moving Figure EV1D to the main text. Figure panels also need more detailed titles and legends.

We apologise for the lack of clarity regarding use of different DNA substrates. The use of a circular dsDNA template enabled us to assess helicase-polymerase uncoupling by monitoring U* formation (e.g. **Figure 1D, native gel**). However, owing to bidirectional replication from a single origin (ARS306), leading strands are synthesised by two potentially asynchronous replication fork populations giving rise to a broad distribution of leading-strand products (e.g. **Figure 1D, denaturing gel**). In contrast, use of a linear template where ARS306 is asymmetrically positioned generates two differentially sized leading-strand products (8.2 kb and 1.5 kb), each synthesised by a single replication fork population. The length distribution of these products is thus tighter, making it simpler to detect differences in synthesis rates. We now explain this in the main text and, as the reviewer suggests, have moved Figure EV1D in the original manuscript to **Figure 1E**. We have also updated figure titles and legends to include more detail throughout the work.

4) The model in Figure 7 is overreaching. It proposes a very specific scenario not directly supported by the data. There does not seem to be evidence that ara nucleotides specifically affect the handover to pol epsilon at origins. Why cannot they affect pol epsilon processivity during elongation? It would be better to end with a more general model that this study directly supports and discuss different hypotheses for how pol delta participation in leading strand synthesis could be enhanced.

The reviewer is correct. We have not directly tested the effects of ara nucleotides on the establishment of Pol ε-catalysed leading-strand synthesis, and we certainly do not intend to convey that these drugs have no effect on Pol ε processivity during elongation. We instead think that both scenarios may occur with a likelihood that is influenced by local drug concentration and polymerase proofreading. We have therefore updated **Figure 7** to focus primarily on leading-strand elongation, and we suggest in the Discussion that ara chemotherapies may additionally influence the establishment of CMGε leading-strand synthesis during initiation.

5) The discussion of implications for cancer therapy (p. 15-16) lacks depth. The various statements in this paragraph appear disconnected. Ultimately, it remains unclear how the findings in this work could help improve therapy and for which cancers.

We thank the reviewer for their critical feedback of our discussion point. Upon reflection, we agree that whilst our work has uncovered a critical role for Pol δ proofreading in resisting the cytotoxic effects of ara chemotherapies, the immediate relevance of this in a clinical setting is not as obvious as we have implied. We have therefore removed this section and instead suggest that the ability of Pol δ to remove ara analogues from both nascent lagging and leading strands may act as a possible resistance mechanism against these chemotherapies when used in the clinic.

Minor comments:

6) P. 3, ln. 71: Change "in this manuscript" to "in this study" or similar.

We have replaced 'manuscript' with 'study'.

7) P. 4, ln. 101: Delete "corresponding" unless supported by quantification (the increase in theta intermediates seems very small compared to the loss of LRIs).

We have removed the term ‘corresponding’.

8) P. 5, ln. 130: "under these conditions" seems incorrect: unlike Fig. 1E, Fig. EV1E shows replication of the circular template.

We have replaced ‘under these conditions’ with ‘under similar conditions’.

9) P. 6, ln. 162-163: The conclusion sounds premature. The experiments described in the following paragraphs, indeed, provide evidence for pol delta synthesis on the leading strand, but this paragraph only describes inhibition of synthesis by the catalytically dead pol delta. I suggest rephrasing the conclusion.

We agree with the reviewer and, in light of Referee #2’s related feedback regarding **Figure 2**, we now present the catalytically dead Pol delta experiment later to avoid premature conclusions.

10) P. 6, ln. 172: "we repeated the experiment" is incorrect (again, different assays were used in Fig. 2C and Fig. EV2D).

We have replaced ‘we repeated the experiment’ with ‘we performed a similar experiment’.

11) P. 9, ln. 260-261: "began to sensitize cells at higher concentrations" - the difference seems to be in the assays, not concentrations. Figure EV5D shows no effect at 40 uM concentration.

We agree with the reviewer, however the scans in **Figure EV5D** show cell growth at an early stage (3 days, 25°C). In **Figure EV5E**, the difference becomes more obvious at later timepoints (5 days, 30°C). Consistent with this, scans taken at a later stage (5 days, 25°C) do begin to show a small effect (see below, compare top and bottom). Since **Figure EV5E** represents quantification from three independent yeast cultures, we would like to keep this sentence in the manuscript.

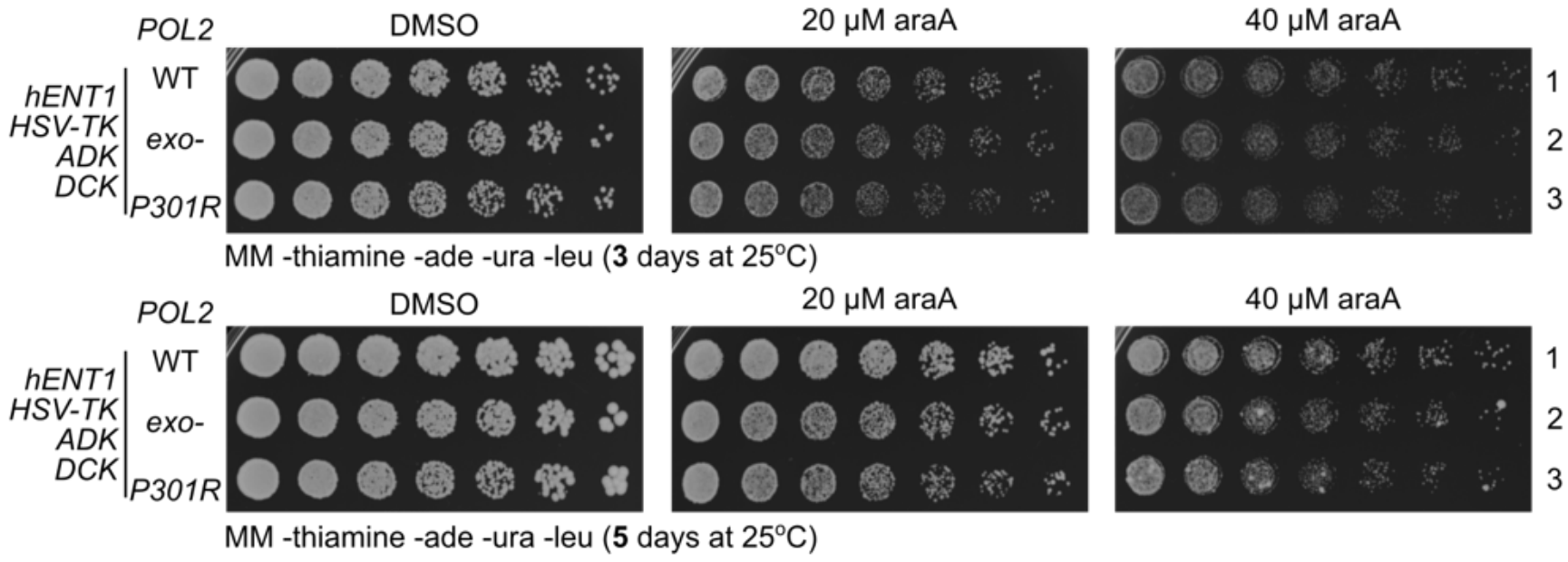

12) P. 29, ln. 520-524: "Figures were performed" should read "Assays/experiments/reactions in figures were performed".

We have corrected this.

13) Materials and Methods subtitles "Budding yeast replication reactions" are misleading, as the reactions are with purified proteins rather than yeast.

We have replaced 'Budding yeast replication reactions' with 'replication reactions with purified budding yeast proteins'.

14) Figures 2A and EV2A-B: indicate which pol epsilon variant was used.

We have added 'With Pol ε' to these figures.

15) Figure 4B-C: indicate that the reaction products were digested with SmaI.

We have added additional text in the legend to indicate reactions in **Figure 4B** were treated post-quench with SmaI. However, reactions in **Figure 4C** were not treated with SmaI so we have not modified it further.

16) Figure EV3A legend: "as in Figure 1C" - the DNA template in Figure 1C is circular.

We have replaced 'as in Figure 1C' with 'as in **Figure 1E**' which in the updated manuscript outlines replication reactions performed on a linear DNA template.

17) Figure EV4 legend is confusing.

We have altered **Figure EV4** legend for clarity.

Referee #2:

This manuscript focuses on the DNA replication of nucleotide analogues (NAs) and employs both an in vitro assay using a reconstituted replication system and in vivo assays, including measurements of polymerase usage. This is an extensive study that addresses changes in DNA polymerase functions in response to NAs and provides valuable case studies illustrating how replication forks and their components can be flexible. In my opinion, while the overall structure of this study is sound and well described, I have several comments that may help further improve the manuscript.

We appreciate the positive assessment of our study and the constructive suggestions to further improve our manuscript.

Figure 1:
At 10 μM, araATP causes a clearly more severe perturbation of both leading and lagging strand synthesis. I wonder whether the authors can exclude the possibility that this strong effect is due to inhibition of DNA unwinding by the MCM helicase.

We thank the reviewer for this interesting question. Whilst we cannot exclude that araATP inhibits DNA unwinding by the CMG helicase, we think it is unlikely to contribute to DNA synthesis inhibition in our reactions for the following reasons:
1) ATP is in 320-fold molar excess over araATP (3.2 mM vs 10 uM).
2) Even if unwinding is attenuated, the formation of U* (e.g. **Figure 1D, native**) demonstrates that DNA polymerases must be inhibited to a greater extent.
3) Addition of araCTP, which is unlikely to be utilised by the CMG helicase, has a similar dose-dependent effect on synthesis compared with araATP (see updated **Fig. EV4A**).

Figure 2:
While the data support the conclusions, the explanation of the experiments is confusing, taking several reads to understand the data presented. We suggest this could be simplified by presenting the information in an alternative order, presenting Figure 2B, 2C, then 2A. This would establish the effect of Pol delta omission first, then move to the confirmatory pulse-chase experiment, which is relatively complicated. An interpretation should be provided for the lack of difference observed with WT Pol epsilon in Figures 2B and 2C, in contrast to Figure 2A, and also why exo- gives different results.

We thank the reviewer for their suggestions regarding alternative presentation of experiments in Figure 2. We agree that presenting the more complex pulse-chase experiment later provides greater clarity to the reader and avoids premature conclusions.

We have rearranged **Figure 2** in the updated manuscript as follows with accompanying rationale:

Figure 2E/C → 2A/B (Pol ε^{exo-}, 1/10 μM araATP)

Presenting Figures 2E/C first enables us to demonstrate dependence on Pol δ for leading-strand synthesis in the presence of a proofreading-defective Pol ε, as hinted at in Figure 1.

Figures 2D/B → 2C/D (Pol ε, 1/10 μM araATP)

Figures 2D/B then show that, surprisingly, omission of Pol δ has little effect on leading-strand synthesis rates in the presence of wild-type Pol ε despite helicase-polymerase uncoupling occurring at this drug concentration (Fig. 1D). This either indicates that Pol δ has no role on the leading strand when wild-type Pol ε is present, or that the synthesis rates of Pol ε and Pol δ are equal under these conditions since they are both limited by the time taken to remove ara nucleotides from the nascent strands.

Figure 2A → 2E (pulse-chase, 10 μM araATP)

To distinguish between these possibilities, we perform a pulse-chase experiment showing that a polymerase-dead Pol δ mutant inhibits leading-strand synthesis by wild-type Pol ε. Assuming that this polymerase-dead Pol δ mutant behaves similarly to the wild-type enzyme, we therefore conclude that Pol δ is the leading-strand polymerase under these conditions.

We have additionally rearranged **Figure EV2** to reflect the updated order of the main figure.

Figure 3:

The description of results in the "Pol δ proofreads the leading strand..." section is not clearly matched to what the section title and Figure 3 aim to show. A description of how the assay/results demonstrate leading strand proofreading should be provided to aid the reader.

Since we have established that Pol δ synthesises the leading strand in the presence of 10 uM araATP (**Figure 2**) and that a proofreading-defective form of the enzyme leads to a reduction in leading-strand synthesis rates (**Figure 3**), we think the simplest explanation is that Pol δ proofreads the leading strand to remove ara nucleotides. However, the reviewer is correct in that our experiments do not specifically assess ara nucleotide incorporation into nascent DNA.

We have therefore updated the results section to explicitly mention that the reduction in leading-strand synthesis rates upon loss of Pol δ proofreading suggests that Pol δ both synthesises and proofreads nascent leading strands.

Figure 6:
This figure provides strong in vivo evidence for the involvement of Pol delta in leading-strand DNA synthesis. To show that DNA fragmentation results from cleavage at ribonucleotides, rather than from the nature of the pol2-exo- cells, araA treatment, or their combination, I suggest that the authors explain this part more carefully using the control experiments. For example, they could directly compare "POL3-WT pol2-exo- + araA" in Figure EV6E, lane 14, with "pol3-L612G pol2-exo- + araA" in Figure 6F, lane 8.

We are grateful to the reviewer for their advice on clarifying the results presented in **Figure 6**. We now include specific lane comparisons between **Figures 6F and EV6E** in the revised manuscript to better highlight our control experiments.

Minor:
In lines 177-180, I suggest that the authors avoid discussing differences between samples from different experimental batches.

We have removed this sentence in the revised manuscript.

There appears to be a dominant negative effect in Figure 3B, where having Pol deltaexo- is worse than omitting Pol dleta (Figure 2B). Could the authors explain the cause of this in the text? Is this due to different experiment baches?

Figure 3B shows the effect of Pol δ^{exo-} on synthesis in the presence of Pol ε^{exo-}, whereas Figure 2B (**Figure 2D** in the revised manuscript) shows the effect of omitting Pol δ in the presence of wild-type Pol ε. We wonder whether the reviewer intends to compare **Figure 3A** with Figure 2B (**Figure 2D** in the revised manuscript), both of which contain wild-type Pol ε. Comparison of these figures does indeed show a Pol δ^{exo-} dominant-negative effect on synthesis in the presence of araATP relative to when Pol δ is omitted. This is presumably because a polymerase that is able to remove ara nucleotides and thus extend the nascent strand (Pol ε) is replaced by one that cannot (Pol δ^{exo-}), thus supporting our conclusions that Pol δ is the leading-strand polymerase under these conditions.

Please provide the amino acid changes in the Pol epsilon exo- mutant when first mentioned.

We have added the D290A, E292A mutations when first introducing the Pol ε^{exo-} mutant in the revised manuscript. We have additionally added the D321A, E323A mutations when first introducing the Pol δ^{exo-} mutant.

In lines 404-406, the authors suggest a role for CTF18-RFC in minimizing the uncoupling of leading-strand synthesis. However, in Figure 1D, they did not observe a clear effect upon addition of this factor. I wonder how the authors reconcile these two parts.

We thank the reviewer for this interesting discussion point. In lines 404-406 of the original manuscript, we cite work that demonstrates loss of CTF18-RFC sensitises cells to araC and suggest that this may contribute to minimising uncoupling *in vivo*. Indeed, in a cellular context where PCNA unloading pathways contribute to PCNA flux, a dedicated leading-strand clamp loader may be important in minimising uncoupled synthesis. Our *in vitro* replication reactions do not have these PCNA unloading pathways and, indeed, we do not know the frequency that loaded PCNA dissociates from our templates. It may therefore simply be that PCNA loading is not limiting in our reactions and thus does not influence uncoupling to such an extent that can be observed from our gels.

Referee #3:

The aim of this manuscript is to explain how the replisome can tolerate arabinosyl (ara) analogs, specifically cytarabine (araCTP) and vidarabine (araATP). AraC is in clinical use in treatment against acute myeloid leukemia, whereas araA is primarily used as an antiviral agent due to its rapid deamination in human cells. This study uses S. cerevisiae as a model system, which has the strength of allowing both the use of a complete reconstituted in vitro replication system and genetically modified yeast strains to test models in vivo. The eukaryotic replisome has so far been found to be functionally conserved between humans and yeast. Thus, studies in yeast will be relevant for understanding how the studied nucleoside analogs affect the replisome in human cells. The conclusions drawn in this manuscript can be summarized as follows, araATP and araCTP inhibit DNA synthesis when incorporated into DNA. However, Pol delta proofreads stalled leading strand synthesis and extends DNA synthesis on the leading strand to limit helicase-polymerase uncoupling and intra-S checkpoint activation. Thus, the conclusion is that Pol delta-mediated removal of araA and araC from nascent strands is likely

the primary resistance mechanism towards these drugs. This is supported in part by experiments showing that a template-embedded araA or araC does not stall the replisome.

Although the experiments are technically sound and the gels are of high quality, there are some major concerns that need to be raised. The novelty is to a certain degree limited. It has been known for a long time that the proofreading function of Pol epsilon and Pol delta suppresses the inhibitory action of nucleoside analogs considered to be chain terminators. In addition, it is also well documented that Pol delta will proofread errors made by Pol epsilon via a mechanism called extrinsic proofreading. It is therefore expected that Pol delta will proofread incorporated araA and araC. Finally, the nucleotide concentrations used in this series of experiments are of major importance, since there will be competition between ATP/CTP/GTP/UTP, dATP/dCTP/dGTP/dTTP, and araATP or araCTP. A higher concentration of araCTP or araATP relative to the normal nucleotides may lead to an overestimation of how frequently araCTP or araATP is incorporated into the genome. In my opinion, the relative nucleotide concentrations have not been sufficiently addressed. Specific points will follow.

We are pleased that the reviewer considers our work to be technically sound. We agree that it is well documented that Pol δ proofreads errors for Pol ε on the leading strand, and we have referenced these studies in the original manuscript at the earliest opportunity (page 7, lines 182-183). However, we think our work advances mechanistic understanding of this process by showing that helicase-polymerase uncoupling on the leading strand regulates Pol δ access. We should also point out that the notion that Pol δ proofreading promotes resistance against ara nucleosides has only recently been shown *in vivo*, as cited in our work (page 8, line 237).

Major concerns.

Page 1. TITLE The title generalizes the findings in this manuscript to all nucleoside analogs. I believe the title should reflect that only two nucleotide analogs were studied, araATP and araCTP (gemcitabine was also studied, but it depletes dNTP pools via inhibition of ribonucleotide reductase).

The reviewer is correct. Our major findings address the effects of araA and araC and their triphosphate derivatives on replisome behaviour and cell viability. We therefore have reworded the title to emphasise our study on arabinosyl analogues.

Page 1. ABSTRACT The abstract follows the same pattern as the title, it generalizes the results from araA and araC as being valid for all nucleoside analogs, which may or may not be true. Please revise the abstract so that it reflects that the study is restricted to araA and araC.

We again agree with the reviewer. We have rephrased our abstract in the revised manuscript to stipulate which analogues we have studied and suggest at the end that our results may be applicable to other chain-terminating analogues.

Page 1. ABSTRACT The abstract ends with the following statement: "This study defines the initial response of the eukaryotic replisome to these chemotherapies and may have implications for their use in the clinic." It is not obvious to me how these findings "may have implications for their use in the clinic," given that araC has been in clinical use for at least 55 years. I believe that a finding which truly revolutionizes treatment strategies would be needed for clinicians to change their current practice. This point is elaborated on in the discussion section on page 15, but the question remains, why would clinicians choose chemotherapy when patients harboring exonuclease-disrupting mutations in either or both Pol epsilon and Pol delta respond well to immune checkpoint inhibitor therapy?

We appreciate this feedback. We certainly did not intend to suggest that our results revolutionise treatment strategies and necessitate a change in clinical practice. The aim was instead to simply highlight the fact that, as the reviewer correctly states, mutations in DNA polymerase proofreading domains are biomarkers for cancers that respond well to immunotherapy approaches and as such could also be for ara analogues. In light of both this criticism and related comments from Referee #1, we have decided to remove this part of the discussion and instead suggest our results have implications for how resistance may arise against arabinosyl chemotherapies, and possible other chain-terminators, when used in the clinic.

Page 4, line 83. The manuscript states: "Since human DNA replication is yet to be fully reconstituted, we utilized ... an established in vitro replication system with purified budding yeast proteins." I understand the need to provide a rationale for the choice of model system. However, the human replication fork has already been reconstituted by the authors, and I believe this study could have been conducted in the human system. I therefore find the current argument somewhat weak and wonder whether a stronger justification exists. Are there meaningful differences between the human and yeast replisome that would favor the yeast

system? The tools are available to test this in vitro, even if the human system is unable to activate an origin.

We thank the reviewer for giving us further opportunity to explain our choice of model system. DNA synthesis in the human system begins from a pre-assembled CMG helicase loaded onto a linear, primed replication fork substrate. In contrast, DNA synthesis in the yeast system is fully reconstituted at an origin, with bidirectional leading-strand synthesis being established following the synthesis of the initial lagging-strand primer. As such, use of the yeast system enabled us to test the effects of ara nucleotides not only during elongation, but during origin activation. Both scenarios would likely be affected *in vivo* and are addressed in our updated model (**Figure 7**) and Discussion following feedback from Referee #1. Furthermore, the ability to fully reconstitute budding yeast replisomes permitted us to perform reactions on circular templates, which was essential for assessing the effects of ara analogues on helicase-polymerase uncoupling by proxy of U* formation (e.g. **Figure 1D**). Finally, we felt that *in vitro* work with purified budding yeast proteins would better complement our *in vivo* data including those assessing polymerase usage near an origin (**Figure 6**)- experiments uniquely achievable in budding yeast. We also note that the core architecture of yeast and human replisomes are similar, and the polymerase division of labour at forks under normal conditions appears to be conserved. Taken together, we therefore decided that the yeast system was most appropriate for the mechanistic questions addressed in this study.

Page 4, line 96. This is the first mentioning of what concentration of araATP was added in specific experiments. The authors first used 1 μM araATP with little effect and then increased the concentration to 10 μM araATP and saw a clear inhibition of DNA synthesis. I have a series of questions regarding nucleotide concentrations.

a) Why did you choose 1 and 10 μM araATP, respectively?

These concentrations were initially chosen based on the relative levels of inhibition observed in our *in vitro* replication experiments. By comparing the effects of two drug concentrations, we were able to confirm that DNA synthesis in this system is inhibited in a dose-dependent manner and show that the relative contributions of Pol ε and Pol δ to leading-strand replication is also dependent on drug dose, which we show relates to the extent of helicase-polymerase uncoupling.

b) Is there a correlation with the expected in vivo concentration of araATP in the yeast strains described later?

We do not know the precise level of the active, triphosphorylated form of araA (araATP) *in vivo* since this will likely be regulated by ADK/DCK (and Ado1, **Figure EV5A**) and possibly other competing metabolic pathways. However, it has been shown that the triphosphorylated form of a fluorinated derivative of araA, fludarabine (F-araA), can accumulate to the uM range in blood cells (Gandhi & Plunkett, 2002).

c) I read in the materials and methods that 3.2 mM ATP and 200 μM each of C/G/UTP are used, while 30 μM each of dA/dC/dG/dTTP is used. This does not reflect the estimated in vivo concentrations for these nucleotides. On what basis were these nucleotide concentrations chosen?

In budding yeast, the estimated *in vivo* concentrations (uM) of A/C/G/UTP are 3000, 500, 700, and 1250, respectively. The estimated *in vivo* concentrations (uM) of dA/dC/dG/dTTP are lower at 16, 14, 12, and 30, respectively (Williams & Kunkel, 2022). Whilst not identical, we hope the reviewer will agree that the concentrations used in our replication reactions are comparable and, as shown previously, support synthesis rates similar to those observed *in vivo* (Yeeles *et al*, 2017).

d) How will the relative concentrations of araATP, araCTP, NTPs, and dNTPs affect the function of the replisome? The frequency with which araATP and araCTP are incorporated will certainly be influenced, as will the capacity to extend incorporated araATP and araCTP. Furthermore, a reduced frequency of araATP or araCTP incorporation will likely influence the dependence on extrinsic proofreading by Pol delta during leading strand synthesis. The same outcome is likely if the ratio between araNTP and dNTP is altered, e.g. 1:3 (when 10 μM araNTP is used) compared to 1:30 (when 1 μM araNTP is used), or even 1:300.

We agree with the reviewer that the relative concentrations of araNTPs and (d)NTPs will influence how frequently araNTPs are incorporated and extended from, and the dependence on Pol δ for leading-strand synthesis. Indeed, we have shown that DNA synthesis inhibition mediated by these analogues is dose-dependent (**Figures 1, 2, and 6**) and that the extent of inhibition influences the usage of Pol δ on the leading strand *in vitro* (**Figure 2**) and *in vivo* (**Figure 6**).

e) In general, throughout the manuscript, the discussion and arguments regarding the effects of specific nucleotide concentrations are missing or underdeveloped.

We respectfully disagree with the reviewer. In all *in vitro* and *in vivo* experiments where the effect of araN(TP) addition is tested, we clearly state the concentration used, show that concentration modulates replisome behaviour (as above, and see below) and indeed incorporate the effect of drug concentration into our final model (**Figure 7**). However, based on the reviewer's comments we have further discussed the effects of 1 μM vs 10 μM araATP on Pol δ usage on the leading strand when reporting results presented in **Figure 2**.

Page 5, lines 133-136. "We conclude that under low drug concentrations (1 μM) uncoupling of leading-strand synthesis from helicase unwinding is minimised by Pol proofreading, however this activity has a less significant effect at higher drug concentrations (10 μM)." This sentence illustrates the importance of understanding which nucleotide concentrations should be used to make the study biologically relevant. From a biochemical perspective, it is understandable why the authors chose to focus on what happens in the presence of 10 μM araATP. However, to what extent is this relevant if the in vivo concentration is much lower, and more importantly, lower relative to the concentrations of NTPs and dNTPs? Could you please justify why 10 μM araATP is a more relevant condition than 1 μM araATP?

By using two different drug concentrations (1 and 10 μM), we have been able to modulate the extent of helicase-polymerase uncoupling and show that this influences the ability of Pol δ to access and proofread the leading strand *in vitro* (**Figures 1-3**). Such drug concentrations are shaped by the sensitivity of our experimental setup, which uses bulk synthesis rates as a readout for Pol δ activity. Importantly, we have not claimed, and have no wish to claim, that one concentration is more biologically relevant than another. Rather, the biochemical studies establish a principle of replisome response to ara analogues which we then use to design subsequent experiments to test whether the same response occurs *in vivo* at drug concentrations that are, again, appropriate to the experimental setup (**Figures 5-6**).

Page 5, line 147. "Since Pol epsilon proofreading does not significantly affect replication rate in the presence of 10 μM araATP (Fig. 1F), and Pol alpha cannot support efficient leading-strand synthesis under these conditions (Yeeles et al., 2017),..." Pol alpha is needed for the initiation of leading strand synthesis as well as for Okazaki fragment synthesis during lagging strand synthesis in the replisome. Please add in vitro assays that directly analyze the inhibition of Pol alpha.

We agree with the reviewer that Pol α is required for the initiation of leading-strand synthesis and Okazaki fragment synthesis, as supported by much of our previous work. The sentence written on page 5, line 147 of the original manuscript addressed the fact that under our replication reaction conditions where PCNA loading is permitted, Pol α still synthesises short primers but is unable to synthesise leading strands of any considerable length (see Figure 6A of Yeeles *et al*, 2017). Based on this, we explore the role of Pol δ on leading-strand synthesis (**Figure 2**). We have added a primer extension reaction as **Figure EV2B** showing that Pol α synthesis is inhibited by araATP (as observed for Pol ε and Pol δ) and have referenced this in the results section, and updated the Methods section accordingly.

There is a paragraph discussing the effect of Pol alpha, however, the reasoning is based on products that are extended by Pol delta, which is itself also affected by the added araNTP. Could you please clarify where and how the inhibition of Pol alpha influences the results in the biochemical analysis? This is particularly important for interpreting the in vivo results. Pol alpha will most likely be inhibited in yeast cells, if so, how will this affect cell growth in the presence or absence of Pol delta proofreading? It should also be noted that Pol delta proofreads Pol alpha and extyends primers synthesize dby Pol alpha, both when initiating leading strand synthesis and during lagging strand synthesis.

The fact that we observe leading-strand synthesis in reactions containing either 1 or 10 μM araATP demonstrates that Pol α still exhibits sufficient activity to extend RNA primers that are handed over to Pol δ. We agree with the reviewer that Pol α is likely inhibited in yeast cells but the fact that modulating the proofreading activity of Pol δ affects cell sensitivity to ara analogues (**Figure 5**) indicates Pol α continues to lay down primers that Pol δ subsequently extends.

Page 14, lines 437-438. It is concluded that the observed effect of gemcitabine on yeast cells is most likely caused by inhibition of ribonucleotide reductase. Is it known whether araA or araC influence dNTP pools in vivo, either by modifying the activity of ribonucleotide reductase or through some other enzyme involved in nucleotide metabolism? Altered dNTP pools may shift the balance between exonuclease and polymerase activity as well as the processivity and rate of synthesis.

A select number of publications report that araATP and F-araATP can inhibit ribonucleotide reductase (RNR) activity (White *et al*, 1982). We therefore cannot exclude the possibility that inhibition of RNR and/or other enzymes involved in nucleotide metabolism enzymes

additionally contributes to the cytotoxicity of ara analogues *in vivo*. However, most literature addressing the modes-of-action of araA/C suggests these drugs primarily inhibit DNA synthesis following incorporation into nascent strands by DNA polymerases (e.g. Townsend & Cheng, 1987), consistent with our primer extension and replication experiments (e.g. **Figure 1**). We have also found that, unlike for araA/CTP, addition of 20 μM gemCTP does not inhibit DNA synthesis in our replication reactions (now added as **Fig. EV4B**). Taken together with the fact that gemC is more potent than araC *in vivo* (**Figure 5A**), this leads us to conclude that its primary mode-of-action is its ability to inhibit RNR.

References

Gandhi V & Plunkett W (2002) Cellular and Clinical Pharmacology of Fludarabine. *Clin Pharmacokinet* 41: 93–103

Townsend AJ & Cheng YC (1987) Sequence-specific effects of ara-5-aza-CTP and ara-CTP on DNA synthesis by purified human DNA polymerases in vitro: visualization of chain elongation on a defined template. *Mol Pharmacol* 32: 330–339

White EL, Shaddix SC, Brockman RW & Bennett LL (1982) Comparison of the actions of 9-beta-D-arabinofuranosyl-2-fluoroadenine and 9-beta-D-arabinofuranosyladenine on target enzymes from mouse tumor cells. *Cancer Res* 42: 2260–2264

Williams JS & Kunkel TA (2022) Ribonucleotide Incorporation by Eukaryotic B-Family Replicases and Its Implications for Genome Stability. *Annu Rev Biochem* 91: 133–155

Yeeles JTP, Janska A, Early A & Diffley JFX (2017) How the Eukaryotic Replisome Achieves Rapid and Efficient DNA Replication. *Mol Cell* 65: 105–116

Dr. Joseph TP Yeeles
MRC Laboratory of Molecular Biology (LMB)
Protein and Nucleic Acids Chemistry
Francis Crick Avenue
Cambridge CB2 0QH
United Kingdom

27th Jul 2026

Re: EMBOJ-2026-124467R
How the eukaryotic replisome tolerates arabinosyl nucleoside analogue chemotherapies

Dear Joe,

Thank you for submitting your revised manuscript on replisome tolerance of arabinosyl nucleoside analogs for our consideration. Two of the original referees have now reviewed it once more, and overall found the study significantly strengthened and most major concerns adequately addressed. Referee 3 retains a few presentational reservations, which I would ask you to incorporate in a final round of minor revision. In addition, there are still a few editorial modifications to be made at this stage:

- Please adjust the order of the manuscript sections, and also make sure to use the correct section headers:
Single title page with complete author information, Abstract, Introduction, Results, Discussion, Methods, Data Availability, Acknowledgements, Disclosure and Competing Interests Statement, References, Main Figure Legends, Tables, Expanded Figure Legends.

- As we are switching from a free-text author contribution statement towards a more formal statement based on Contributor Role Taxonomy (CRediT) terms, please remove the present Author Contribution section and instead specify each author's contribution(s) directly in the Author Information page of our submission system during upload of the final manuscript. See https://casrai.org/credit/ for more information.

- Please carefully go through the reference list and make sure that all references have their complete citation information - including year, journal name & volume, and page/elocator numbers - some of this information is currently missing for several of them.

- For preprint citations: Please change in-text reference format according to "preprint: Miller et al, 2025"; and in the reference list, name of the platform plus DOI + preprint label - e.g. "bioRxiv doi: 1234/002.dfj123 [PREPRINT]". (Btw, please double-check if the preprint by Hill et al is actually referenced somewhere in text).

- Please provide the Appendix as a PDF file, making sure to remove any remnaining change-highlight marks. On the title page with the ToC, please add the header 'Appendix for [manuscript title]'. Finally, please double-check that Appendix Table S1 is referenced at least once from the main manuscript.

- The Data Availability section should only refer to any newly generated datasets deposited in public repositories; should there be none, all it should state is "This study includes no data deposited in external repositories."

- Please provide suggestions for a short 'blurb' text prefacing and summing up the conceptual aspect of the study in two sentences (max. 250 characters), followed by 3-5 one-sentence 'bullet points' with brief factual statements of key results of the paper; they will form the basis of an editor-written 'Synopsis' accompanying the online version of the article. Please also upload a synopsis image, which can be used as a "visual title" for the synopsis section of your paper. The image should be in JPG format of 550 pixels wide and 300 pixels high, and please make sure that it remains clear enough even in these modest dimensions.

- Finally, during routine pre-acceptance checks, our data editors have raised the following queries regarding figures, data, and legends; I would appreciate if you briefly answered to them in the cover letter of your final submission, and made the requested text modifications with changes/additions highlighted via the "Track changes" option, to facilitate our final checking.
1. Please provide the exact p values in the legends of figures 5B; EV-5B.
2. Please add information related to 'n' in the legends of figure EV-5B.
3. Please define the measure of center for the error bars in the legends of figures 5B; EV-5B.

I am returning the manuscript to you for minor revision, hoping you will be able to return the final modified files within the course of this week. Once we will have received them, we should be ready to swiftly proceed with formal acceptance and production of the manuscript.

With kind regards,

Hartmut

Hartmut Vodermaier, PhD
Senior Editor, The EMBO Journal
h.vodermaier@embojournal.org

*** PLEASE NOTE: All revised manuscript are subject to initial checks for completeness and adherence to our formatting guidelines. Revisions may be returned to the authors and delayed in their editorial re-evaluation if they fail to comply to the following requirements. As a first step please read our guidelines for revised submissions: https://link.springer.com/journal/44318/submission-guidelines#cms-Revised-submissions

1) Every manuscript requires a Data Availability section (even if only stating that no deposited datasets are included). Primary datasets or computer code produced in the current study have to be deposited in appropriate public repositories prior to resubmission, and reviewer access details provided in case that public access is not yet allowed.

2) Each figure legend must specify
- size of the scale bars that are mandatory for all micrograph panels
- the statistical test used to generate error bars and P-values
- the type error bars (e.g., S.E.M., S.D.)
- the number (n) and nature (biological or technical replicate) of independent experiments underlying each data point
- Figures may not include error bars for experiments with n<3; scatter plots showing individual data points should be used instead.

3) Revised manuscript text (including main tables, and figure legends for main and EV figures) has to be submitted as editable text file (e.g., .docx format). We encourage highlighting of changes (e.g., via text color) for the referees' reference.

4) Each main and each Expanded View (EV) figure should be uploaded as individual production-quality files (preferably in .eps, .tif, .jpg formats). For suggestions on figure preparation/layout, please refer to our Figure Preparation Guidelines: https://media.springernature.com/original/springer-cms/rest/v1/content/27825798/data/v1

5) Point-by-point response letters should include the original referee comments in full together with your detailed responses to them (and to specific editor requests if applicable), and also be uploaded as editable (e.g., .docx) text files.

6) Please complete our Author Checklist, and make sure that information entered into the checklist is also reflected in the manuscript; the checklist will be available to readers as part of the Review Process File.

7) All authors listed as (co-)corresponding need to deposit, in their respective author profiles in our submission system, a unique ORCiD identifier linked to their name. Please see our Guide to Authors for detailed instructions.

8) Please note that supplementary information at EMBO Press has been superseded by the 'Expanded View' for inclusion of additional figures, tables, movies or datasets; with up to five EV Figures being typeset and directly accessible in the HTML version of the article.

9) To facilitate reproducibilty and cross-laboratory adoption of methodologies, please structure the Materials & Methods section as outlined in our guide to authors, including a completed Reagents and Tools Table.

10) Digital image enhancement is acceptable practice, as long as it accurately represents the original data and conforms to community standards. If a figure has been subjected to significant electronic manipulation, this must be clearly noted in the figure legend and/or the 'Materials and Methods' section. The editors reserve the right to request original versions of figures and the original images that were used to assemble the figure. Finally, we generally encourage uploading of numerical as well as gel/blot image source data.

At EMBO Press we ask authors to provide source data for the main manuscript figures. You will receive a separate email with instructions for providing source data with your revised manuscript, including how to upload and organize the files.

In the interest of ensuring the conceptual advance provided by the work, we recommend submitting a revision within 3 months (25th Oct 2026). Please discuss the revision progress ahead of this time with the editor if you require more time to complete the revisions. Use the link below to submit your revision:

Link Not Available

Referee #2:

The authors have adequately addressed our comments and have substantially revised the text to improve the presentation of the data. In my opinion, the manuscript is now ready for publication.

Referee #3:

I have read this revised manuscript with great interest and find it substantially improved from the previous version. The following concerns remain and should be addressed.

Major concern

Overgeneralization to arabinosyl nucleoside analogues

My primary remaining concern is that the authors continue to extrapolate findings obtained exclusively with araATP and araCTP to arabinosyl nucleoside analogues as a class. This represents an improvement over the original submission, in which conclusions were generalized to all nucleoside analogues. However, the scope of the present study remains more limited than the language implies. It is not shown that araGTP and araUTP, neither of which is addressed in this manuscript, will exert the same effects on the replisome as araATP and araCTP. The authors should be careful not to imply otherwise.

Given that many readers encounter only the title and abstract, it is particularly important that these sections accurately reflect the scope of the experimental work. Accordingly, I request the following revisions:

Title: Replace the generic term "arabinosyl nucleoside analogue" with "araATP and araCTP", or otherwise make explicit that the study is restricted to these two compounds.

Abstract: The concluding sentence currently reads: "This study defines the initial response of the eukaryotic replisome to arabinosyl chemotherapies and may have implications for how resistance arises against them, and other nucleoside analogue chemotherapies, in the clinic." This sentence overstates the generality of the findings. I suggest revising it to: "This study defines the initial response of the eukaryotic replisome to araATP and araCTP chemotherapies and may have implications for how resistance arises against them, and other nucleoside analogue chemotherapies, in the clinic."

Line 84, page 3: The statement "we demonstrated that removal of ara analogues from nascent strand..." implies that all ara analogues behave equivalently, which is not supported by the literature or by the data presented here. Please revise to specify araA and araC. More broadly, I ask that the authors review the manuscript in its entirety and ensure that generalizations to ara analogues as a class are either removed or clearly flagged as assumptions rather than conclusions.

I am satisfied with all other revisions and responses provided by the authors.

We are very grateful to the editors and referees for their time in critically assessing our revised manuscript. Each comment has been addressed in our point-by-point responses below. The new manuscript has been revised accordingly, with any text changes tracked.

Referee #2:

The authors have adequately addressed our comments and have substantially revised the text to improve the presentation of the data. In my opinion, the manuscript is now ready for publication.

Referee #3:

I have read this revised manuscript with great interest and find it substantially improved from the previous version. The following concerns remain and should be addressed.

Major concern

Overgeneralization to arabinosyl nucleoside analogues

My primary remaining concern is that the authors continue to extrapolate findings obtained exclusively with araATP and araCTP to arabinosyl nucleoside analogues as a class. This represents an improvement over the original submission, in which conclusions were generalized to all nucleoside analogues. However, the scope of the present study remains more limited than the language implies. It is not shown that araGTP and araUTP, neither of which is addressed in this manuscript, will exert the same effects on the replisome as araATP and araCTP. The authors should be careful not to imply otherwise.

Given that many readers encounter only the title and abstract, it is particularly important that these sections accurately reflect the scope of the experimental work. Accordingly, I request the following revisions:

Title: Replace the generic term "arabinosyl nucleoside analogue" with "araATP and araCTP", or otherwise make explicit that the study is restricted to these two compounds.

We have now amended the title to “Response of the replisome to araA and araC suggests a mechanism for arabinosyl nucleoside tolerance”.

Abstract: The concluding sentence currently reads: "This study defines the initial response of the eukaryotic replisome to arabinosyl chemotherapies and may have implications for how resistance arises against them, and other nucleoside analogue chemotherapies, in the clinic." This sentence overstates the generality of the findings. I suggest revising it to: "This study defines the initial response of the eukaryotic replisome to araATP and araCTP chemotherapies and may have implications for how resistance arises against them, and other nucleoside analogue chemotherapies, in the clinic."

We have now changed the abstract to specify that our results define the replisome response to araA and araC, and that they may have implications for other nucleoside analogue chemotherapies.

Line 84, page 3: The statement "we demonstrated that removal of ara analogues from nascent strand..." implies that all ara analogues behave equivalently, which is not supported by the literature or by the data presented here. Please revise to specify araA and araC. More broadly, I ask that the authors review the manuscript in its entirety and ensure that generalizations to ara analogues as a class are either removed or clearly flagged as assumptions rather than conclusions.

This has been changed as requested.

I am satisfied with all other revisions and responses provided by the authors.

EMBO Press Author Checklist

Corresponding Authors Names: Harry Orrin & Joseph T.P. Yeeles	
Journal Submitted to: The EMBO Journal	
Manuscript Number: EMBOJ-2026-124467	

USEFUL LINKS FOR COMPLETING THIS FORM
The EMBO Journal - Author Guidelines
EMBO Reports - Author Guidelines
Molecular Systems Biology - Author Guidelines
EMBO Molecular Medicine - Author Guidelines

Reporting Checklist for Life Science Articles (updated January
This checklist is adapted from Materials Design Analysis Reporting (MDAR) Checklist for Authors. MDAR establishes a minimum set of requirements in transparent reporting in the life sciences (see Statement of Task: 10.31222/osf.io/9sm4x). Please follow the journal's guidelines in preparing your
Please note that a copy of this checklist will be published alongside your article.

Abridged guidelines for figures
1. Data
The data shown in figures should satisfy the following conditions:
- ➞ the data were obtained and processed according to the field's best practice and are presented to reflect the results of the experiments in an accurate and unbiased manner.
- ➞ ideally, figure panels should include only measurements that are directly comparable to each other and obtained with the same assay.
- ➞ plots include clearly labeled error bars for independent experiments and sample sizes. Unless justified, error bars should not be shown for technical
- ➞ if n<5, the individual data points from each experiment should be plotted. Any statistical test employed should be justified.
- ➞ Source Data should be included to report the data underlying figures according to the guidelines set out in the authorship guidelines on Data

2. Captions
Each figure caption should contain the following information, for each panel where they are relevant:
- ➞ a specification of the experimental system investigated (eg cell line, species name).
- ➞ the assay(s) and method(s) used to carry out the reported observations and measurements.
- ➞ an explicit mention of the biological and chemical entity(ies) that are being measured.
- ➞ an explicit mention of the biological and chemical entity(ies) that are altered/varied/perturbed in a controlled manner.
- ➞ the exact sample size (n) for each experimental group/condition, given as a number, not a range;
- ➞ a description of the sample collection allowing the reader to understand whether the samples represent technical or biological replicates (including how many animals, litters, cultures, etc.).
- ➞ a statement of how many times the experiment shown was independently replicated in the laboratory.
- ➞ definitions of statistical methods and measures:
  - common tests, such as t-test (please specify whether paired vs. unpaired), simple χ2 tests, Wilcoxon and Mann-Whitney tests, can be unambiguously identified by name only, but more complex techniques should be described in the methods section;
  - are tests one-sided or two-sided?
  - are there adjustments for multiple comparisons?
  - exact statistical test results, e.g., P values = x but not P values < x;
  - definition of 'center values' as median or average;
  - definition of error bars as s.d. or s.e.m.

Please complete ALL of the questions below.
Select "Not Applicable" only when the requested information is not relevant for your study.

Materials

Newly Created Materials	Information included in the manuscript?	In which section is the information available? (Reagents and Tools Table, Materials and Methods, Figures, Data Availability Section)
New materials and reagents need to be available; do any restrictions apply?	Not Applicable	

Antibodies	Information included in the manuscript?	In which section is the information available? (Reagents and Tools Table, Materials and Methods, Figures, Data Availability Section)
For **antibodies** provide the following information:<br>- Commercial antibodies: RRID (if possible) or supplier name, catalogue number and or/clone number<br>- Non-commercial: RRID or citation	Yes	Reagents and Tools Table

DNA and RNA sequences	Information included in the manuscript?	In which section is the information available? (Reagents and Tools Table, Materials and Methods, Figures, Data Availability Section)
**Short novel DNA or RNA including primers, probes**: provide the sequences.	Yes	Appendix Table S1

Cell materials	Information included in the manuscript?	In which section is the information available? (Reagents and Tools Table, Materials and Methods, Figures, Data Availability Section)
**Cell lines:** Provide species information, strain. Provide accession number in repository **OR** supplier name, catalog number, clone number, and/**OR** RRID.	Not Applicable	
**Primary cultures:** Provide species, strain, sex of origin, genetic modification status.	Not Applicable	
Report if the cell lines were recently **authenticated** (e.g., by STR profiling) and tested for mycoplasma contamination.	Not Applicable	

Experimental animals	Information included in the manuscript?	In which section is the information available? (Reagents and Tools Table, Materials and Methods, Figures, Data Availability Section)
**Laboratory animals or Model organisms:** Provide species, strain, sex, age, genetic modification status. Provide accession number in repository **OR** supplier name, catalog number, clone number, **OR** RRID.	Yes	Reagents and Tools Table
**Animal observed in or captured from the field:** Provide species, sex, and age where possible.	Not Applicable	
Please detail **housing and husbandry conditions**.	Not Applicable	

Plants and microbes	Information included in the manuscript?	In which section is the information available? (Reagents and Tools Table, Materials and Methods, Figures, Data Availability Section)
**Plants:** provide species and strain, ecotype and cultivar where relevant, unique accession number if available, and source (including location for collected wild specimens).	Not Applicable	
**Microbes:** provide species and strain, unique accession number if available, and source.	Not Applicable	

Human research participants	Information included in the manuscript?	In which section is the information available? (Reagents and Tools Table, Materials and Methods, Figures, Data Availability Section)
If collected and within the bounds of privacy constraints report on age, sex and gender or ethnicity for all study participants.	Not Applicable	

Core facilities	Information included in the manuscript?	In which section is the information available? (Reagents and Tools Table, Materials and Methods, Figures, Data Availability Section)
If your work benefited from core facilities, was their service mentioned in the acknowledgments section?	Yes	Acknowledgements

Design

Study protocol	Information included in the manuscript?	In which section is the information available? (Reagents and Tools Table, Materials and Methods, Figures, Data Availability Section)
If study protocol has been **pre-registered, provide DOI in the manuscript**. For clinical trials, provide the trial registration number **OR** cite DOI.	Not Applicable	
Report the **clinical trial registration number** (at ClinicalTrials.gov or equivalent), where applicable.	Not Applicable	

Laboratory protocol	Information included in the manuscript?	In which section is the information available? (Reagents and Tools Table, Materials and Methods, Figures, Data Availability Section)
Provide DOI OR other citation details if **external detailed step-by-step protocols** are available.	Not Applicable	

Experimental study design and statistics	Information included in the manuscript?	In which section is the information available? (Reagents and Tools Table, Materials and Methods, Figures, Data Availability Section)
Include a statement about **sample size** estimate even if no statistical methods were used.	Not Applicable	
Were any steps taken to minimize the effects of subjective bias when allocating animals/samples to treatment (e.g. **randomization procedure**)? If yes, have they been described?	Not Applicable	
Include a statement about **blinding** even if no blinding was done.	Not Applicable	
Describe **inclusion/exclusion criteria** if samples or animals were excluded from the analysis. Were the criteria pre-established?<br><br>If sample or data points were omitted from analysis, report if this was due to attrition or intentional exclusion and provide justification.	Not Applicable	
For every figure, are **statistical tests** justified as appropriate? Do the data meet the assumptions of the tests (e.g., normal distribution)? Describe any methods used to assess it. Is there an estimate of variation within each group of data? Is the variance similar between the groups that are being statistically compared?	Yes	Figure legends/ Materials and Methods/ Appendix Table S4

Sample definition and in-laboratory replication	Information included in the manuscript?	In which section is the information available? (Reagents and Tools Table, Materials and Methods, Figures, Data Availability Section)
In the figure legends: state number of times the experiment was **replicated** in laboratory.	Yes	Figure legends/ Materials and Methods
In the figure legends: define whether data describe **technical or biological replicates**.	Yes	

Ethics

Ethics	Information included in the manuscript?	In which section is the information available? (Reagents and Tools Table, Materials and Methods, Figures, Data Availability Section)
Studies involving **human participants**: State details of **authority granting ethics approval** (IRB or equivalent committee(s), provide reference number for approval.	Not Applicable	
Studies involving **human participants**: Include a statement confirming that **informed consent** was obtained from all subjects and that the experiments conformed to the principles set out in the WMA Declaration of Helsinki and the Department of Health and Human Services Belmont Report.	Not Applicable	
Studies involving **human participants:** For publication of **patient photos**, include a statement confirming that consent to publish was obtained.	Not Applicable	
Studies involving experimental **animals**: State details of **authority granting ethics approval** (IRB or equivalent committee(s), provide reference number for approval. Include a statement of compliance with ethical regulations.	Not Applicable	
Studies involving **specimen and field samples:** State if relevant **permits** obtained, provide details of authority approving study; if none were required, explain why.	Not Applicable	

Dual Use Research of Concern (DURC)	Information included in the manuscript?	In which section is the information available? (Reagents and Tools Table, Materials and Methods, Figures, Data Availability Section)
Could your study fall under dual use research restrictions? Please check biosecurity documents and list of **select agents and toxins** (CDC): https://www.selectagents.gov/sat/list.htm	Not Applicable	
If you used a select agent, is the security level of the lab appropriate and reported in the manuscript?	Not Applicable	
If a study is subject to dual use research of concern regulations, is the name of the **authority granting approval and reference number** for the regulatory approval provided in the manuscript?	Not Applicable	

Reporting

The MDAR framework recommends adoption of discipline-specific guidelines, established and endorsed through community initiatives. Journals have their own policy about requiring specific guidelines and recommendations to complement MDAR.

Adherence to community standards	Information included in the manuscript?	In which section is the information available? (Reagents and Tools Table, Materials and Methods, Figures, Data Availability Section)
State if relevant guidelines or checklists (e.g., **ICMJE, MIBBI, ARRIVE, PRISMA**) have been followed or provided.	Not Applicable	
For **tumor marker prognostic studies,** we recommend that you follow the **REMARK** reporting guidelines (see link list at top right). See author guidelines, under 'Reporting Guidelines'. Please confirm you have followed these guidelines.	Not Applicable	
For **phase II and III randomized controlled trials**, please refer to the **CONSORT** flow diagram (see link list at top right) and submit the CONSORT checklist (see link list at top right) with your submission. See author guidelines, under 'Reporting Guidelines'. Please confirm you have submitted this list.	Not Applicable	

Data Availability

Data availability	Information included in the manuscript?	In which section is the information available? (Reagents and Tools Table, Materials and Methods, Figures, Data Availability Section)
Have **primary datasets** been deposited according to the journal's guidelines (see 'Data Deposition' section) and the respective accession numbers provided in the Data Availability Section?	Not Applicable	
Were **human clinical and genomic datasets** deposited in a public access-controlled repository in accordance to ethical obligations to the patients and to the applicable consent agreement?	Not Applicable	
Are **computational models** that are central and integral to a study available without restrictions in a machine-readable form? Were the relevant accession numbers or links provided?	Not Applicable	
If publicly available data were reused, provide the respective **data citations in the reference list**.	Not Applicable	
